# Identification of novel non-toxic and anti-angiogenic α-fluorinated chalcones as potent colchicine binding site inhibitors

**DOI:** 10.1080/14756366.2021.2014831

**Published:** 2022-01-03

**Authors:** Moran Sun, Minghua Yuan, Yingying Kang, Jinling Qin, Yixin Zhang, Yongtao Duan, Longfei Wang, Yongfang Yao

**Affiliations:** a Henan Provincial Key Laboratory of Children's Genetics and Metabolic Diseases, Children's Hospital Affiliated to Zhengzhou University, Zhengzhou University, Zhengzhou, China; b Key Laboratory of Advanced Drug Preparation Technologies, School of Pharmaceutical Sciences, Ministry of Education, Zhengzhou University, Zhengzhou, China;; c State Key Laboratory of Esophageal Cancer Prevention & Treatment, School of Pharmaceutical Sciences, Zhengzhou University, Zhengzhou, China

**Keywords:** Microtubules, zebrafish, angiogenesis, chalcone, fluorinated

## Abstract

α-Fluorinated chalcones were prepared and evaluated for their cell growth inhibitory properties against six human cancer cell lines. The most potent chalcone **4c** demonstrated excellent selective toxicity against cancer cells versus normal human cells, with IC_50_ values at nanomolar concentration ranges against 5 cancer cell lines. A further study revealed that **4c** could bind to the colchicine site of tubulin, disrupt the cell microtubule networks, and effectively inhibit tubulin polymerisation. Cellular-based mechanism studies elucidated that **4c** arrested MGC-803 cell cycle at G2/M phase. In addition, **4c** dose-dependently caused Caspase-induced apoptosis of MGC-803 cells through mitochondrial dysfunction. Notably, compound **4c** was found to inhibit the HUVECs tube formation, migration, and invasion *in vitro*. Furthermore, our data suggested that treatment with **4c** significantly reduced MGC-803 cells metastasis and proliferation *in vitro*. Overall, this work showed that chalcone hybrid **4c** is a potent inhibitor of tubulin assembly with prominent anti-angiogenesis and anti-cancer properties.

## 1. Introduction

Cancer is one of the most formidable afflictions, which represents a leading cause of death worldwide^1^. Drug resistance, side effects and lack of efficacy associated with current cancer chemotherapy demand the design of new and safer anticancer drugs^2^. Vascular Disrupting Agents (VDAs) constitute an innovative and complementary approach to other standard anticancer therapies, due to the low toxicity to the normal cell, the broad-spectrum of activity against tumours, and tolerance to the development of resistance^3^. These compounds have been found to cause a rapid and selective shutdown in tumour blood flow to solid tumours, while the blood flow in normal tissues was leaved to be relatively intact. As a result, the endothelial cells of immature vasculature shutdown and blood flow within the tumour rapidly and dramatically reduced. Finally, extensive tumour necrosis will occur due to starved of oxygen and nutrients^4^.

**Figure 1.**
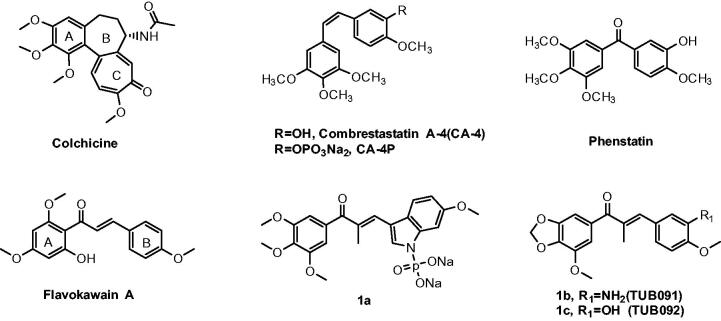
Representive tubulin inhibitors with anti-angiogenesis potency.

The most studied group of VDAs is microtubule-destabilizing agents that interact at the colchicine-binding site of the αβ-tubulin dimer^5^. Compared with other site binders, colchicine-site binders have several advantages including simpler structure, low toxicity, improved aqueous solubility and fewer issues of multidrug resistance^6^. The normal function of endothelial cells, including movement, invasion, attachment, arrangement, and proliferation, is highly dependent on the tubulin cytoskeleton. It has been shown that endothelial cells of immature vasculature have a less developed actin cytoskeleton and are therefore more sensitive to the effects of VDAs. Consequently, the vascular targeting properties of VDAs were invariably linked to the microtubule-binding properties of the compound. In general, colchicine-site binders have anti-angiogenesis or disrupting established tumour vasculature effects or both^7^. Combretastatin A4(CA-4), a representative colchicine-site binder, exhibit prominent anti-angiogenic property within solid tumour, suggesting that this class of compounds could target the tumour vasculature and ultimately lead to tumour necrosis (Figure 1)^8^. The water-soluble phosphate prodrug CA-4P (Zybrestat ^TM^) has been approved as an orphan drug for anaplastic thyroid cancer treatment by FDA^9^. However, *Z*-natural stilbene compound CA-4 is prone to isomerise to the more stable *E*-isomer during storage and administration, which displayed a dramatically reduced activity.

CA-4 has provided a structural model for the design of new analogs with anti-vascular and anticancer profiles. Chalcones, bearing an α, β-unsaturated carbonyl moiety, can be viewed simplistically as keto-stilbenes and mimics a portion of CA-4^10^. Accordingly, chalcones have been used as a source of microtubule-destabilizing agents binding at the colchicine-site with the purpose to improve the chemical stability of CA-4. Flavokawain A was a promising apoptosis inducer and arrested cell cycle at G2/M against HER2-overexpressing breast cancer cells (Figure 1)^10^. Incorporated the aryl substitution pattern of CA-4 into chalcones afford compound **1a**, which displayed low cytotoxicity towards normal cells and greater metabolic stability than CA-4, along with potent antiproliferative activity against drug resistant cells^11^. Chalcone **1b** (TUB091) exhibit vascular disrupting effects and anti-metastatic activity similar to that of CA-4P^12^.

The introduction of fluorine atoms into a bioactive molecule might affect the binding affinity with the target protein, pKa value, lipid solubility, metabolic pathways, molecular conformation and pharmacokinetic properties^13^. For a specific molecule, the biological effect brought by fluorine substitution is difficult to predict. Therefore, in the process of drug optimisation, "fluorine scan" has become a fundamental routine work. Furthermore, the benzoheterocycle has assumed special significance in pharmaceutical chemistry and serve as unique versatile scaffolds for drug design^14^. In continuation of our efforts to discover and develop novel bioactive molecules that target the tubulin-microtubule system^15^, we have incorporated a fluorine atom in the α-position of chalcone and substituted one of the two phenyl rings with benzoheterocycle nucleus. In all cases, as a feature structure of tubulin-binding agents, the typical 3,4,5-trimethoxyphenyl of CA-4 was retained in order to achieve the outstanding compounds. Herein, we would like to report full details of the synthesis, characterisation, and evaluation of their tumour growth inhibition against cancer cells. The lead compound **4c** was also elucidated for its fundamental cytotoxic mechanisms, anti-angiogenesis, and anti-metastatic properties *in vitro* and *in vivo*.

## 2. Results and discussion

### 2.1. Chemistry

**Scheme 1.**
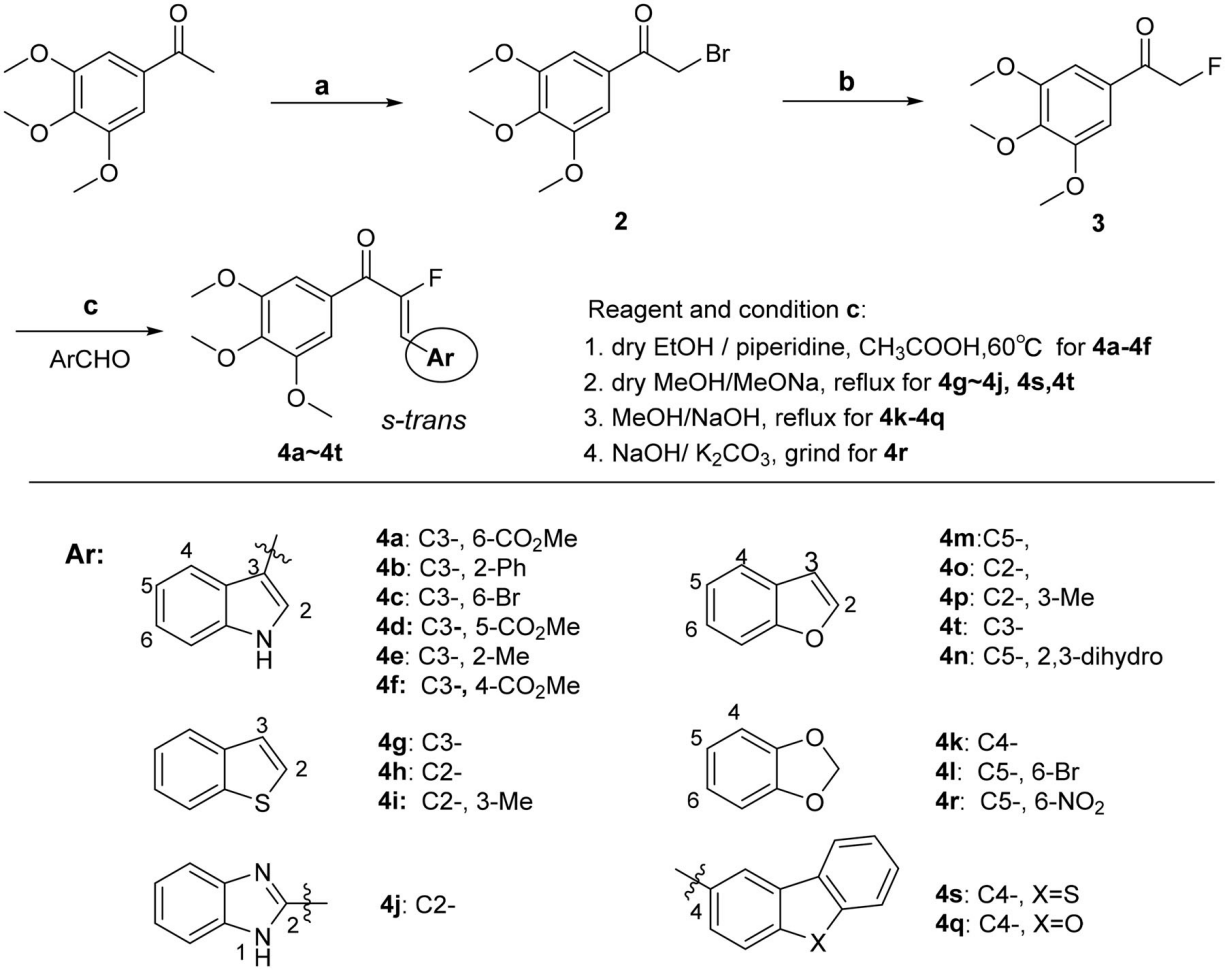
Reagents and conditions: (a) TBABr_3_, DCM: MeOH = (5:2), rt, overnight; (b) KF,18-crown-6, MeCN; (c) MeOH or ethanol or THF or Dioxane, appropriate base, 0–75 °C.

A novel series of chalcone derivatives containing α-fluoro moiety was synthesised using the procedure described in Scheme 1. The intermediate **2** was obtained by bromination at the α-position of starting material 3,4,5-trimethoxyacetophenone with tetrabutylammonium tribromide. The resulting bromide **2** was subjected to nucleophilic substitution reaction, affording fluoride **3** in the presence of potassium fluoride in dry acetonitrile. A Knoevenagel condensation between intermediate **3** and a wide range of heterocyclic aromatic aldehyde was promoted by appropriate base to provide the target chalcone analogues **4a–4t**. It should be noted the amount of base may be range from 0.01 to 100 equivalents.

### 2.2. Anti-cancer activity

#### 2.2.1. Antiproliferative activity and SARs

To discover novel tubulin polymerisation inhibitors, we initially determined the anti-proliferative activity of all synthesised compounds **4a–4t** against MCF-7 (human breast cancer cells), MGC-803 (human gastric cancer cells), HepG2 (human liver cancer cells), Hela (human cervical cancer cells), A549 (human non-small cell lung cancer) and U937 (human leukaemia cells) cell lines using the SRB and CCK-8 assay, while the colchicine site tubulin polymerisation inhibitor CA-4 was used as a positive control. The results listed in Table 1 indicated that this series of chalcones analogues exhibited decent growth-inhibitory activity.

**Table 1.** Antiproliferative activities of all target compounds against different human cell lines

Compd	IC_50_±SD (µM)^a^					
	A549	Hela	MCF-7	U937	MGC-803	HepG2
**4a**	>5	>5	>5	>5	>5	>5
**4b**	3.635 ± 0.001	0.685 ± 0.002	0.551 ± 0.022	0.562 ± 0.020	0.202 ± 0.023	0.883 ± 0.041
**4c**	0.202 ± 0.012	0.025 ± 0.001	0.254 ± 0.037	0.025 ± 0.001	0.0571 ± 0.006	>5
**4d**	>5	>5	>5	>5	>5	>5
**4e**	>5	0.101 ± 0.031	0.551 ± 0.014	0.123 ± 0.011	0.072 ± 0.002	1.385 ± 0.001
**4f**	>5	>5	>5	>5	>5	>5
**4g**	0.505 ± 0.010	0.072 ± 0.001	0.121 ± 0.012	0.456 ± 0.013	0.021 ± 0.007	0.199 ± 0.006
**4h**	2.569 ± 0.159	2.493 ± 0.002	2.101 ± 0.003	0.606 ± 0.026	1.897 ± 0.001	5.976 ± 0.004
**4i**	>5	>5	>5	>5	>5	>5
**4j**	>5	>5	>5	>5	>5	>5
**4k**	>5	0.565 ± 0.012	0.441 ± 0.001	0.806 ± 0.024	0.166 ± 0.004	3.298 ± 0.007
**4l**	>5	>5	>5	>5	>5	>5
**4m**	>5	0.925 ± 0.007	1.002 ± 0.007	0.710 ± 0.027	0.362 ± 0.123	1.407 ± 0.014
**4n**	0.364 ± 0.003	0.042 ± 0.001	0.128 ± 0.004	0.031 ± 0.001	0.043 ± 0.001	0.116 ± 0.001
**4o**	>5	>5	4.408 ± 0.043	>5	3.301 ± 0.026	>5
**4p**	>5	>5	>5	>5	>5	>5
**4q**	>5	>5	>5	>5	>5	>5
**4r**	>5	>5	>5	>5	>5	>5
**4s**	>5	>5	>5	>5	>5	>5
**4t**	1.025 ± 0.064	0.203 ± 0.006	1.293 ± 0.007	0.125 ± 0.002	N.D.^b^	1.063 ± 0.008
**CA-4**	0.005 ± 0.001	0.008 ± 0.001	0.011 ± 0.001	0.0003 ± 0.001	0.002 ± 0.001	0.007 ± 0.001

^a^IC_50_ values are presented as the mean ± SD(standard deviation) from three separated experiments. ^b^N. D.: not determined.

We synthesised six indole ring-B chalcone derivatives, of which **4b** (2-phenyl) and **4e** (2-methyl) showed comparable antiproliferative activity, while **4c** (6-bromide) exhibited the most promising and broad-spectrum anti-tumour cell proliferation activity among all tested compounds, with IC_50_ values of 0.025 µM against Hela and U937, respectively. However, the introduction of *t* -CO_2_Me group at the 6-, 5- and 4-positions of indoles (**4a**, **4d,** and **4f**) resulted in a significant decrease in the anti-proliferative activity (IC_50_ > 5 µM), suggesting that the donating/withdrawing properties and steric hinderance of the substituents on ring-B might play a comprehensive role on the inhibitory activity. Comparing three benzothiophene ring-B chalcones (**4g**, **4h**, and **4i)**, it was found that the activity was significantly affected by the position of benzothiophene attached to α, β-unsaturated ketone and the tendency of the inhibition activity is 3-position >2-position. Compared with the parent compound **4h**, chalcone **4i** containing methyl substation at 3-position showed diminished cytotoxic activity towards all tested cell lines. Interestingly, this SARs were also valid in benzofuran series, namely, chalcone with an CH_3_ group at the 3-position (**4p**) caused a remarkable decrease in antiproliferative activity, and the relationships between the location of heterocycles and the antiproliferative activities were still 3-position(comparable to 5-position) >2-position. Among all benzofuran chalcone derivatives, compound **4n** (2,3-dihydro-benzofuran ring-B) had a more potent inhibitory effect (IC_50_=0.031–0.364 µM) towards several tested cancer cell lines. From the comparison of **4k** (C4) with the inactive analogues **4l** (C5, 6-Br) and **4r** (C5, 6-NO_2_), we could further surmise that the linkage position of ring-B on α, β-unsaturated ketone may play a more critical role in anti-proliferation effect than properties of substituent on ring-B, such as electronegativity and atom size. Based on these findings, compound **4c** was selected as the optimised compound for further studies, and to prove its mode of action.

#### 2.2.2. Analysis of tubulin polymerisation in vitro

**Figure 2.**
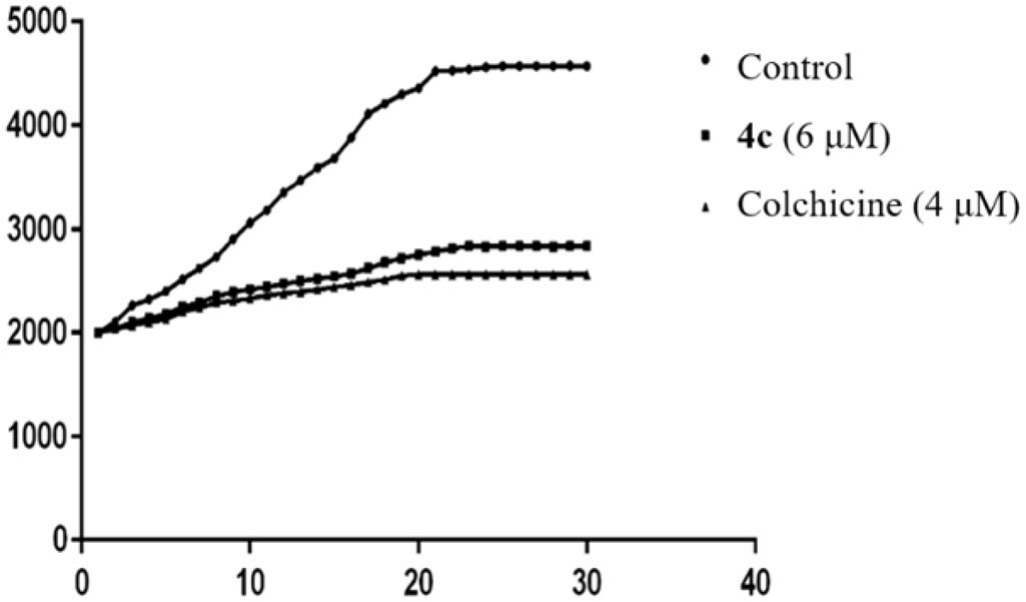
Effect of **4c** on tubulin polymerisation in vitro. Data are presented as the mean ± SD from three independent experiments.

Among chalcones containing α- fluoro moiety mentioned above, **4c** showed the best activity against six cell lines with an IC_50_ value of 0.025–0.254 µM. Accordingly, **4c** was chosen to undergo the microtubule polymerisation assay *in vitro* and to demonstrate whether these α-fluoro chalcone derivatives are tubulin targeting agents. Colchicine, a microtubule-destabilizing agent (MDA), is known to significantly inhibit self-polymerisation of tubulin. The microtubule kinetic experiments were performed with 4 µM of colchicine as a positive control and 6 µM of **4c** as the experimental group according to the method originally described by J. M. Andreu et al. with some modifications^16^. Purified and unpolymerised tubulin was incubated with the tested compounds and the increased tendency of fluorescence intensity shown in control samples was obviously slowed down. As shown in Figure 1, compound **4c** exhibited a similar action to that of colchicine, which suggest that **4c** could be used as a potential MDA for cancer therapy (Figure 2).

#### 2.2.3. The selectivity of 4c towards normal cells

The effect of compound **4c** on three types of normal human cell lines including LO2 (human hepatocyte cells), 293 T (human embryonic kidney cell) and THP-1-derived macrophages was then determined. In contrast with the positive control CA-4, **4c** showed low toxicity to non-malignant cell lines especially macrophages with an IC_50_ value of 1210 nΜ and the selectivity ratio towards MGC-803 cells and macrophage cells was up to 21.2-folds (Table 2).

**Table 2.** Growth inhibitory effects of **4c** on macrophage.

Compound	IC_50_±SD(nM)^a^				Selectivity ratio^b^
	MGC-803	LO2	293T	macrophage	
**4c**	57 ± 6	680 ± 220	1050 ± 220	1210 ± 17	11.9 ∼ 21.2
**CA-4**	2 ± 1	5 ± 1	12 ± 2	7 ± 1	2.5 ∼ 6.0

^a^Each compound was tested in triplicate; the data are presented as the mean ± SD.

^b^Selectivity ratio = (IC_50_ human normal cells)/(IC_50_ MGC-803).

#### 2.2.4. Docking profiles of compound 4c with tubulin

**Figure 3.**
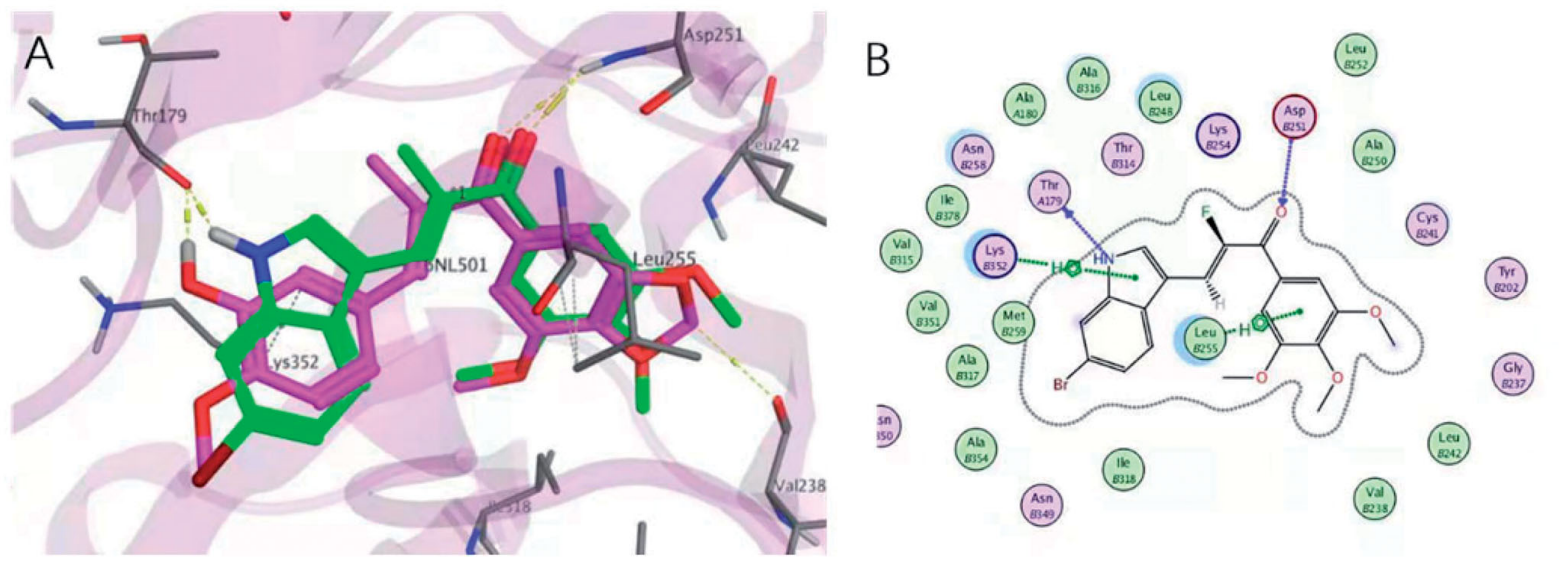
Proposed binding models for **4c** (green) with tubulin (PDB code：5JVD). (A) The superimposed conformation with TUB092(carmine). The hydrogen bonds were shown in yellow dashed lines. (B) The 2 D interactions between 4c and tubulin.

In order to explore the interaction mode of compound **4c** with the colchicine binding sites of tubulin, molecular docking study was performed by using MOE (PDB code:5JVD). It should be noted that chalcones with a fluorine atom in the α-position have been shown by X-ray analysis to adopt the *s-trans* conformation, typical of α-methyl chalcones^12^. As shown in Figure 3(A), the superimposition of the best poses of compound **4c** with the original ligand TUB092(α-methyl chalcone) in the colchicine binding pocket clearly demonstrated that **4c** adopt almost identical binding position to that of TUB092. The 3,4,5-trimethoxyphenyl moiety of **4c** was well buried into the hydrophobic pocket shaped by the side chains of *β*-tubulin residues Leu252, Ala250, Cys241, Leu242 and Ile318. The N atom of indole ring and carbonyl of **4c** formed a hydrogen bond with residue *α*Thr179 and *β*Lys352 respectively, which was resembled with that of 4′-methoxy oxygen at ring-B and carbonyl of TUB902. Meanwhile, 2 D analysis of the interactions revealed that a CH-π interaction could be formed between the ring-A of **4c** and *β*Leu255, which is highly consistent with that of TUB092. In terms of **4c**, further stabilisation is provided by a cation-π interaction between the indole ring and the side chain of *β*Lys 352. This non-polar force together with hydrogen bonds strongly help compound **4c** to anchor in a dominant conformation in the binding site of tubulin and these results can describe the inhibitory effect of compound **4c**.

#### 2.2.5. Anti-microtubule effects in MGC-803 cancer cells

**Figure 4.**
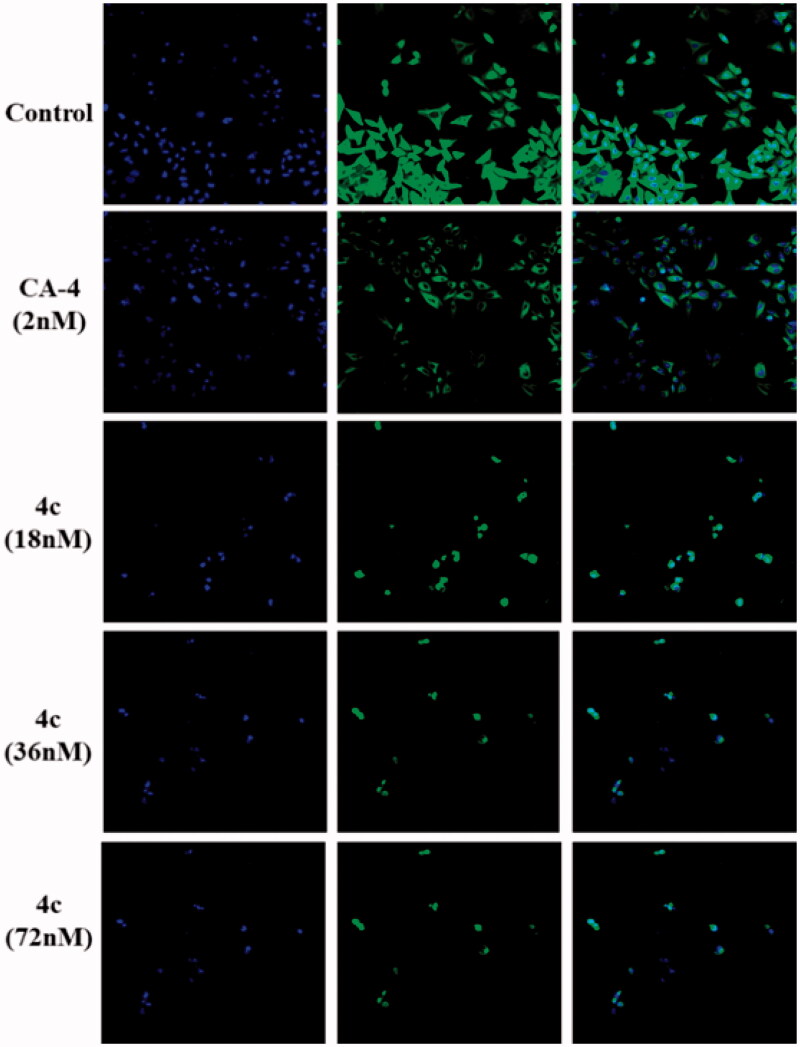
Effects of **4c** on the cellular microtubule network visualised by immunofluorescence.

Since tubulin-microtubule system plays a crucial role in mechanical solidity, cell motility and mitosis, an immunofluorescent staining assay was further carried out in MGC-803 cells to evaluate the inhibitory effects of **4c** on microtubule cytoskeleton. The morphological changes of microtubules were observed by laser confocal microscopy, as shown in Figure 4. In the solvent-treated control, the intracellular network of microtubules assembled regularly and formed normal intact network with fine filaments, whereas, cells treated with **4c** at different concentrations (0, 18, 36, and 72 nΜ) led to remarkable disruption of microtubule reticular network. Even at concentration as low as 18 nm, the microtubule spindle shrunk around the centre of the cells and the cellular structure was destroyed distinctly, which is very similar to positive control microtubule-destabilizing agent CA-4. Upon treatment with higher concentrations of **4c**, the dispersed tubulin dimers were even more obvious and there is no tubular structure left. Such morphological microtubules changes indicated that compound **4c** destroyed the microtubule organisation, thus preventing the mitosis of MGC-803 at much lower effective concentration, which might eventually induce cell apoptosis.

#### 2.2.6. Cell cycle analysis

**Figure 5.**
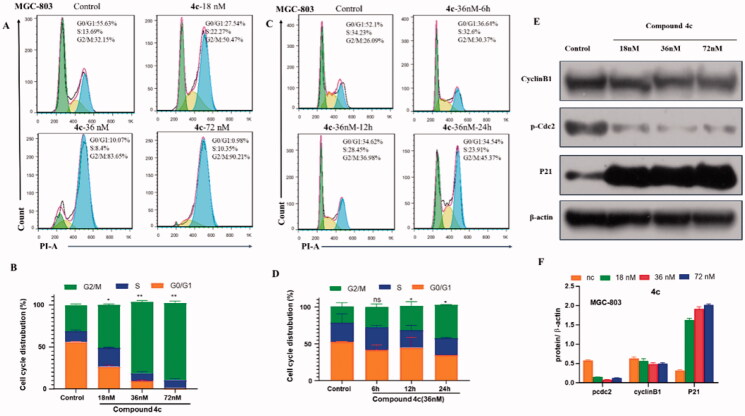
Compound **4c** induced G2/M arrest in MGC-803 cancer cells. (A) The percentages of cells at different phase of the cell cycle after treatment with various concentrations of **4c**. (B) Histograms display the percentage of cell cycle distribution. (C) The percentage of different phase of the cell cycle after treatment with the same concentration for different times. (D) Histograms display the percentage of cell cycle distribution for different times. (E) Western blotting analysis on the effect of **4c** on the G2/M regulatory proteins. (F) Histograms display the density rations of p-Cdc2, CyclinB1 and P21.

Most inhibitors of microtubule polymerisation are able to block mitosis in G2/M phase, which ultimately leads to apoptosis by disrupting tubulin-microtubule balance^17^. To elucidate whether the cytotoxicity of **4c** was due to cell cycle arrest, flow cytometry analysis and PI marking method were used to evaluate the effects of **4c** on the MGC-803 cell cycle distribution. As presented in Figure 5(A,B), treatment of **4c** at concentrations of 18, 36, 72nΜ caused a rise of cells in G2/M population from 32.15% to 50.47%, 83.65% and 90.21% respectively, indicating that **4c** arrested MGC-803 cell cycle at the G2/M phase. A significant difference was also observed when we further evaluated its effects using different time points (Figure 5(C,D)). MGC-803 cells treated with **4c** (36 nM) can be arrested in the G2/M phase from 30.37% at 6 h to 45.37% at 24 h.

Cdc2 kinase, a member of Ser/Thr protein family, is considered as a driving force of G2/M transition. Activation of Cdc2 catalysed the phosphorylation of a variety of substrate proteins, thus promoting cell transition from G2 phase to M phase^18^. Cyclin B1 plays a vital role to control cell transition from G2 to M phase^19^. P21 also effectively regulates cell cycle progress by increasing the intracellular G2/M phase ratio. Therefore, the expression of cell cycle checkpoint protein was investigated. Figure 4(E,F) showed that chalcone **4c** decrease the phosphorylation levels of Cdc2 in a concentration-dependent manner. We further observed that **4c** caused a decrease in Cyclin B1 and increased the expression of tumour suppressor protein P21, which confirmed a G/M phase arrest by **4c**. These results were consistent with flow cytometry analysis and microtubule immunofluorescence assay, which confirmed the mechanism of the cell cycle blocking effect at the cellular biochemical level.

#### 2.2.7. Cell apoptosis assay

**Figure 6.**
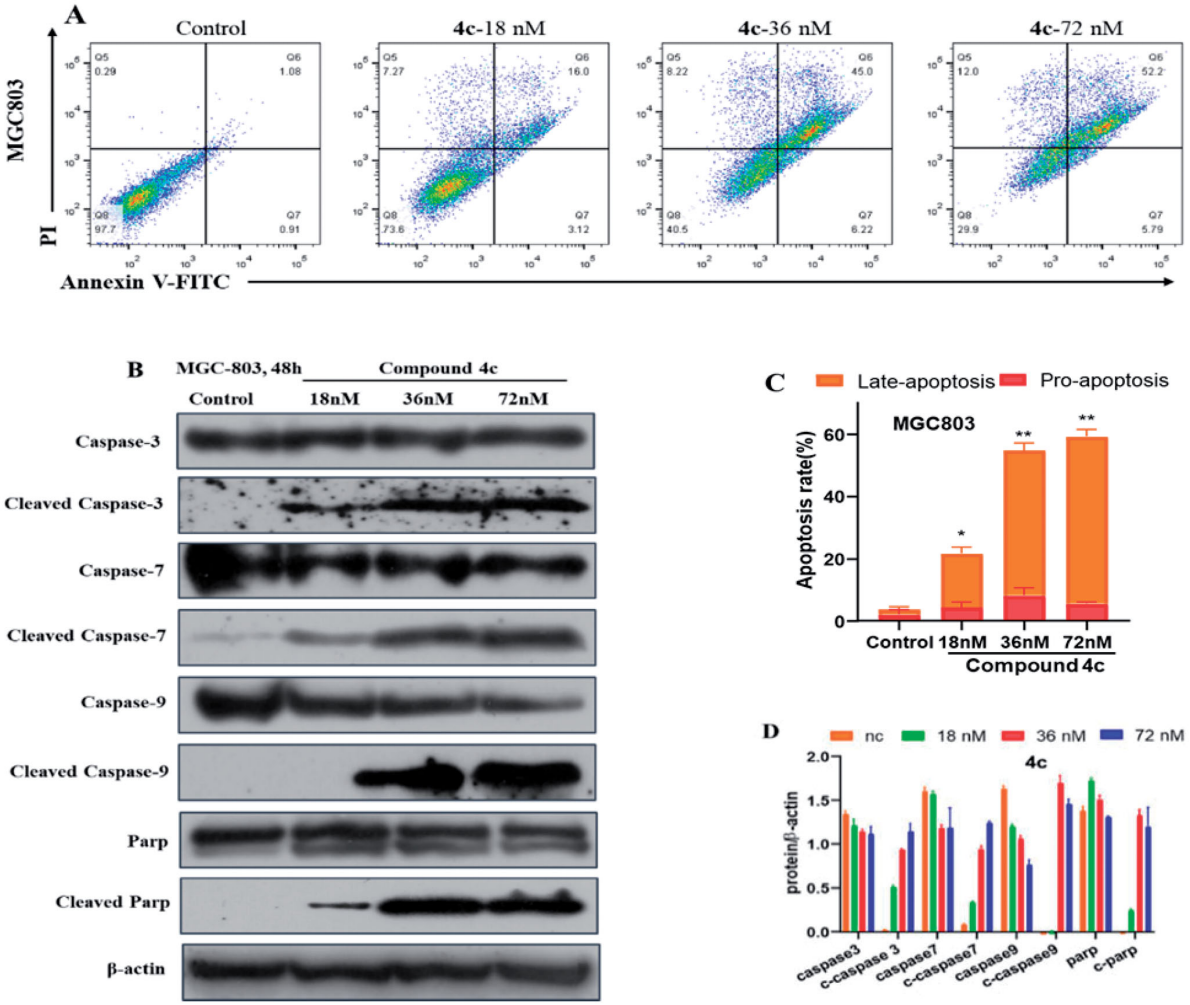
Compound **4c** induced apoptosis in MGC-803 cancer cells. (A) MGC-803cells stained with Annexin V/PI, followed by flow cytometric analysis. (B) Western blot analysis of apoptosis-related proteins. (C) Histograms display the percentage of apoptosis cells. (D) Histograms display the density ratios of Caspase -3/-7/-9, cleaved Caspase -3/-7/-9, Parp and cleaved Parp.

According to above-mentioned cell cycle analysis, we suppose that **4c** might induce cells apoptosis, like other microtubule polymerisation inhibitors. Thus, the effects of **4c** on MGC-803 cell apoptosis were examined by flow cytometry. As shown in Figure 6(A,C), after treating MGC-803 cells with **4c** (18, 36, 72nΜ) for 48 h, flow analysis showed that **4c** induced apoptosis in a dose-dependent manner, with the percentage of apoptotic cells ranging from 19.12% to 57.99%, compared with 1.99% in the control group.

The Caspase family proteins are considered to be critical regulators of cell apoptosis, in which Caspase-3 is one of the most important "executioner" of apoptosis induction, capable of cleaving many important cellular matrices and represent the hallmark of apoptosis^20^. To explore the apoptosis-associated protein alterations, the expression of Caspase -3/-7/-9 and PARP was analysed. As presented in Figure 6(B,D), MGC-803 cells was treated with **4c** at different concentrations (18, 36 and 72 nM) for 48 h, an up-regulation of cleaved Caspase-3/-7/-9, the activated form of Caspase family apoptotic proteins, was observed. **4c** treatment resulted in a cleavage of Caspase-9, indicating that it activated the exogenous apoptotic pathway and further processed other Caspase members such as Caspase-3 and -7, thereby initiating the Caspase cascade. As a result of these alteration, its substrate protein PARP, a marker of nuclear fragmentation and chromatin condensation, was cleaved and activated. These results demonstrated that compound **4c** could trigger the Caspase cascade and the cleavage of PARP, eventually inducing apoptosis of MGC-803 cells.

#### 2.2.8. *In vitro* evaluation of mitochondrial membrane potential (MMP) and reactive oxygen species (ROS) generation

**Figure 7.**
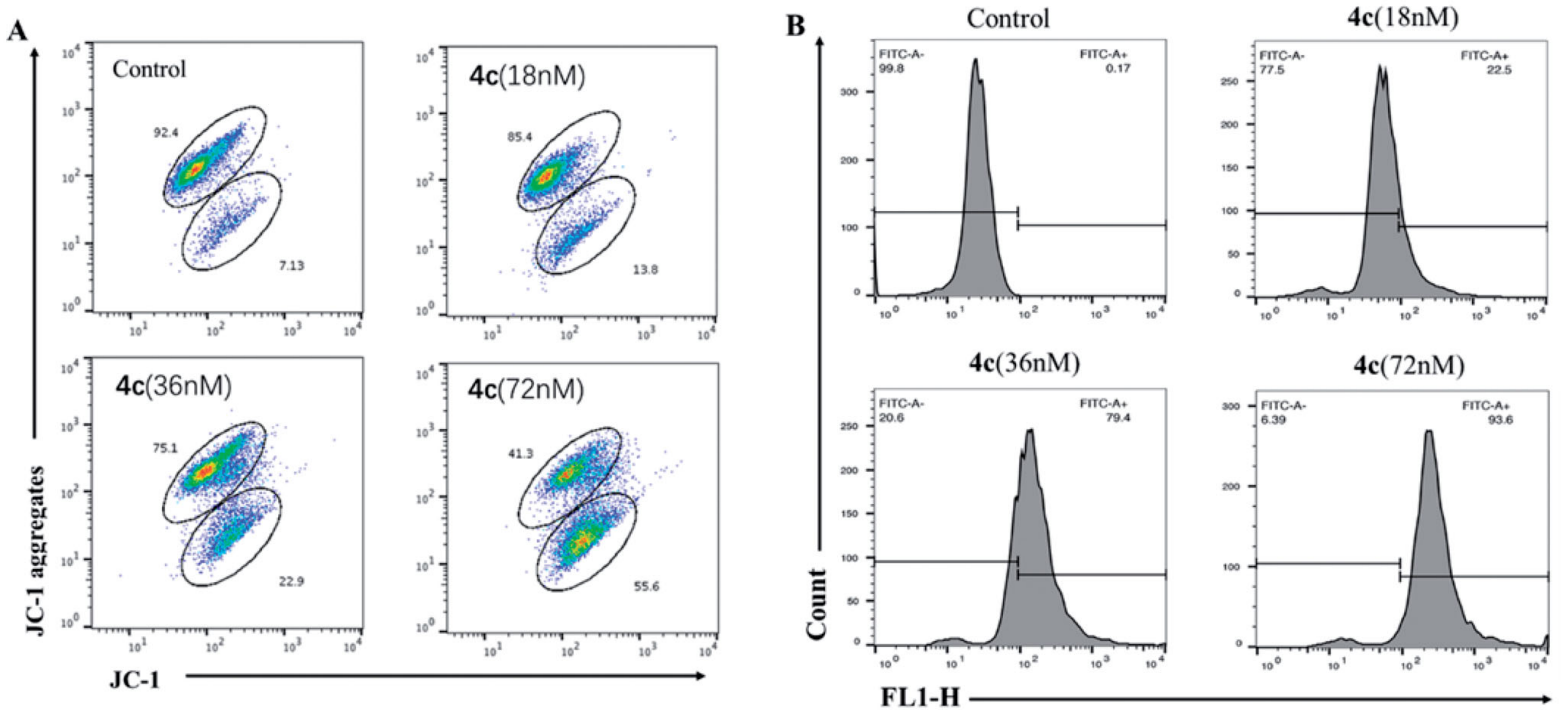
(A) Effects of **4c** on the MMP of MGC-803 cells; (B) The generation of ROS was measured using DCFH-DA.

There is a close relationship between mitochondria and apoptosis, and a decrease in mitochondrial membrane potential is one of the irreversible events that occur early in apoptosis cells. Therefore, effect of compound **4c** on MMP of MGC-803 cells was examined by cationic JC-1 staining method and flow cytometry. As illustrated in Figure 7(A), with the concentration of **4c** ranging from 0 to 72 nΜ, the red fluorescence intensity decreased from 92.4 to 41.3%, and the green fluorescence intensity increased from 7.13 to 55.6%, indicating that **4**c produced the collapse of MMP and eventually induced MGC-803 cell apoptosis.

It is now generally accepted that mitochondrial dysfunction is the main source of elevated ROS levels, accompanied by changes in mitochondrial membrane potential and activation of downstream Caspases. To further evaluate the alterations of intracellular ROS levels after cells exposed to **4c**, flow cytometry was analysed with 2′,7′-Dichlorodihydrofluorescein diacetate (DCFH-DA) as fluorescent probe. As shown in Figure 7(B), **4c** induced intracellular ROS generation in a dose-dependent manner. The ratio of DCF positive cells was increased from 0.17% (control) to 93.6% in cells incubated with 72 nM of **4c**. According to above-mentioned results, we can deduce that **4c** induced cell apoptosis through a variety of ways including the disruption of the mitochondrial membrane potential, accumulation of intracellular ROS level and regulation of apoptosis-related proteins expression in cancer cells.

#### 2.2.9. Migration and invasion assay of MGC-803 cells

**Figure 8.**
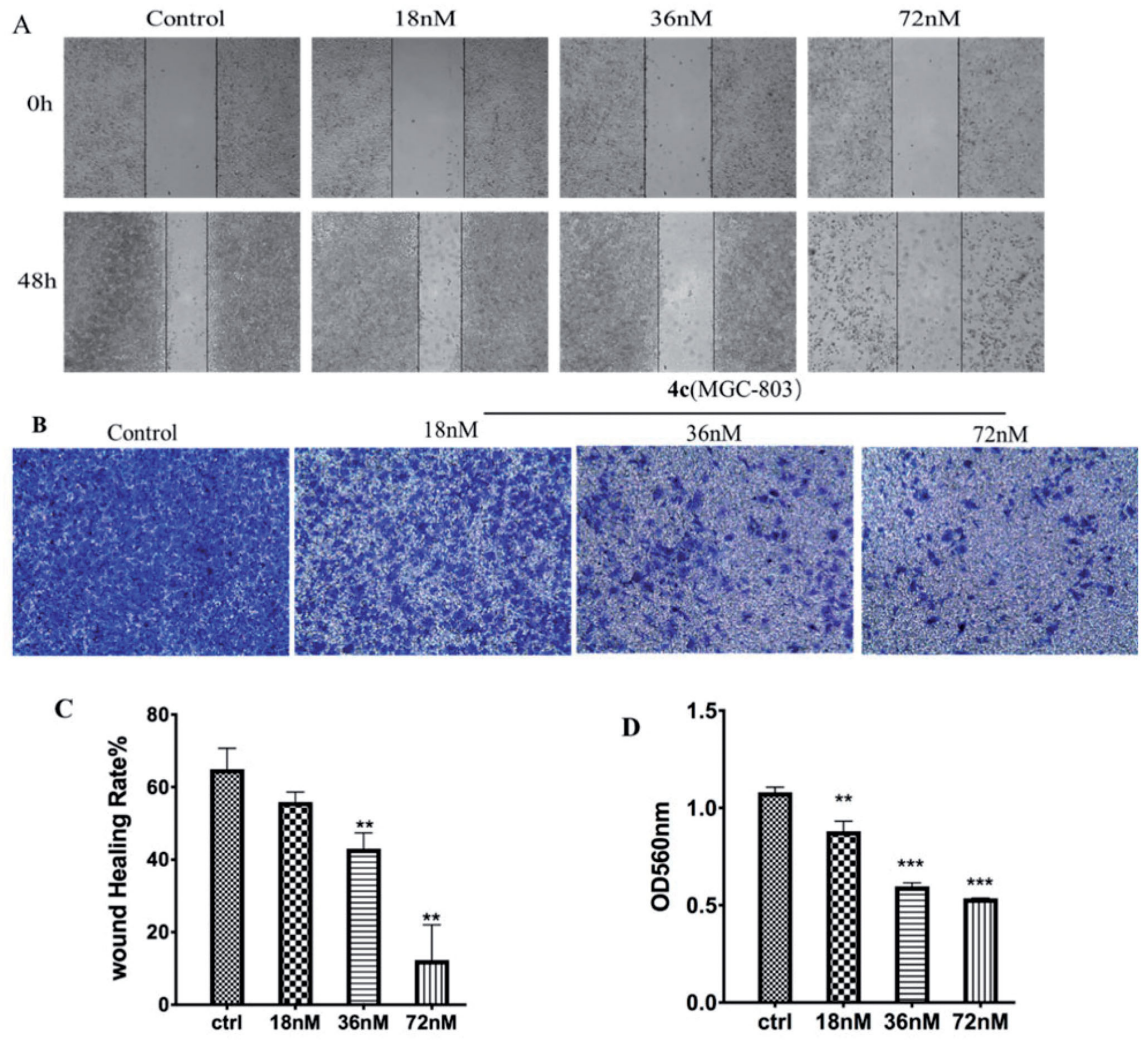
Effects of compound **4c** on the migration and invasion of MGC-803 cells. (A) Images of MGC-803 cells migration inhibited by **4c**; (B) Suppressing effects of **4c** on the invasion of MGC-803 cells; (C) Histograms display the percentage of healing cells; (D) Histograms display the percentage of invasion cells.

Metastasis is one of the leading causes of morbidity and mortality of cancer patients. Microtubules-targeting inhibitors have also been reported to interfere with cell migration and invasion. Accordingly, the *in vitro* would healing assay and Transwell assays were performed to investigate the inhibitory effect of compound **4c** on the migration and invasion of MGC-803 cells. As shown Figure 8(A,C), the scratches healing rate in the control group was approximately 64%. By contrast, the cells treated with **4c** have much smaller scratched areas recovery at all concentrations. In particular, the migration rates were only 10% at 72 nM of **4c** treatment after 48 h, which indicated that **4c** could significantly impaired the migration of MGC-803 cells, and it was positively correlated with the concentration of **4c**. Cell invasion is an important malignant behaviour during cancer metastasis. In Tanswell assay, MGC-803 cells were seeded into upper chambers with pre-coated Matrigel stimulating base membrane in order to assess the cellular invasion. The data shown in Figure 8(B,D) demonstrated that after **4c** treatment, MGC-803 cells exhibited significant reduced invasion ability, mainly characterised by the decrease in absorbance value of crystal violet and the amount of MGC-3 cell invasion from the upper layer to lower layer compared with control group. These results suggested that compound **4c** could inhibit tumour cell migration and invasion in a dose-dependent manner *in vitro*.

### 2.3. Anti-angiogenesis activity

Quite a few classic tubulin polymerisation inhibitor, e.g. Taxol, 2-methoxyestradiol^21^, or CA-4^22^, show anti-angiogenic activity *in vitro* and *in vivo*. Due to its disorganised structure and permeability, the tumour vascular system has long been regarded as a unique target for chemotherapeutic agents that can inhibit the formation of new tumour vessels (anti-angiogenic effect) or destroy existing vessels (vascular disruption effect). For this reason, we further evaluated compound **4c** for inhibitory effects on the formation of vessel-like structure on Matrigel, as well as on the angiogenesis *in vitro* and *in vivo*.

#### 2.3.1. Migration and invasion assay of HUVEC

**Figure 9.**
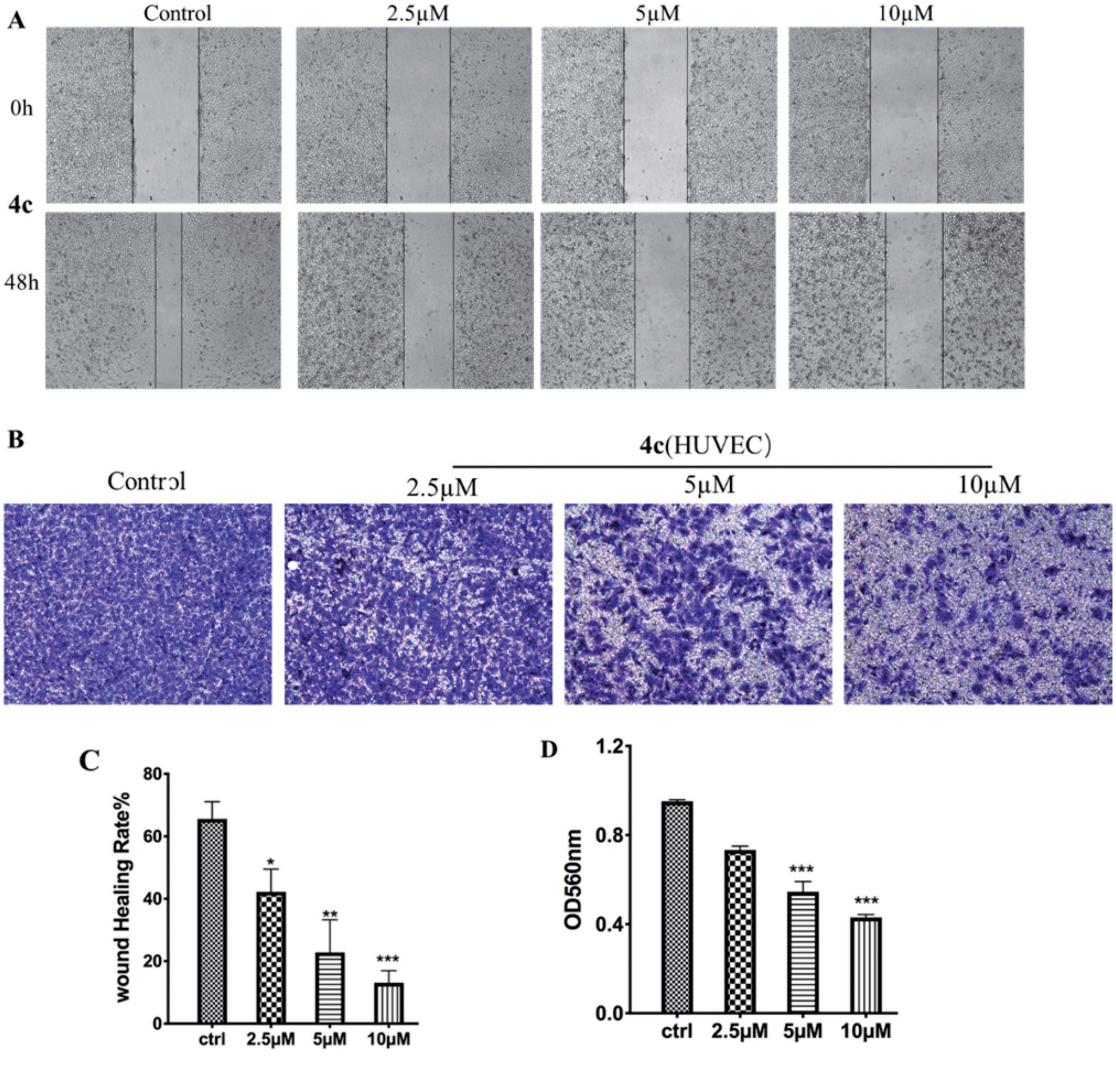
Effects of compound **4c** on the migration and invasion of HUVEC. (A) Images of HUVEC migration inhibited by **4c**; (B) Suppressing effects of **4c** on the invasion of HUVEC; (C) Histograms display the percentage of healing cells; (D) Histograms display the percentage of invasion cells.

HUVECs (human umbilical vein endothelial cells) grown on Matrigel were commonly used as a tube formation model for studying the physiological and pathological processes of the vascular system *in vitro*, and a widely studied human endothelial cell type in angiogenesis. The effect of **4c** on endothelial cell migration was assessed by using HUVECs. Standardised wounds were made in confluent monolayers of HUVECs and then exposed to compound **4c** at 2.5, 5 and 10 µM for 48 h. As shown in Figure 9(A,C), the wound closure rates after 48 h of **4c** treatment was only 11% at the dose of 10 µM, which is much slower than 65% of the control group. We further evaluated the capacity of **4 s** on endothelial cell invasion using a Transwell assay. As shown in Figure 9(B,D), the control HUVECs that was not treated displayed a complete invasion from the upper chamber to lower one. However, after exposed to **4c** at 2.5, 5 and 10 µM for 24 h, the number of cells invaded into lower chamber was gradually decreased in a dose-dependent manner.

#### 2.3.2. Tube formation of HUVECs

**Figure 10.**
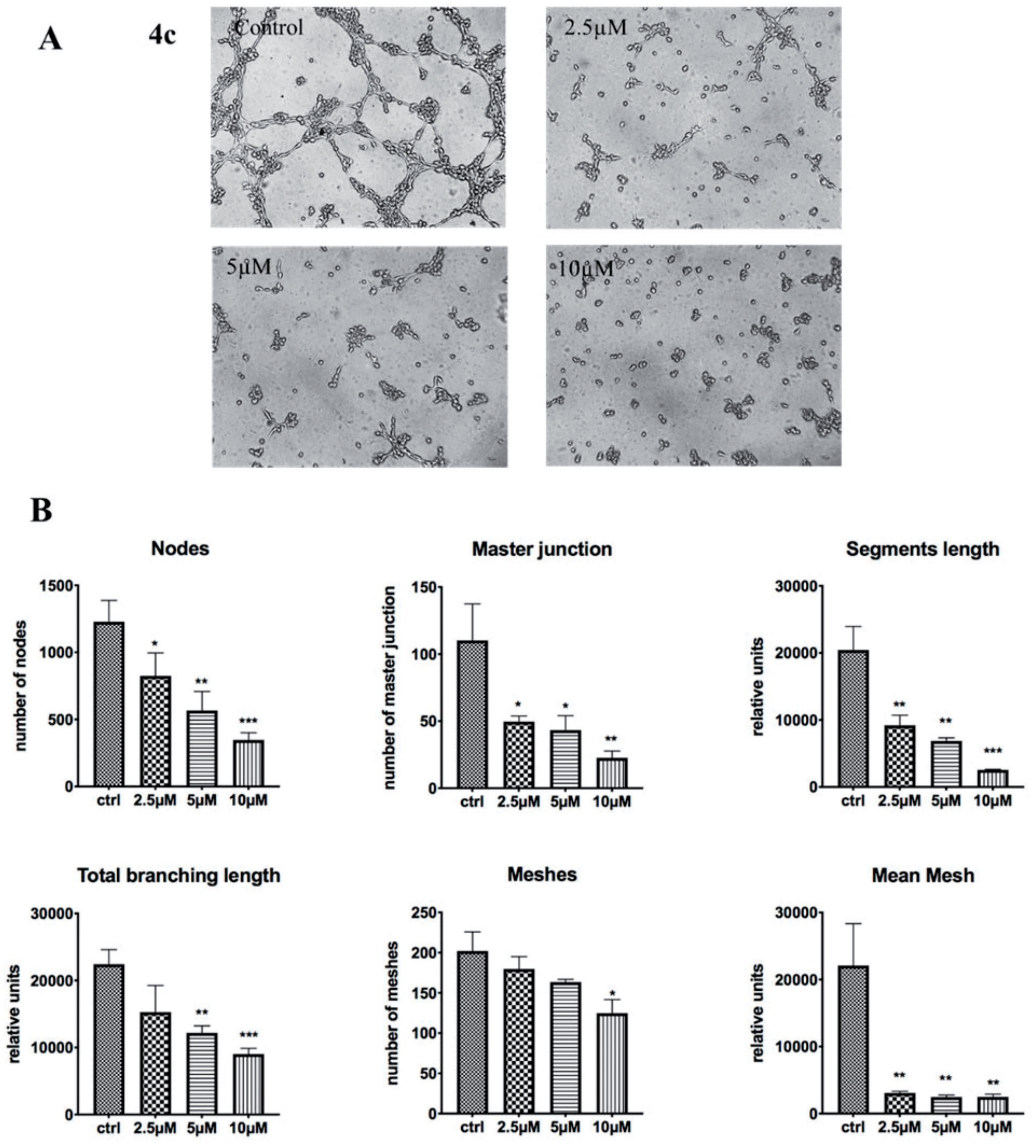
Effects of compounds **4c** on the tube formation of HUVECs *in vitro*; (A) Images for the tube-forming inhibitory activities for **4c** (0, 2.5, 5, 10 µM) in HUVECs; (C) Statistical analysis for the tube-forming capability. *p < 0.05, **p < 0.01,vs. control; ***p < 0.001 vs. control.

The formation of HUVEC tube represents the key steps in angiogenesis. Thus, we further tested the anti-vascular activity of **4c** by performing a tube formation assay. As shown in Figure 10 HUVECs in the control group formed integrated tubules with multicentric junctions on the Matrigel matrix, while the tubes were fragmentary after exposure to **4c**. HUVEC cord formation, tube area and length were dramatically suppressed even at relatively lower dose of 2.5 µM, with almost no tube structure observed. Statistical analysis showed that, compared to the control group, **4c** severely impaired the tube formation ability of HUVEC. Collectively, these results highlighted that compound **4c** has potent anti-vascular activity *in vitro*, which is evidenced by the disruption of tubule-like HUVECs, interference with HUVECs cell migration and invasion.

#### 2.3.3. Anti-angiogenesis in zebrafish embryos

**Figure 11.**
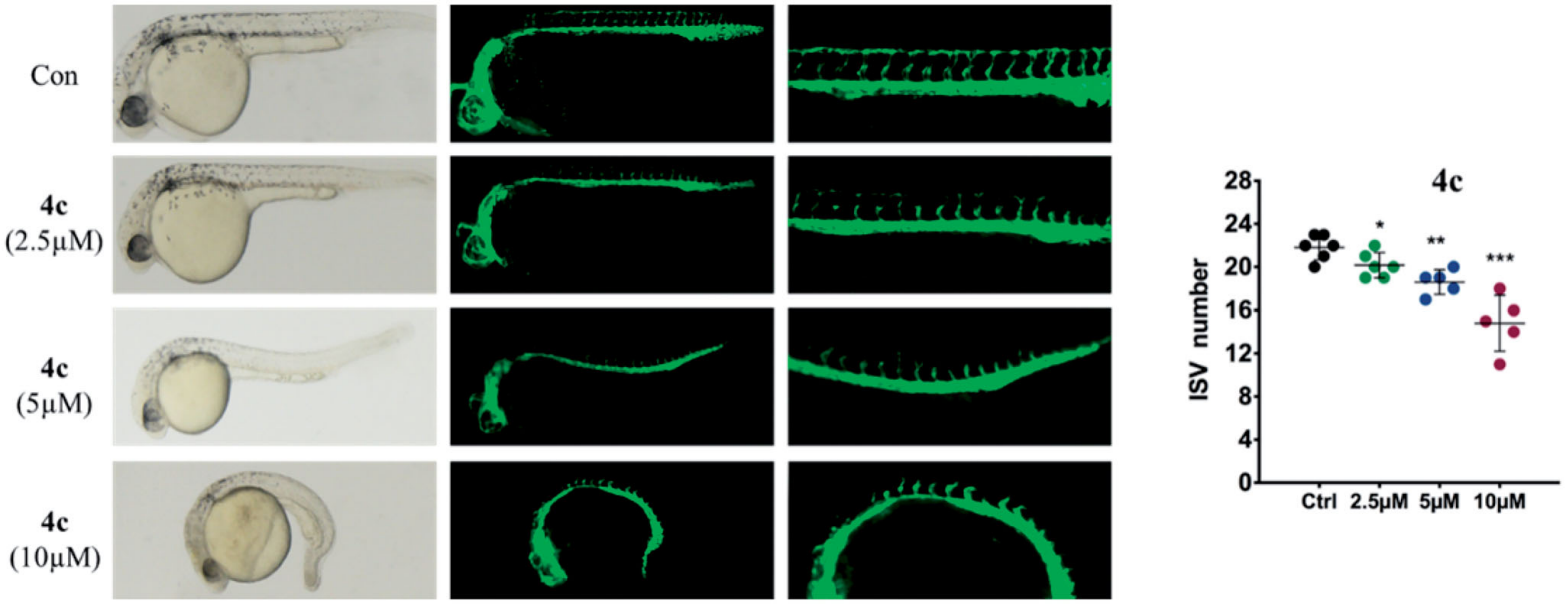
Anti-angiogenic effect of compounds **4c** in zebrafish embryos assay. Zebrafish embryos were incubated with **4c** at 0.5, 1, and 2 µM for 24 h.

Since zebrafish embryos emerged as a powerful model for studying human diseases, the transgenic zebrafish with green fluorescent protein (GFP) labelled endothelial cells have been used extensively for testing angiogenesis *in vivo*. In this work, an anti-angiogenetic activity of **4c** was confirmed by *Tg*(flk1: EGFP) zebrafish embryos *in vivo* assay. Fluorescently labelled 3hpf period embryos were treated with different doses of **4c** and incubated at 28.5 °C. Until 30hpf, zebrafish was imaged under a fluorescent microscopy to observe the affection of applied agents on intersegmental vessels (ISVs) development. As shown in the Figure 11, ISVs were clearly observed in newly born zebrafish embryos of solvent-treated control group, which were growing at regular longitudinal and equidistant intervals. Upon treatment with **4c**, the number and length of ISVs were all dose-dependently reduced. The embryos exhibited the anti-angiogenic phenotype already at 2.5 µM dose. At the dose ≥ 5 µM, embryos exhibited marked delay in development and only scattered or fragmentary blood vessels were observed, indicating that **4c** could effectively blocked the growth of normal angiogenesis.

## 3. Conclusions

In conclusion, a series of α-fluoro chalcone derivatives was designed, synthesised, and evaluated for their antiproliferative activity, where **4c** exhibited significant inhibition with IC_50_ ranging from 0.025 µM to 0.202 µM against five of the tested cell lines. Compound **4c**, which has a bromide substituted on indole moiety ring-B, remarkably suppressed tubulin polymerisation and disrupted microtubule networks of MGC-803 cells. Molecular docking results show that **4c** can bind to colchicine sites. Moreover, **4c** induced MGC-803 cell cycle arrest at the G2/M phase by regulation of G2/M-related protein expression of p-Cdc2, Cyclin B1 and p21. Besides, **4c** induced MGC-803 cell apoptosis, which might be the resulting effects of the cleavage of apoptotic related protein, including Caspase-3/-7/-9 and cleaved PARP. Additionally, **4c** depolarised the mitochondria membrane potentials and induced ROS generation in MGC-803 cells.

The evaluation of capillary-like tube formation *in vitro* indicated that **4c** has an inhibitory potency on HUVECs cells proliferation, which was consistent with its anti-angiogenesis activity of zebrafish embryos *in vivo*. Moreover, compound **4c** dose-dependently inhibited the migration and invasion of HUVECs *in vitro*. Finally, the anti-metastasis and anti-invasion potential of **4c** were confirmed in MGC-803 cells by wound healing and Transwell assay on Matrigel. Therefore, **4c** deserved further investigation as a novel, structurally simple and efficacious tubule polymerisation inhibitor, and anti-angiogenesis agents for the treatment of cancer.

## 4. Experimental

### 4.1. Chemistry

All chemicals were obtained from commercial sources without further purification unless otherwise stated. Solvents and reagents were dried and purified through routine protocols. Column chromatography (CC) was performed on silica gel (200–300 mesh, Qingdao Ocean Chemical Company, China). Thin-layer chromatography (TLC) was used to monitor reactions on GF254 silica gel glass plates (Qingdao Ocean Chemical Co., China). ^1^HNMR and ^13^CNMR were recorded on a Bruker AV-400 (400 MHz) NMR instrument at 400 and 100 MHz for deuterated chloroform (CDCl3) or deuterated dimethylsulphoxide (DMSO-d6) solutions, respectively. Chemical shifts were reported in parts per million (ppm) with tetramethylsilane as an internal standard. High resolution mass spectra (HRMS) were recorded with a Q-TOF micron spectrometer.

#### 4.1.1. 2-Bromo-1–(3,4,5-trimethoxyphenyl)ethan-1-one(2)

To a solution of 3,4,5-trimethoxyacetophenone (1.68 g, 8 mmol) in 100 ml DCM and 40 ml MeOH, was added TBABr_3_ and the reaction mixture was stirred at r.t. for 8 h. The solvent was removed under reduced pressure, and the residue was purified by column chromatography (PET: EA = 5:1) to obtain compound **4** as a white solid (2.0 g, 87%) . ^1^H NMR (400 MHz, CDCl_3_) *δ* 7.25 (s, 2H), 4.41 (s, 2H), 3.94 (s, 3H), 3.93 (s, 6H).

#### 4.1.2. 2-Fluoro-1–(3,4,5-trimethoxyphenyl)ethan-1-one (3)

Potassium fluoride (4.8 g, 84 mmol) was dried in an infra-red oven for 8 h in advance. Compound **2** (2.0 g, 7 mmol) and 18-Crown-6 (453 mg, 1.26 mmol) were dissolved in anhydrous acetonitrile and added dropwise through a dropping funnel to a flask containing potassium fluoride, and the reaction mixture was stirred at reflux for 16 h. The solvent was removed and the residue was extracted with DCM, dried over MgSO_4_, concentrated, and purified by column chromatography (PET: EA = 6:1) to give compound **3** as a light yellow solid(1.3g, 85%). ^1^H NMR (400 MHz, CDCl_3_) *δ* 7.16 (s, 2H), 5.55 (s, 1H), 5.43 (s, 1H), 3.94 (s, 3H), 3.92 (s, 6H).

#### 4.1.3. General procedure for synthesis of compound 4a∼4f

The intermediate **3** (50 mg, 0.22 mmol) and corresponding *1H*-indole-3-carbaldehyde(1.25 equivalent) was dissolved in anhydrous ethanol, a small amount of piperidine and glacial acetic acid was added, and then the reaction mixture wasstirred at 65 °C for 4 h. The crude mixture was allowed to cooled down to 0 °C and stirred further for 30 min, then filtrated through Celite, washed with anhydrous ethanol, to obtain the target product **4a∼4f.**

##### 4.1.3.1. *Methyl(Z)-3–(2-fluoro-3-oxo-3–(3,4,5-trimethoxyphenyl)prop-1-en-1-yl)-1H-indole-6-carboxylate (4a*)

M. p. 207.3–209.1 °C; ^1^H NMR (400 MHz, CDCl_3_) *δ* 9.33 (s, 1H), 8.26 − 8.19 (m, 1H), 8.09 (s, 1H), 7.93 (dd, *J* = 8.4, 1.3 Hz, 1H), 7.76 (d, *J* = 8.4 Hz, 1H), 7.35 (d, *J* = 37.5 Hz, 1H), 7.21 (s, 2H), 3.97 (s, 3H), 3.96 (s, 3H), 3.94 (s, 6H); ^13 ^C NMR (101 MHz, DMSO-d_6_) *δ* 185.30(d, *J* = 30.3 Hz), 166.78(s), 153.11(d, *J* = 258.6 Hz), 152.62(s), 139.94 (d, *J* = 12.1 Hz),141.18(s), 135.51(s), 133.94(d, *J* = 3.0 Hz), 133.82(s), 131.79(s), 129.74(s), 123.57(s), 121.14(s), 119.03(s), 115.00(d, *J* = 10.1 Hz), 114.03(s), 107.25(s), 106.59(s), 60.15(s), 56.06(s), 51.92(s); HRMS(ESI-TOF) *m/z* [M + H]^+^ calcd for C_22_H_20_FNO_6_, 414.1353, found 414.1339.

##### 4.1.3.2. (Z)-2-fluoro-3–(2-phenyl-1H-indol-3-yl)-1–(3,4,5-trimethoxyphenyl)prop-2-en-1-one (4 b)

M. p.150.7–152.4 °C; ^1^H NMR (400 MHz, CDCl_3_) *δ* 8.79 (d, *J* = 14.1 Hz, 1H), 8.10 (d, *J* = 7.3 Hz, 1H), 7.55 − 7.52 (m, 2H), 7.49 − 7.42 (m, 4H), 7.33 − 7.26 (m, 2H), 7.305 (d, *J =* 40.2 Hz, 1H) 7.16 (s, 2H), 3.92 (s, 3H), 3.85 (s, 6H); ^13 ^C NMR (101 MHz, CDCl_3_) *δ* 186.79(s), 186.52(d, *J* = 28.3 Hz), 153.58(d, *J* = 265.6 Hz), 152.83(s),141.94(s), 136.34(s), 132.03(s), 131.39(s), 129.23(s), 129.08(s), 128.93(s), 126.46(s), 123.68(s), 122.65, 122.48(s), 121.62 (d, *J* = 4.0 Hz), 115.56(d, *J* = 10.1 Hz), 111.22(s), 106.96(d, *J* = 5.1 Hz),106.66(d, *J* = 2.0 Hz), 60.97(s), 56.27(s); HRMS(ESI-TOF) *m/z* [M + H]^+^ calcd for C_26_H_22_FNO_4_, 432.1611, found 432.1620.

##### 4.1.3.3. (Z)-3–(6-bromo-1H-indol-3-yl)-2-fluoro-1–(3,4,5-trimethoxyphenyl)prop-2-en-1-one (4c)

M. p. 229.0–229.6 °C; ^1^H NMR (400 MHz, CDCl_3_) *δ* 8.73 (s, 1H), 7.90 (d, *J* = 2.2 Hz, 1H), 7.64 − 7.57 (m, 2H), 7.35 (dd, *J* = 8.6, 1.6 Hz, 1H), 7.20 (d, *J* = 0.8 Hz, 2H), 3.96 (s, 3H), 3.93 (s, 6H); ^13 ^C NMR (101 MHz, DMSO-d_6_) *δ* 185.27(d, *J* = 25.3 Hz), 153.02(d, *J* = 258.6 Hz), 152.61(s), 141.14(s), 137.04(s), 131.83(s), 131.67(s), 131.54(s), 125.23(s), 123.49(s), 120.97(d, *J* = 3.0 Hz), 115.19(s), 115.07(s), 114.769 s), 107.15(d, *J* = 1.0 Hz), 106.59(d, *J* = 2.0 Hz), 60.15(s), 56.05(s); HRMS(ESI-TOF) *m/z* [M + H]^+^ calcd for C_20_H_17_BrFNO_4_, 434.0398, found 434.0397.

##### 4.1.3.4. Methyl(Z)-3–(2-fluoro-3-oxo-3–(3,4,5-trimethoxyphenyl)prop-1-en-1-yl)-1H-indole-5-carboxylate (4d)

M. p. 80.4–81.8 °C; ^1^H NMR (400 MHz, CDCl_3_) *δ* 9.16 (s, 1H), 8.50 (d, *J* = 4.0 Hz, 1H), 8.03 − 7.96 (m, 2H), 7.48 (d, *J* = 8.6 Hz, 1H), 7.38 (d, *J* = 37.3 Hz, 1H), 7.21 (s, 2H), 3.98 (s, 3H), 3.96 (s, 3H), 3.94 (s, 6H); ^13 ^C NMR (101 MHz, DMSO-d_6_) *δ* 185.38(d, *J* = 25.3 Hz), 166.92(s), 152.98(d, *J* = 258.6 Hz), 152.61(s), 141.21(s),138.89(s), 132.51(d, *J* = 11.1 Hz), 131.84(s), 125.69(s), 123.36(s), 122.04(s), 121.65(d, *J* = 4.0 Hz), 115.16(d, *J* = 11.1 Hz), 112.30(s), 108.17(s), 106.72(d, *J* = 3.0 Hz), 60.15(s), 56.05(s), 51.82(s); HRMS (ESI-TOF) *m/z* [M + H]^+^ calcd for C_22_H_20_FNO_6_, 414.1339, found 414.1350.

##### 4.1.3.5. (Z)-2-fluoro-3–(2-methyl-1H-indol-3-yl)-1–(3,4,5-trimethoxyphenyl)prop-2-en-1-one (4e)

M. p. 204.0–206.4 °C;^1^H NMR (400 MHz, CDCl_3_) *δ* 8.51 (s, 1H), 7.95 (d, *J* = 8.0 Hz, 1H), 7.34 − 7.30 (m, 1H), 7.25 − 7.19 (m, 4H), 7.23 (d, *J* = 40.4 Hz,1H), 3.95 (s, 3H), 3.93 (s, 6H), 2.54 (s, 3H); ^13 ^C NMR (101 MHz, Chloroform-d) *δ* 186.72(d, *J* = 27.3 Hz), 152.88(s), 152.57(d, *J* = 261.6 Hz),141.87(s), 139.88(s), 135.76(s), 126.42(s), 122.68(s), 121.28(d, *J* = 4.0 Hz), 121.13(s), 115.05(d, *J* = 10.1 Hz), 110.78(s), 106.95(d, *J* = 4.0 Hz), 106.42(d, *J* = 1.0 Hz), 61.01(d, *J* = 2.0 Hz), 56.33(d, *J* = 2.0 Hz), 12.95; HRMS (ESI-TOF) *m/z* [M + H]^+^ calcd for C_21_H_20_FNO_4_, 370.1455, found 370.1444.

##### 4.1.3.6. Methyl(Z)-3–(2-fluoro-3-oxo-3–(3,4,5-trimethoxyphenyl)prop-1-en-1-yl)-1H-indole-4-carboxylate (4f)

M. p. 118.0–119.0 °C; ^1^H NMR (400 MHz, CDCl_3_) *δ* 9.45 (s, 1H), 8.23 (d, *J* = 2.9 Hz, 1H), 8.12 (d, *J* = 39.9 Hz, 1H), 7.80 (dd, *J* = 7.5, 0.9 Hz, 1H), 7.65 (dd, *J* = 8.1, 0.9 Hz, 1H), 7.29 (d, *J* = 7.8 Hz, 1H), 7.18 (s, 2H), 3.98 (s, 6H), 3.94 (s, 3H), 3.80 (s, 3H); ^13 ^C NMR (101 MHz, DMSO-d_6_) *δ* 185.77(d, *J* = 23.23 Hz), 167.81(s), 152.51(d, *J* = 26.26 Hz), 140.66(d, *J* = 74.74 Hz), 137.30(s), 124.15(s), 123.59(s), 122.97(s), 121.68(s), 118.83 (d, *J* = 6.06 Hz), 106.34(s), 55.89(d, *J* = 2.02 Hz), 55.47(s), 51.66(s); HRMS (ESI-TOF) *m/z* [M + H]^+^ calcd for C_22_H_20_FNO_6_, 414.1353, found 414.1338.

#### 4.1.4. General procedure for synthesis of compound 4 g∼4j,4s,4t

38 mg of sodium (1.65 mmol) was dispersed into anhydrous methanol under argon atmosphere. After the disappearance of sodium, the intermediate **3** (50 mg, 0.22 mmol) and corresponding aromatic aldehyde (1.25 equivalents) was added and stirred at room temperature for 10 min and then stirred at 65 °C for additional 5 h. After completion of the reaction, the mixture was precipitated after 30 min of stirring under ice bath, filtered, and washed with cold methanol, to afford the target compounds.

##### 4.1.4.1. (Z)-3-(benzo[b]thiophen-3-yl)-2-fluoro-1–(3,4,5-trimethoxyphenyl)prop-2-en-1-one (4 g)

M. p. 118.0–119.0 °C; ^1^H NMR (400 MHz, CDCl_3_) *δ* 8.24 (s, 1H), 7.90 (dd, *J* = 19.6, 7.5 Hz, 2H), 7.46 (dtd, *J* = 17.8, 7.2, 1.1 Hz, 2H), 7.33 (d, *J* = 35.7 Hz, 1H), 7.25 (d, *J* = 1.0 Hz, 2H), 3.97 (s, 3H), 3.94 (s, 6H); ^13 ^C NMR (101 MHz, DMSO-d_6_) *δ* 152.70(d, *J* = 28.28 Hz), 142.18(d, *J* = 35.35 Hz), 138.96(d, *J* = 33.33 Hz), 137.57(s), 132.14(s), 130.93(s), 125.17(d, *J* = 12.12 Hz), 124.84(s), 124.57(s), 123.02(d, *J* = 12.12 Hz), 122.90(s), 122.12 (s), 121.70(s), 111.66(s), 106.97(d, *J* = 50.5 Hz), 59.99(d, *J* = 23.23 Hz), 56.13(s), 55.65(s) ; HRMS (ESI-TOF) *m/z* [M + H]^+^ calcd for C_20_H_17_FO_4_S, 373.0910, found 373.0989.

##### 4.1.4.2. (Z)-3-(benzo[b]thiophen-2-yl)-2-fluoro-1–(3,4,5-trimethoxyphenyl)prop-2-en-1-one (4 h)

M. p. 122.2–123.6 °C;^1^H NMR (400 MHz, CDCl_3_) *δ* 7.85 (m, 2H), 7.64 (s, 1H), 7.40 (m, 2H), 7.32 (d, *J* = 35.0 Hz, 1H), 7.24 (d, *J* = 1.1 Hz, 2H), 3.96 (s, 3H), 3.94 (s, 6H); ^13 ^C NMR (101 MHz, CDCl_3_) *δ* 185.23(d, *J* = 27.3 Hz),155.53(d, *J* = 276.7 Hz), 153.02(s), 142.81(s), 142.11(s), 142.03(s), 138.81(s), 133.97(d, *J* = 5.1 Hz), 130.80(s), 129.17 (d, *J* = 5.1 Hz), 126.09(s), 124.89(s), 124.42(d, *J* = 9.1 Hz), 122.31(s), 114.42(d, *J* = 9.1 Hz), 107.15(d, *J* = 6.1 Hz), 61.04(d, *J* = 2.0 Hz), 56.39(d, *J* = 1.0 Hz); HRMS (ESI-TOF) *m/z* [M + H]^+^ calcd for C_20_H_17_FO_4_S, 373.0910, found 373.0968.

##### 4.1.4.3. (Z)-2-fluoro-3–(3-methylbenzo[b]thiophen-2-yl)-1–(3,4,5-trimethoxyphenyl)prop-2-en-1-one (4i)

M. p. 140.5–143.4 °C; ^1^H NMR (400 MHz, CDCl_3_) *δ* 7.89 − 7.82 (m, 1H), 7.78 (dd, *J* = 6.1, 2.9 Hz, 1H), 7.51 (d, *J* = 35.5 Hz, 1H), 7.45 − 7.40 (m, 2H), 7.27 − 7.24 (m, 2H), 3.96 (s, 3H), 3.94 (s, 6H), 2.55 (s, 3H); ^13 ^C NMR (101 MHz, CDCl_3_) *δ* 185.27(d, *J* = 27.3 Hz), 155.28(d, *J* = 275.7 Hz), 153.00(s), 142.75(s), 141.36(d, *J* = 10.1 Hz), 139.05(s), 136.60(d, *J* = 3.0 Hz), 130.97(d, *J* = 2.0 Hz), 128.65(d, *J* = 7.1 Hz), 126.39(s), 124.50(s), 122.81(d, *J* = 3.0 Hz), 122.33(s), 112.43(d, *J* = 9.1 Hz), 107.18(d, *J* = 6.1 Hz), 106.07(s), 61.02(s), 56.37(s), 12.55(s); HRMS (ESI-TOF) *m/z* [M + Na]^+^ calcd for C_21_H_19_FO_4_S, 387.1066, found 387.1054.

##### 4.1.4.4. (Z)-3-(1H-benzo[d]imidazol-2-yl)-2-fluoro-1–(3,4,5-trimethoxyphenyl)prop-2-en-1-one (4j)

M. p. 178.9–180.9 °C; ^1^H NMR (400 MHz, CDCl_3_) *δ* 10.38 (s, 1H), 7.74 (s, 1H), 7.62(s,1H),7.30 (d, *J* = 3.6 Hz, 2H), 7.20 (s, 2H), 6.62 (s, 1H), 3.96 (s, 3H), 3.92 (s, 6H); ^13 ^C NMR (101 MHz, CDCl_3_) *δ* 185.06(d, *J* = 27.27 Hz), 157.43(d, *J* = 274.72 Hz), 153.14(s), 144.24(d, *J* = 3.03 Hz), 143.23(s), 130.13(s), 128.83(s), 110.61(d, *J* = 6.06 Hz), 107.10(d, *J* = 4.04 Hz), 61.09(s), 56.40(s), 30.57(s), 13.74(s); HRMS (ESI-TOF) *m/z* [M + H]^+^ calcd for C_19_H_17_FN_2_O_4_, 357.1251, found 357.1270.

##### 4.1.4.5. (Z)-3-(dibenzo[b, d]thiophen-2-yl)-2-fluoro-1–(3,4,5-trimethoxyphenyl)prop-2-en-1-one (4 s)

M. p. 142.6–143.8 °C; ^1^H NMR (400 MHz, CDCl_3_) *δ* 8.50 (s, 1H), 8.21 (dd, J = 5.8, 3.1 Hz, 1H), 7.94 − 7.86 (m, 2H), 7.81 (dd, J = 8.4, 1.6 Hz, 1H), 7.53 − 7.47 (m, 2H), 7.24 (d, J = 1.1 Hz, 2H), 7.10 (d, J = 36.5 Hz, 1H), 3.97 (s, 3H), 3.95 (s, 6H); ^13 ^C NMR (101 MHz, CDCl_3_) *δ* 186.48(d, *J* = 29.3 Hz), 155.97(d, *J* = 273.7 Hz), 153.01(s), 142.59(s), 141.39(s), 139.72(s), 136.12(s), 135.05(s), 131.16(s), 127.79(d, J = 4.0 Hz), 127.34(s), 124.82 (s), 123.76(d, *J* = 9.1 Hz), 123.22(s), 122.96(s), 121.76(s), 119.96(d, *J* = 5.1 Hz), 107.13(d, *J* = 5.1 Hz), 61.05(s), 56.41(s); HRMS (ESI-TOF) *m/z* [M + H]^+^ calcd for C_24_H_19_FO_4_S, 423.1066, found 423.1050.

##### 4.1.4.6. (Z)-3-(benzofuran-3-yl)-2-fluoro-1–(3,4,5-trimethoxyphenyl)prop-2-en-1-one (4t)

M. p.103.7–104.8 °C;^1^H NMR (400 MHz, CDCl_3_) *δ* 8.23 (s, 1H), 7.74 (s, 1H), 7.57 (s, 1H), 7.41 − 7.34 (m, 2H), 7.24 (d, J = 1.2 Hz, 2H), 7.16 (d, J = 37.8 Hz, 1H), 3.97 (s, 3H), 3.94 (s, 6H). ^13 ^C NMR (101 MHz, Chloroform-d) *δ* 155.02(s), 153.03(s), 148.46(s), 147.77(d, *J* = 15.15 Hz), 125.76(s), 125.41(s), 123.63(s), 119.63(d, *J* = 3.03 Hz), 111.87(s), 109.48(d, *J* = 12.12 Hz), 107.35(s), 107.10(d, *J* = 5.05 Hz), 106.76(s), 61.05(s), 56.38(s); HRMS (ESI-TOF) *m/z* [M + H]^+^ calcd for C_20_H_17_FO_5_, 357.1138, found 357.1143.

#### 4.1.5. General procedure for synthesis of compound 4k∼4q

To a solution of corresponding aromatic aldehyde (1 equivalent) in 5 ml anhydrous methanol, was added NaOH (4 equivalents) and compound **3** (1 equivalent), and then stirred at room temperature for 5 min and at 65 °C for additional 2 h. Reaction completion was monitored by TLC. The reaction mixture was precipitated after cooling and filtered to obtain the target compounds **4k∼4q**.

##### 4.1.5.1. (Z)-3-(benzo[d][1,3]dioxol-4-yl)-2-fluoro-1–(3,4,5-trimethoxyphenyl)prop-2-en-1-one (4k)

M. p. 148.1–149.1 °C; ^1^H NMR (400 MHz, CDCl_3_) *δ* 7.45 (d, *J* = 8.0 Hz, 1H), 7.19 (s, 2H), 7.05 (d, *J* = 36.9 Hz, 1H), 6.93 − 6.82 (m, 2H), 6.02 (s, 2H), 3.95 (s, 3H), 3.92 (s, 6H); ^13 ^C NMR (101 MHz, CDCl_3_) δ 186.27(d, *J* = 29.3 Hz), 156.18(d, *J* = 275.7 Hz), 152.96(s), 147.43(s),146.92(s), 142.60(s), 130.96(s), 122.32(d, *J* = 13.1 Hz), 122.02(s), 113.86(s), 112.44(d, *J* = 7.1 Hz), 109.80(d, *J* = 2.0 Hz), 107.14(d, *J* = 5.1 Hz), 101.28(s), 61.01(d, *J* = 1.0 Hz), 56.34(d, *J* = 2.0 Hz); HRMS (ESI-TOF) *m/z* [M + H]^+^ calcd for C_19_H_17_FO_6_, 361.1087, found 361.1084.

##### 4.1.5.2. (Z)-3–(6-bromobenzo[d][1,3]dioxol-5-yl)-2-fluoro-1–(3,4,5-trimethoxyphenyl)prop-2-en-1-one (4l)

M. p.156.3–157.8 °C; ^1^H NMR (400 MHz, CDCl_3_) *δ* 7.53 (s, 1H), 7.26 (d, *J* = 36.0 Hz, 1H), 7.18 (s, 2H), 7.11 (s, 1H), 6.06 (s, 2H), 3.95 (s, 3H), 3.94 (s, 6H); ^13 ^C NMR (101 MHz, CDCl_3_) *δ* 186.49(d, *J* = 27.3 Hz), 155.06(d, *J* = 273.7 Hz), 152.98(s), 149.61(d, *J* = 2.0 Hz), 147.72(s), 142.55(s), 131.01(s), 124.38(d, *J* = 4.0 Hz), 118.79(d, *J* = 3.0 Hz), 117.93(d, *J* = 2.0 Hz), 113.18(s), 110.33(d, *J* = 15.2 Hz), 107.08(d, *J* = 4.0 Hz), 102.36(s), 61.01(d, *J* = 1.0 Hz), 56.32(d, *J* = 1.0 Hz).HRMS (ESI-TOF) *m/z* [M + H]^+^ calcd for C_19_H_16_BrFO_6_, 439.0193, found 439.0175.

##### 4.1.5.3. (Z)-3-(benzofuran-5-yl)-2-fluoro-1–(3,4,5-trimethoxyphenyl)prop-2-en-1-one (4 m)

M. p. 94.1–94.2 °C;^1^H NMR (400 MHz, CDCl_3_) *δ* 8.03 (s, 1H), 7.68 (d, *J* = 2.3 Hz, 1H), 7.55 (d, *J* = 8.8 Hz, 1H), 7.21 (d, *J* = 1.2 Hz, 2H), 7.02 (d, *J* = 36.6 Hz, 1H), 6.83 (s, 1H), 3.96 (s, 3H), 3.93 (s, 6H); ^13 ^C NMR (101 MHz, CDCl_3_) *δ* 186.65(d, *J* = 28.3 Hz), 155.34(d, *J* = 272.3 Hz),152.97(s), 146.11(s), 142.51(s), 131.25(s), 128.15(s), 127.30(d, *J* = 8.1 Hz), 124.04(d, *J* = 9.1 Hz), 120.40(d, *J* = 6.1 Hz), 111.96(s), 111.42(s), 107.11(d, *J* = 5.1 Hz), 106.85(s),65.57(s), 61.01(d, *J* = 1.0 Hz), 56.37(d, *J* = 1.0 Hz); HRMS (ESI-TOF) *m/z* [M + H]^+^ calcd for C_20_H_17_FO_5_, 357.1138, found 357.1142.

##### 4.1.5.4. (Z)-3–(2,3-dihydrobenzofuran-5-yl)-2-fluoro-1–(3,4,5-trimethoxyphenyl)prop-2-en-1-one (4n)

M. p. 100.8–103.7 °C;^1^ H NMR (400 MHz, CDCl_3_) *δ* 7.65 (s, 1H), 7.46 (d, *J* = 8.4 Hz, 1H), 7.17 (s, 2H), 6.88 (d, *J* = 28.6 Hz, 1H), 6.82 (s, 1H), 4.65 (t, *J* = 8.7 Hz, 2H), 3.94 (s, 3H), 3.92 (s, 6H), 3.27 (t, *J* = 8.6 Hz, 2H); ^13 ^C NMR (101 MHz, CDCl_3_) *δ* 186.40(d, *J* = 28.28 Hz), 161.94(s), 152.93(s), 142.26(s), 132.12(d, *J* = 7.07 Hz), 131.53(s), 128.19(s), 127.46(d, *J* = 10.1 Hz), 124.12(d, *J* = 5.05 Hz), 120.72(d, *J* = 12.12 Hz), 109.86(s), 106.99(d, *J* = 5.05 Hz), 71.93(s), 61.00(s), 56.34(s), 29.33(s); HRMS (ESI-TOF) *m/z* [M + H]^+^ calcd for C_20_H_19_FO_5_, 359.1289, found 381.1286.

##### 4.1.5.5. (Z)-3-(benzofuran-2-yl)-2-fluoro-1–(3,4,5-trimethoxyphenyl)prop-2-en-1-one (4o)

M. p. 115.5–116.6 °C; ^1^H NMR (400 MHz, CDCl_3_) *δ* 7.65 (d, *J* = 7.8 Hz, 1H), 7.52 (d, *J* = 8.2 Hz, 1H), 7.39 (d, *J* = 7.2 Hz, 1H), 7.32 (s, 1H), 7.28 (s, 1H), 7.23 (s, 2H), 7.08 (d, *J* = 34.4 Hz,1H), 3.96 (s, 3H), 3.94 (s, 6H); ^13 ^C NMR (101 MHz, CDCl_3_) *δ* 185.09(d, *J* = 27.3 Hz), 155.29(d, *J* = 203.0 Hz), 153.04(s), 148.75(s), 142.89(s), 130.63(s), 128.48(s), 126.37(s), 123.53(s), 121.85(s), 112.13(d, *J* = 12.1 Hz), 112.01(s), 111.48(s), 109.30(d, *J* = 9.1 Hz), 107.16(d, *J* = 5.1 Hz), 106.16(s), 61.04(d, *J* = 2.0 Hz), 56.39(d, *J* = 1.0 Hz); HRMS (ESI-TOF) *m/z* [M + H]^+^ calcd for C_20_H_17_FO_5_, 357.1138, found 357.1127.

##### 4.1.5.6. (Z)-2-fluoro-3–(3-methylbenzofuran-2-yl)-1–(3,4,5-trimethoxyphenyl)prop-2-en-1-one (4p)

M. p.117.4–118.8 °C; ^1^H NMR (400 MHz, CDCl_3_) *δ* 7.55 (dd, *J* = 12.7, 8.0 Hz, 2H), 7.39 (d, *J* = 7.2 Hz, 1H), 7.30 (d, *J* = 1.4 Hz, 2H), 7.28 (s, 1H), 7.09 (d, *J* = 34.5 Hz, 1H), 3.96 (s, 3H), 3.95 (s, 6H), 2.40 (d, *J* = 1.4 Hz, 3H); ^13 ^C NMR (101 MHz, CDCl_3_) *δ* 185.45(d, *J* = 27.3 Hz), 155.40(d, *J* = 3.0 Hz), 154.96(d, *J* = 281.8 Hz), 152.96(s), 145.72(d, *J* = 5.1 Hz), 142.79(s), 130.80(d, *J* = 2.0 Hz), 129.07(s), 126.78(s), 122.99(s), 122.01(d, *J* = 5.1 Hz), 111.52(s), 107.28(d, *J* = 6.1 Hz), 106.07(s), 105.76(d, *J* = 8.1 Hz), 61.01, 56.35, 8.83; HRMS (ESI-TOF) *m/z* [M + H]^+^ calcd for C_21_H_19_FO_5_, 371.1295, found 371.1284.

##### 4.1.5.7. (Z)-3-(dibenzo[b,d]furan-2-yl)-2-fluoro-1–(3,4,5-trimethoxyphenyl)prop-2-en-1-one (4q)

M. p. 114.0–116.4 °C; ^1^H NMR (400 MHz, CDCl_3_) *δ* 8.36 (s, 1H), 7.99 (s, 1H), 7.81 (d, *J* = 10.2 Hz, 1H), 7.61 (dd, *J* = 8.3, 6.5 Hz, 2H), 7.51 (d, *J* = 7.7 Hz, 1H), 7.39 (s, 1H), 7.24 (d, *J* = 0.9 Hz, 2H), 7.16 (d, *J* = 22.4 Hz, 1H), 3.97 (s, 3H), 3.95 (s, 6H); ^13^CNMR (101 MHz, CDCl_3_) *δ* 186.55(d, *J* = 28.3 Hz), 156.84(d, *J* = 3.0 Hz), 156.9(s), 155.52(d, *J* = 254.5 Hz), 142.53(s), 131.21(s), 130.17(d, *J* = 8.1 Hz), 127.87(s), 126.34(d, *J* = 8.1 Hz), 125.08(s), 123.63(s), 123.23(s), 123.17(d, *J* = 9.1 Hz), 120.92(s), 120.04(d, *J* = 6.1 Hz), 112.22(d, *J* = 32.3 Hz), 107.10(d, *J* = 5.1 Hz), 61.05(s), 56.39(s); HRMS (ESI-TOF) *m/z* [M + H]^+^ calcd for C_24_H_19_FO_5_, 407.1295, found 407.1281.

#### 4.1.6. General procedure for synthesis of compound 4r

Compound **3** (50 mg, 0.22 mmol), 2-nitro piperonal (45 mg, 0.23 mmol), NaOH (20 mg, 0.5 mmol), K_2_CO_3_ 350 mg (2.5 mmol) were added to the mortar, mixed with an appropriate amount of silica gel, stirred and then ground. Reaction completion was evaluated by silica TLC. The reaction was quenched with water, extracted with DCM, and the organic phase was dried over MgSO_4_, concentrated *in vacuo*, and then recrystallized with ethanol to obtain **4r** (42 mg).

##### 4.1.6.1. (Z)-2-fluoro-3–(6-nitrobenzo[d][1,3]dioxol-5-yl)-1–(3,4,5-trimethoxyphenyl)prop-2-en-1-one (4r)

M. p. 174.7–176.6 °C; ^1^H NMR (400 MHz, CDCl_3_) *δ* 7.59 (s, 1H), 7.47 (d, *J* = 29.9 Hz, 1H), 7.26 (s, 2H), 7.11 (s, 1H), 6.16 (d, *J* = 2.0 Hz, 2H),3.97 (s, 6H), 3.95 (s, 3H); ^13 ^C NMR (101 MHz, DMSO-d_6_) *δ* 192.93 (d, *J* = 20.2 Hz), 151.91(s), 147.19(s), 141.98(s), 140.70(s), 133.66(d, *J* = 6.06 Hz), 129.95(s), 124.48(s), 108.78(s), 105.89(s), 104.52(s), 103.50(s), 95.04(s), 93.18(s), 68.24(d, *J* = 19.19 Hz), 60.16 (d, *J* = 2.02 Hz), 56.05(d, *J* = 3.03 Hz)； HRMS (ESI-TOF) *m/z* [M + H]^+^ calcd for C_19_H_16_FNO_8_, 406.0938, found 406.0927.

### 4.2. Biology

#### 4.2.1. Materials

MCF-7, MGC-803, Hela and HepG-2 cell lines were cultured in a base medium (90% DMEM and 10% FBS), A549 and U937 were cultured in a base medium (90% PRMI-1640 and 10% FBS) at 37 °C, 5% CO_2_ and a saturated humidity atmosphere. All the mediums were supplemented with 10% FBS and 1% ampicillin/streptomycin (all cells were purchased from Shanghai Institutes for Biological Sciences, Chinese Academy of Sciences, Shanghai, China).

#### 4.2.2. Cell lines growth inhibition. SRB

A549, MGC-803, MCF-7, HepG2 and Hela cells were seeded at the appropriate cell line density (3 × 10^3^ to 4 × 10^3^ cells/well). The cells were then incubated at 37 °C for 24 h in a humidified 5%CO_2_ in 96-well plates prior to experiments. When the cells adhered, different concentrations of test compounds were added to each well as shown in the figure and table. After a further 72 h of incubation, cell proliferation was quantified with SRB (sulfonamide B) to determine the number of surviving cells. The absorbance at 560 nm was measured by microplate. The experiment was repeated three times, and the data were listed in the form of mean and standard deviation.

#### 4.2.3. CCK8 procedure

MGC-803 cells were collected and seeded into 96 well plates at a density of 8000/well. The mediums containing drugs and cell suspension were added into the well. The cells were incubated in 5% CO_2_ incubator at 37 °C for 72 h. Each well was added 10 µL CCK8 solution, and the incubation time was 3–4 h. The absorbance value at 450 nm was measured by microplate reader. Data are calculated with GraphPad Prism5 software.

#### 4.2.4. Immunofluorescent staining

MGC-803 cells (3 × 10^4^ cells/well) were seeded into 6 well-plates and then treated with vehicle control 0.1% DMSO and **4c** (18, 36, 72nΜ). The cells were rinsed twice with PBS and fixed by 4% paraformaldehyde for 20 min. Subsequently, microtubule detection was performed using a fluorescent antibody against α-tubulin in 5% BSA and incubated for 5 ∼ 6 h at room temperature. To stain cell nucleus, crystals were incubated with Hoechst for 10 min. After washing with PBST to remove redundant stain, the samples were visualised under an Olympus laser scanning confocal microscope.

#### 4.2.5. Cell cycle distribution assay

MGC-803 cells (15 × 10^4^ cells/well) were inoculated in 6-well plates and incubated at 37 °C for 24 h, and then treated with **4c** (18, 36, 72nΜ). The collected cells were washed with cold PBS and then fixed in 75% ethanol in PBS at −20 °C overnight. The cells were then washed twice with PBS buffer and then stained with 500 μL of sodium iodide (PI, 5 mg) staining solution containing isothiocyanate fluorophore for 15 min in the dark. Samples were immediately assayed for DNA content using a flow cytometer (Becton, Dickinson and Company, USA). The percentage of cell cycle phases was analysed by the software provided in the instrument.

#### 4.2.6. Cell apoptosis assay

MGC-803 cells at 8 × 10^4^ cells/well were seeded in 12-well cell culture plates overnight, and then the cultured cells were incubated with a dose range of compounds **4c** for 48 h. The treated or untreated cells were washed twice with PBS and stained by Annexin V-PE and PI for 15 min without light exposure according to the manufacturer’s protocol. Apoptosis was quantified using a flow cytometer (Becton, Dickinson and Company, USA).

#### 4.2.7. Western blot assay

The protein levels in MGC-803 cells were assessed via western blot analysis. Exponentially growing MGC-803 cells were seeded at a density of 6 × 10^5^/dishes and incubated overnight. Then 18, 36, 72nΜ of **4c** were added in triplicate for 48 h incubation. After the cells were washed with cold PBS, the supernatant was removed. The supernatant was collected by centrifuging at 12,000 g for 10 min at 4 °C. The protein concentration in the supernatant was determined by using a BSA protein assay kit. Samples were prepared in SDS-PAGE loading buffer after BSA analysis to quantify proteins, then boiled for 10 min at 100 °C. Western blot analyses were conducted after separation by SDS-PAGE electrophoresis and transfer to nitrocellulose filter (NC) membranes. Immunoblotting was performed according to the antibody manufacturers’ recommendations.

#### 4.2.8. Mitochondrial membrane potential analysis

The cultured cells were spread in 6-well plates with about 1.5 × 10^5^ cells per well and incubated overnight at 37 °C. Different concentrations of the **4c** were added to the wells, and the incubation was continued for 48 h. The wells were washed twice with JC-1 staining buffer (1×) after resuspension with an appropriate amount of JC-1 staining buffer (1×), flow cytometry assays were performed according to standard procedures

#### 4.2.9. Measurement of intracellular ROS generation

Load the probe and dilute DCFH-DA with serum-free culture medium at 1:1000 to a final concentration of 10 μM/mL. Spread the cultured cells in a 6-well plate with about 1.5 × 10^5^ cells per well and incubate overnight at 37 °C. After 48 h of incubation with **4c** (18, 36, 72nΜ) the cell culture medium was removed and diluted DCFH-DA was added and incubated for 20 min at 37 °C in an incubator, and the mixture was inverted every 3–5 min. To fully remove the DCFH-DA that did not enter the cells, the cells were washed three times with serum-free cell culture medium. Add 200 μL of PBS for resuspension, place on ice, and subsequently assay by flow cytometry using standard procedures.

#### 4.2.10. Tube formation assay

The matrix gel was thawed overnight at 4 °C and then added to 96 well plates. After incubated at 37 °C for 45 min, the matrix gel was solidified. Different concentrations of **4c** were added to each well at the density of HUVECs was 1 × 10^5^/well. After incubated at 37 °C for 6–8 h, the formation of tubules was observed under microscope

#### 4.2.11. Would healing migration assays

MGC-803 cells and HUVECs were seeded in 24-well plates and cultivated for 24 h at 37 °C. The cells were scribed vertically with a gun tip and washed with PBS to remove dead cells. The containing different concentrations of **4c** were added to the scratched monolayers. The migrated cells were photographed under a light microscope at indicated time points from the scratch. Take a picture and use the image J software to calculate the area of the cell scratches.

#### 4.2.12. Transwell cell invasion experiment

Matrix gel was thawed overnight at 4 °C. Transwell chambers were placed into 24-well plates with 6 × 10^4^ cells per well, different concentrations of **4c** prepared 10% FBS medium were added, incubated at 37 °C with 5% CO_2_ for 48 h. Then the cells were washed three times with PBS and fixed cells by 4% paraformaldehyde for 30 min. Subsequently, 0.2% crystal violet solution was added, stained for 30 min, and the invasion of cells was observed under the microscope. Transwell was placed into a 24-well plate, 35% acetic acid (300 μL) was added to the upper chamber of the chamber, and after acetic acid completely entered the lower chamber, 100 μL of acetic acid from the lower chamber was aspirated into a 96-well plate, and three replicate wells of each concentration were shaken for 10 min, and the absorbance was measured at 560 nm with an enzyme marker.

#### 4.2.13. Zebrafish angiogenesis assay

The transgenic flk: enhanced GFP zebrafish embryos were transferred into 6-well plates (*n* = 30 per well) with 1 ml of aquaculture water (0.2 g/L of Instant Ocean Salt in distilled water) and raised at 28 °C. Then the embryos dechorionated at 12 h post fertilisation (hpf) was treated with the indicated concentrations of **4c** or vehicle (DMSO). About 30 embryos were screened for each concentration gradient. At 30hpf, the zebrafish embryos were stripped, anaesthetised, and fixed with agarose gel. The images were taken under the microscope, and the number of blood vessels between the segments was recorded. Results were obtained from three independent determinations and presented as mean ± SD.

## Supplementary Material

Supplemental Material
