# Supplementary material for: Identification of novel non-toxic and anti-angiogenic α-fluorinated chalcones as potent colchicine binding site inhibitors
Source: J Enzyme Inhib Med Chem. 2022 Jan 3;37(1):339–54. doi: 10.1080/14756366.2021.2014831 (PMC8741257; doi:10.1080/14756366.2021.2014831)
Supplement: Supplemental Material [file IENZ_A_2014831_SM9286.pdf]

Identification of novel non-toxic and anti-angiogenic α-fluorination chalcones as potent colchicine binding site inhibitors

Authors: Moran Sun[b], Minghua Yuan[b], Yingying Kang[b], Jinling Qin[b], Yixin Zhang[b], Yongtao Duan[a], Longfei Wang*[a], Yongfang Yao*[a,b]

Affiliation:

a. Henan provincial key laboratory of children's genetics and metabolic diseases, Children's Hospital Affiliated to Zhengzhou University, Zhengzhou University, Zhengzhou 450018, China

b. School of Pharmaceutical Sciences, and Institute of Drug Discovery & Development, Zhengzhou University, 100 Kexue Avenue, Zhengzhou, Henan 450001, China.

*Corresponding authors: yongfangyao@zzu.edu.cn (Yongfang Yao), dragonfly_wang@163.com (Longfei Wang)

Supplementary Material

Contents:

1. ^{1}H、^{13}C NMR and HRMS spectra of synthetized products 2

2. Cell arrest western blot assays films of compound 4c(48h) 33

3. Cell apoptosis western blot assays films of compound 4c 33

1. ^{1}H、^{13}C NMR and HRMS spectra of synthetized products

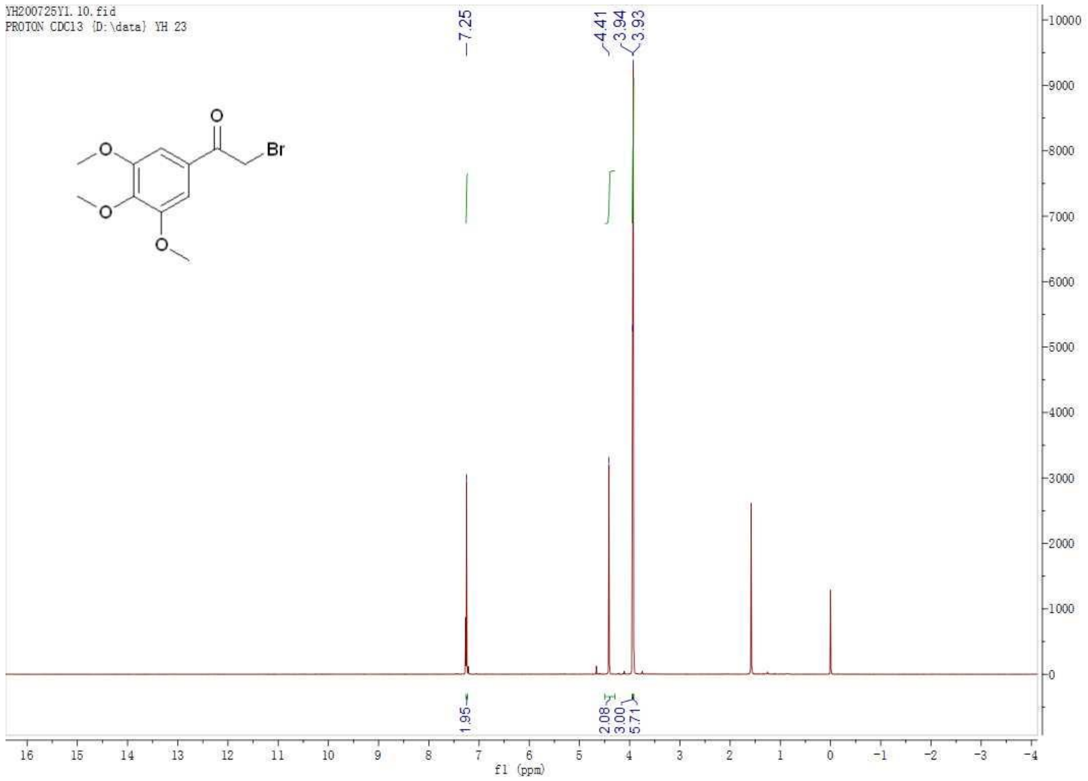

^{1}H NMR of **2**

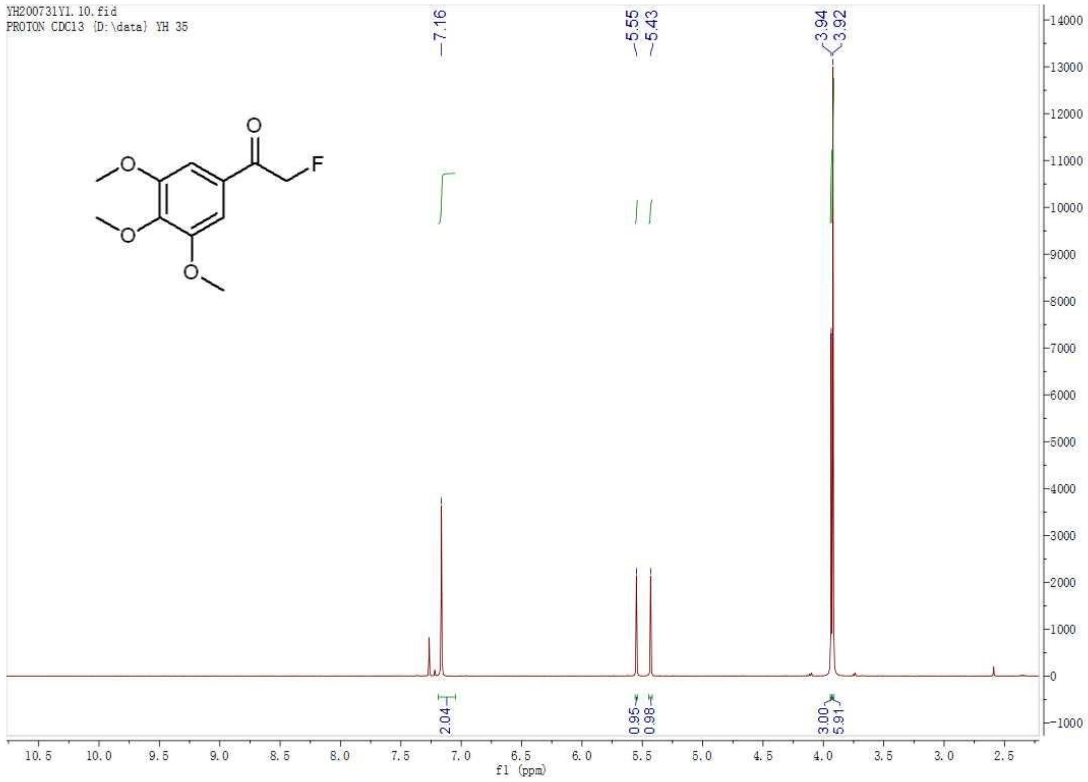

^{1}H NMR of **3**

(1) ^{1}H、^{13}C NMR and HRMS spectra of **4a**

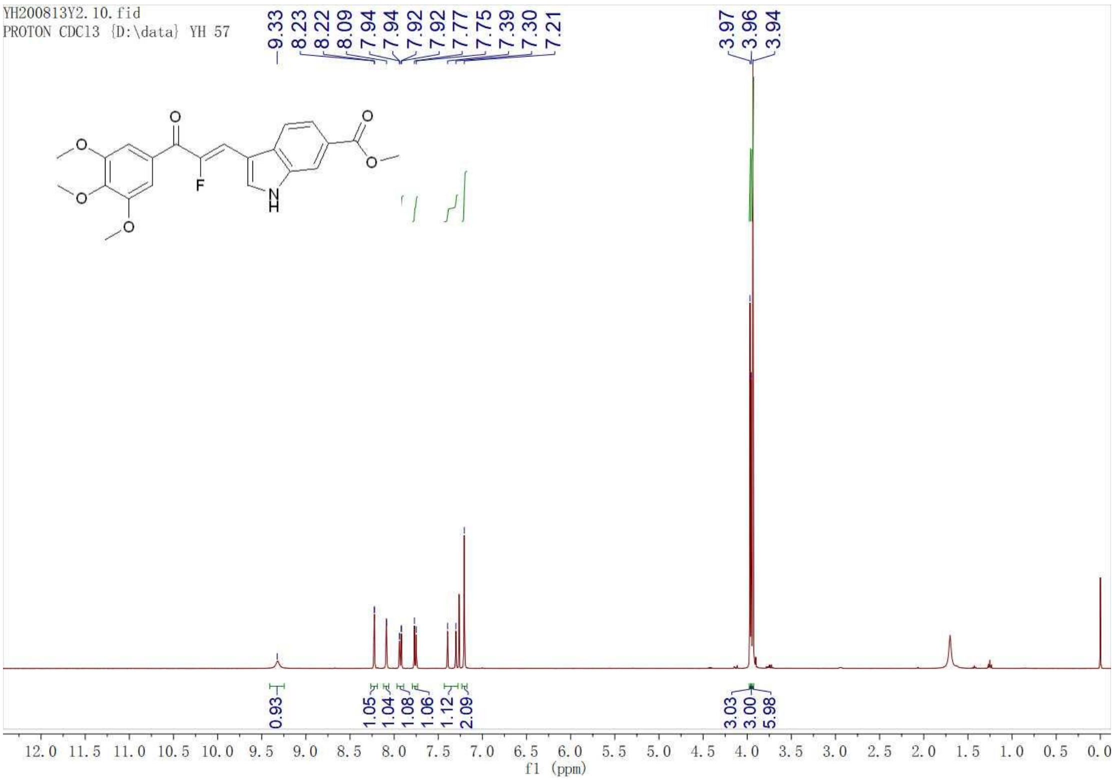

^{1}H NMR of **4a**

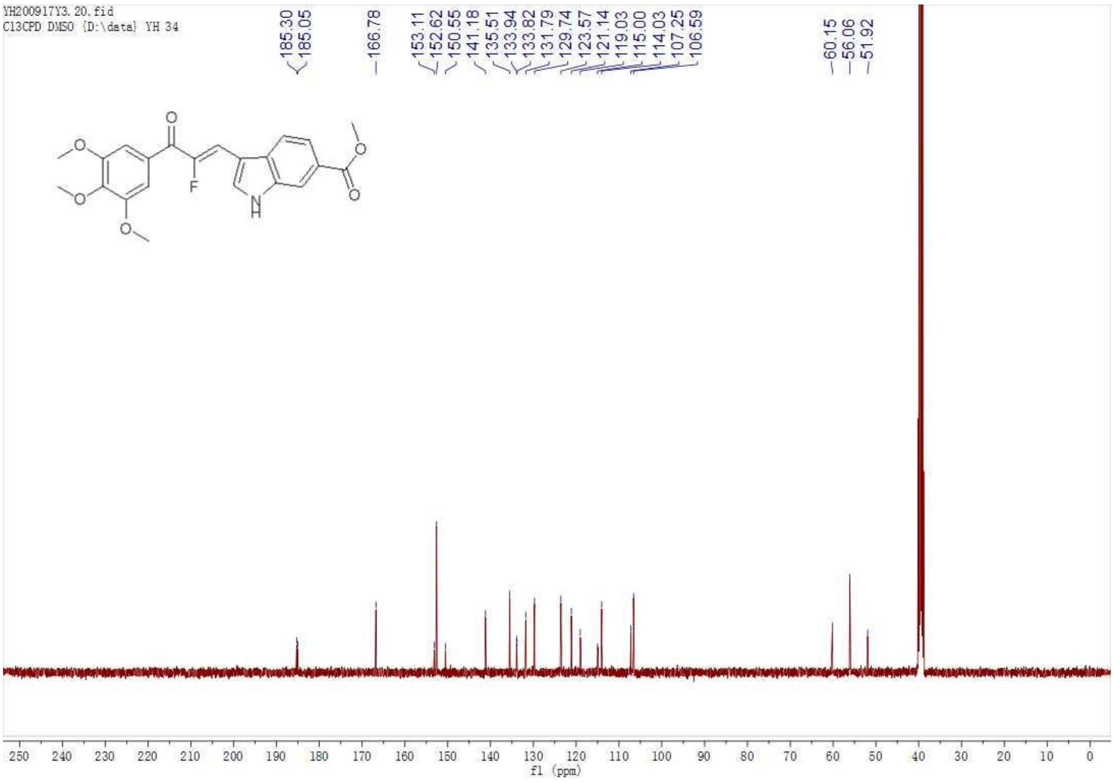

^{13}C NMR of **4a**

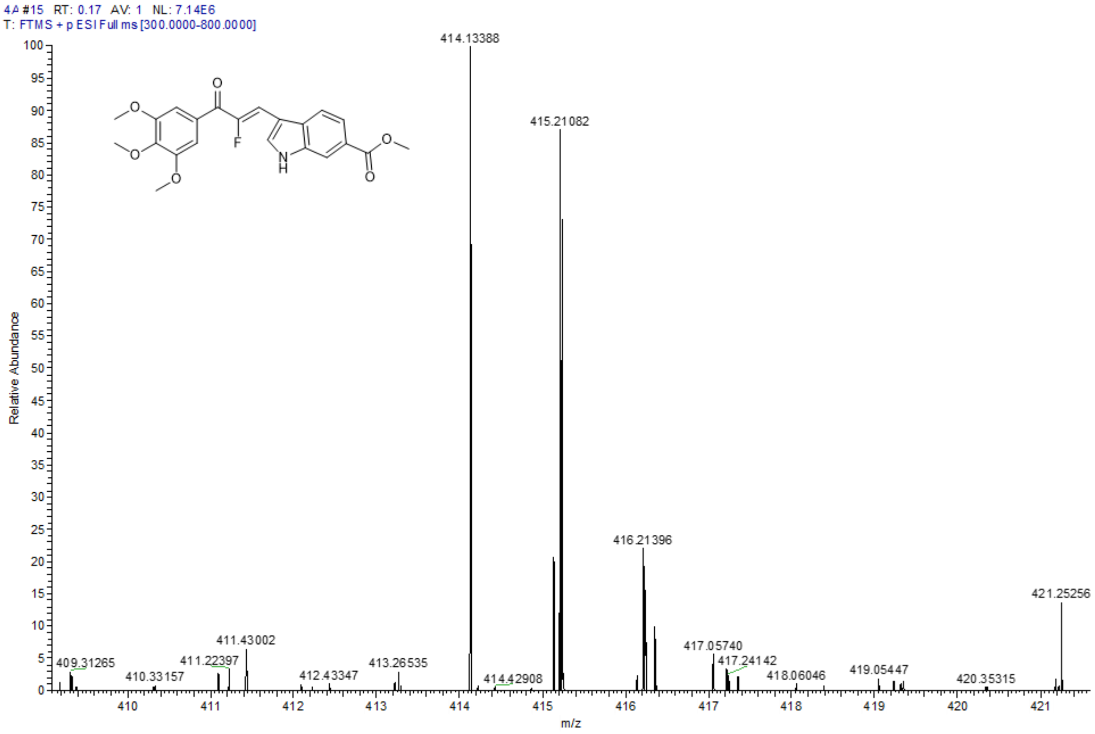

HRMS of **4a**

(2) ^{1}H、^{13}C NMR and HRMS spectra of **4b**

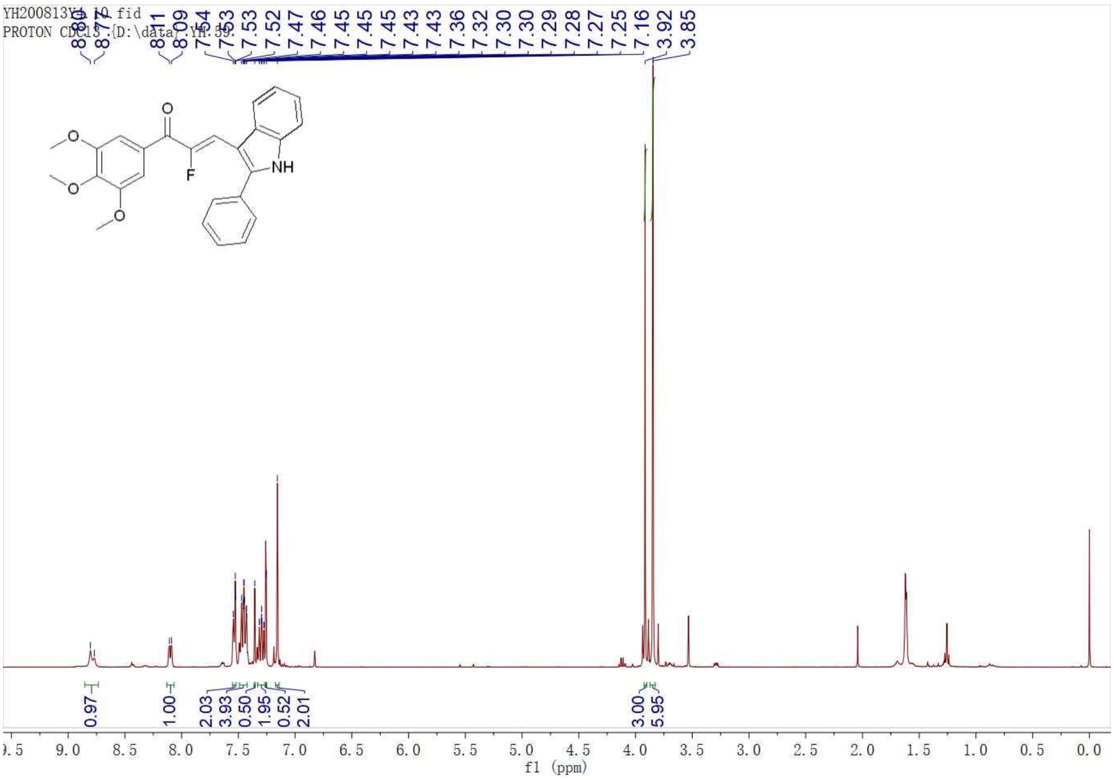

^{1}H NMR of **4b**

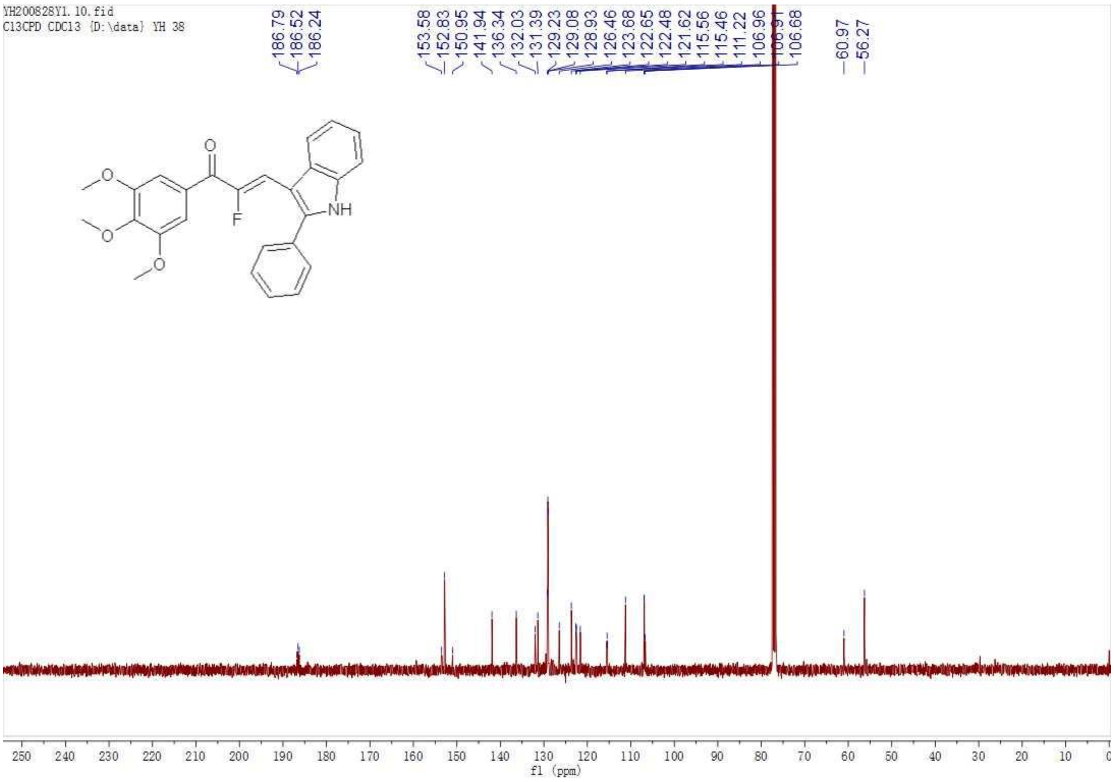

^{13}C NMR of **4b**

\

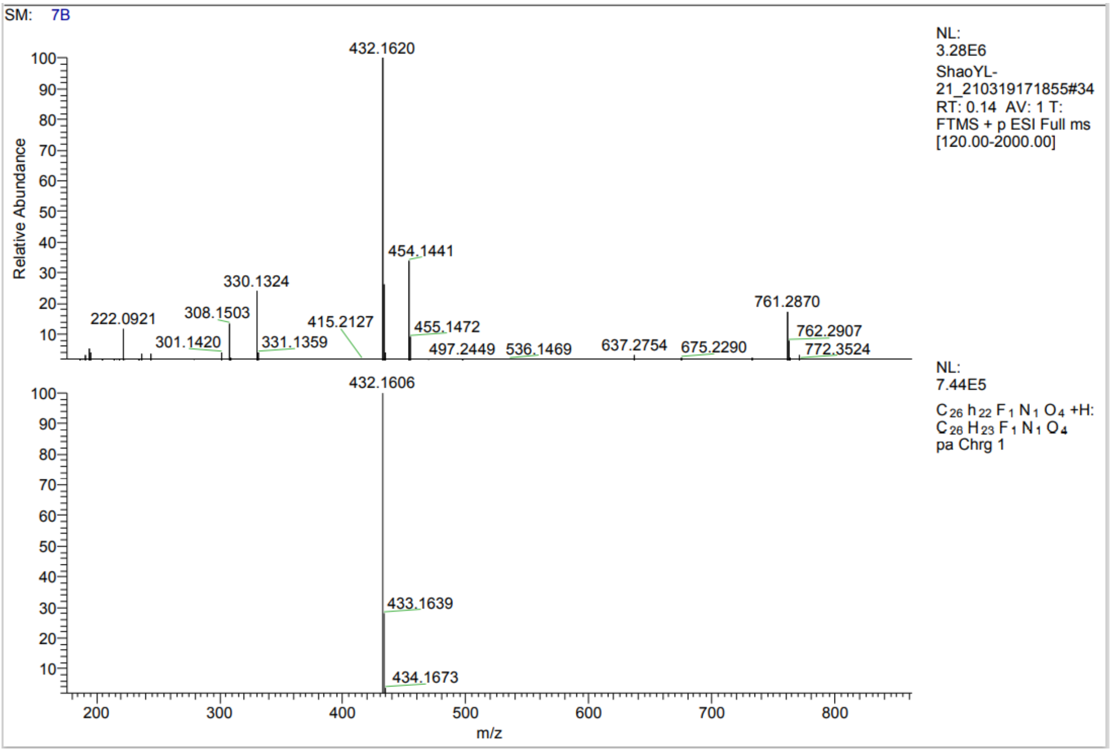

HRMS of **4b**

(3) ^{1}H、^{13}C NMR and HRMS spectra of **4c**

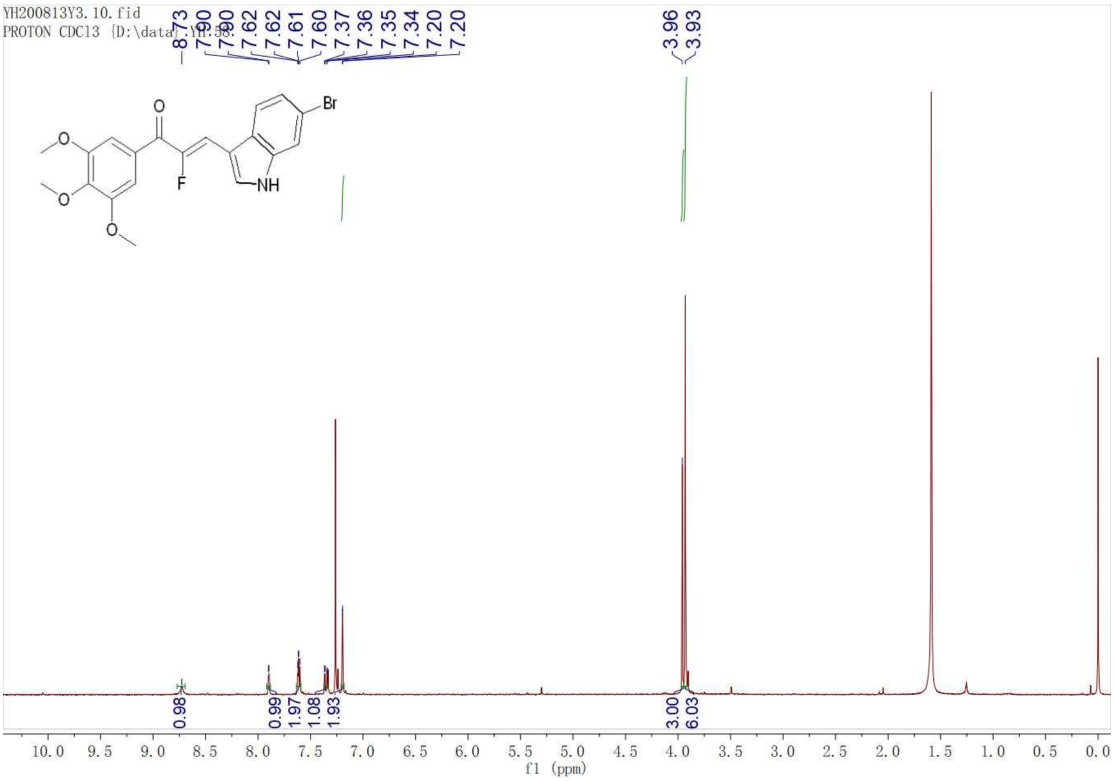

^{1}H NMR of **4c**

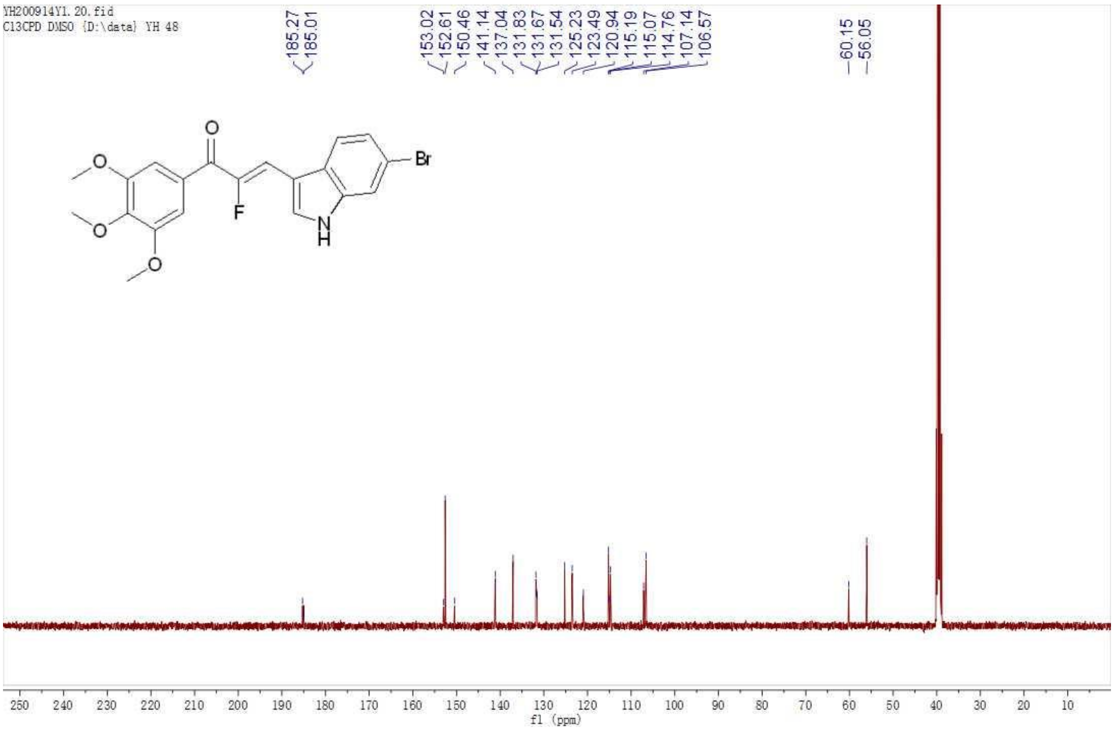

^{13}C NMR of **4c**

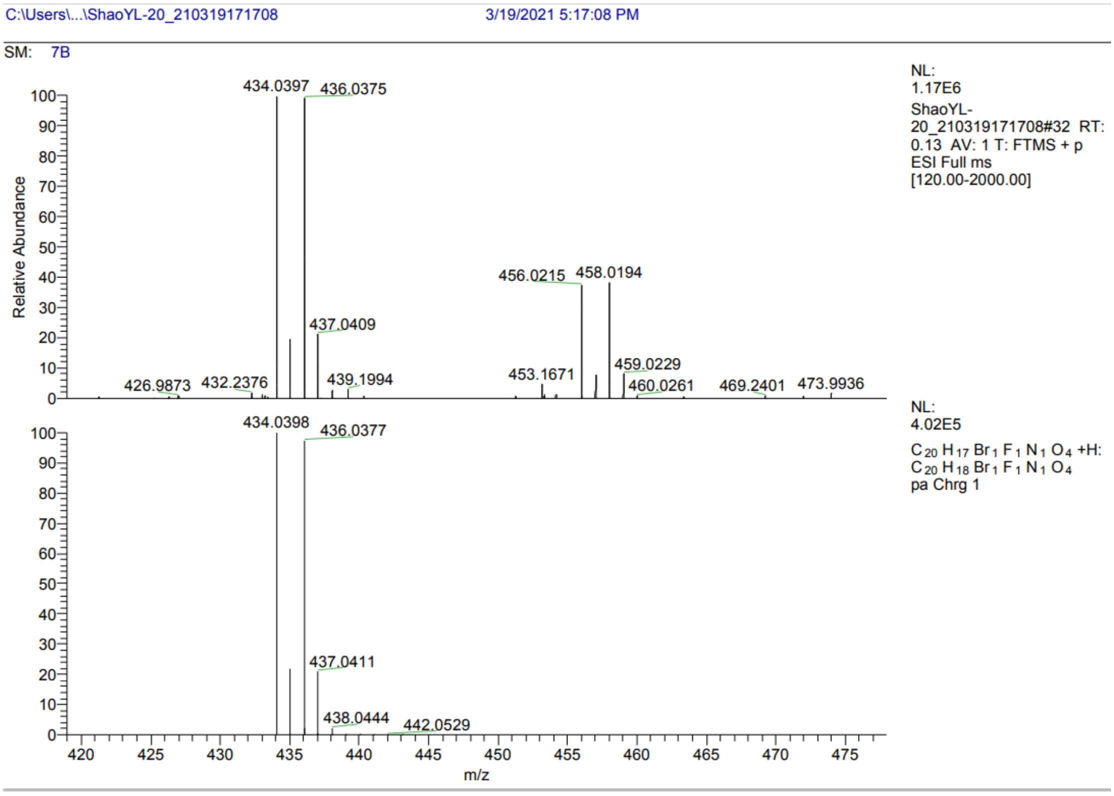

HRMS of **4c**

(4) ^{1}H、^{13}C NMR and HRMS spectra of **4d**

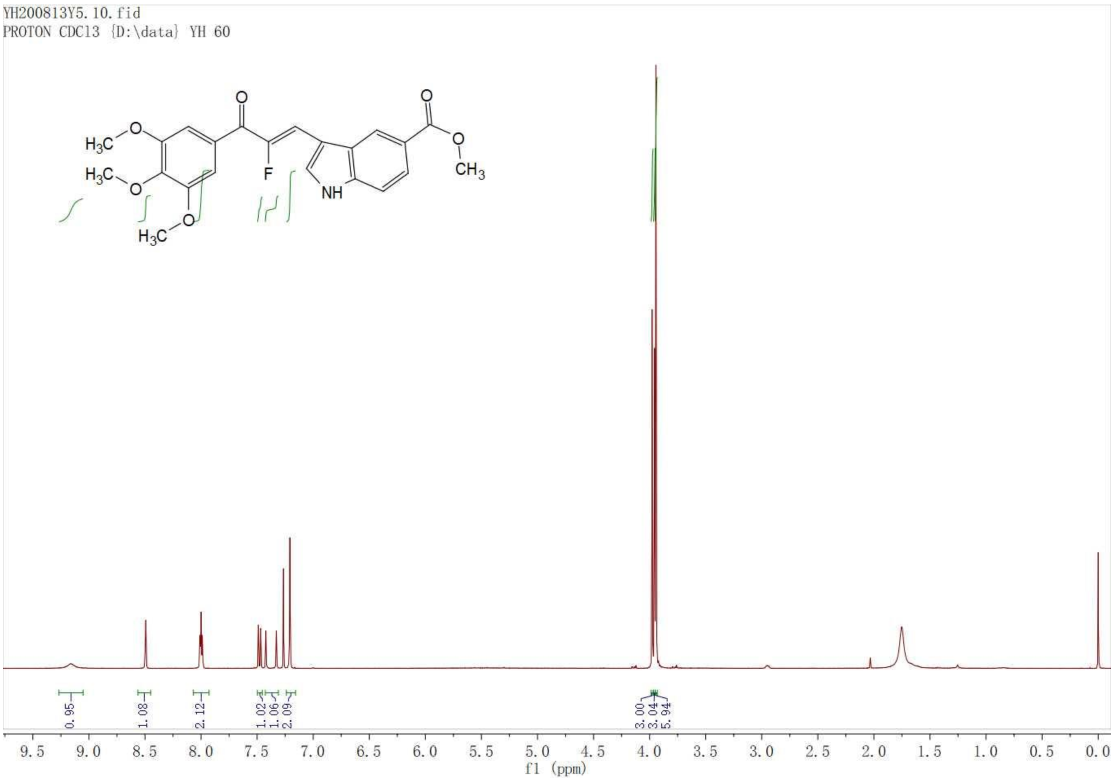

^{1}H NMR of **4d**

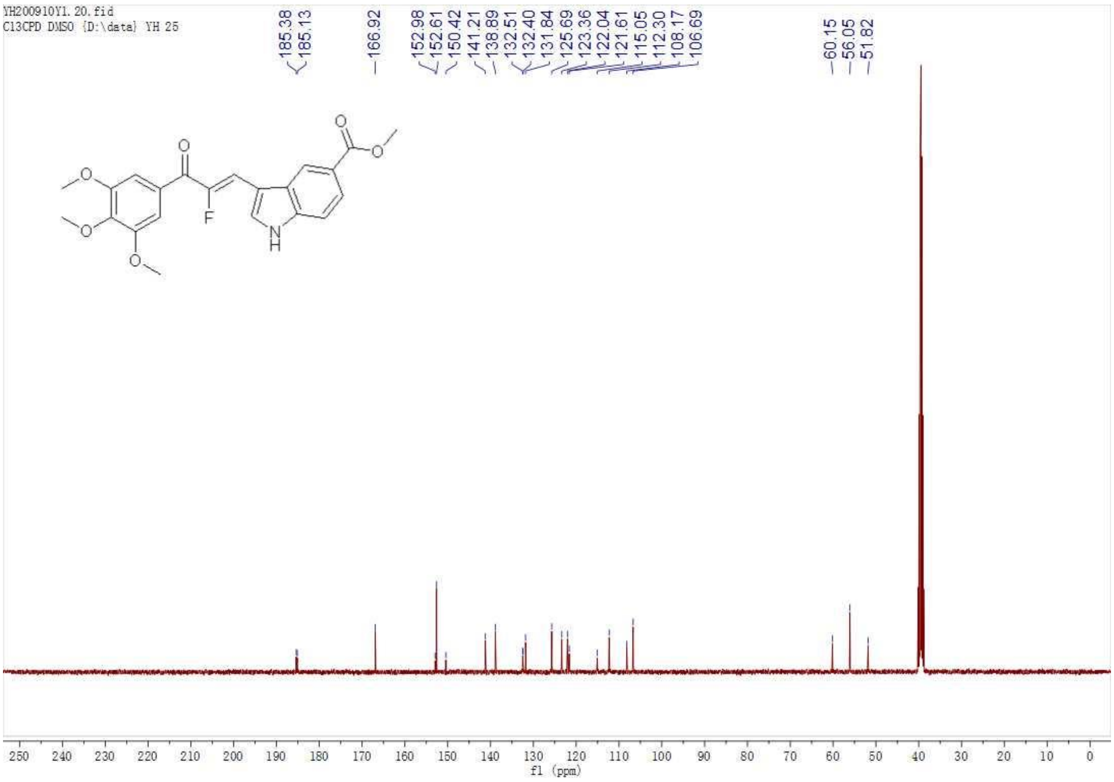

^{13}C NMR of **4d**

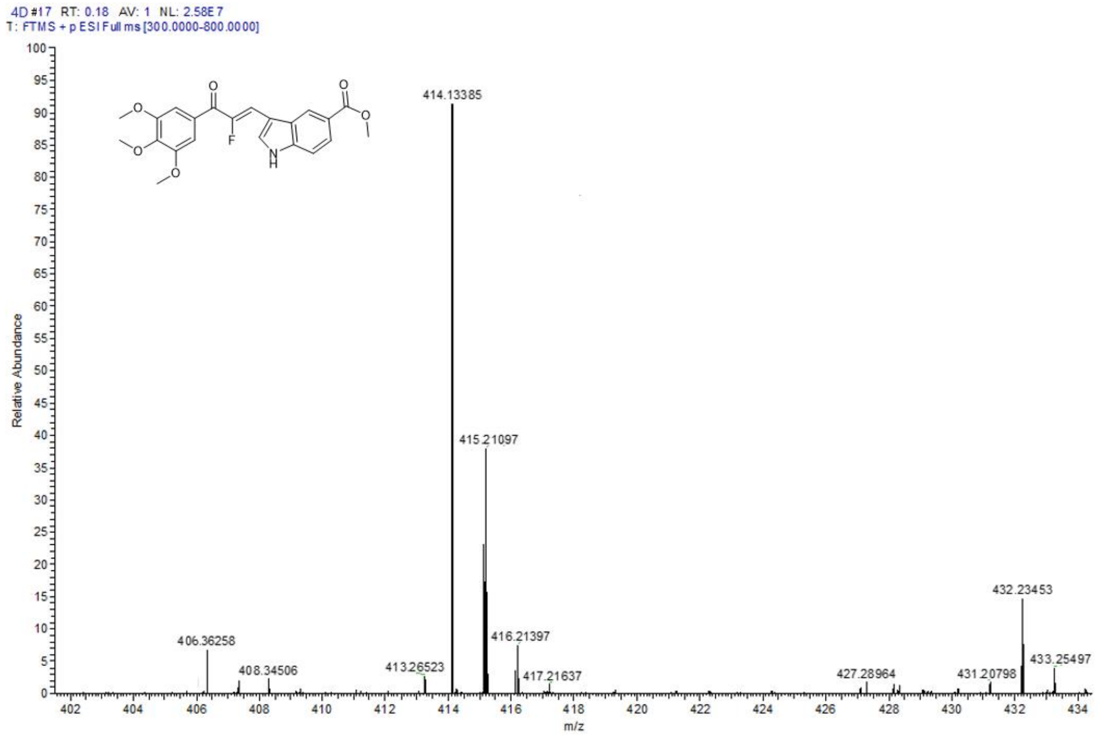

HRMS of **4d**

(5) ^{1}H、^{13}C NMR and HRMS spectra of **4e**

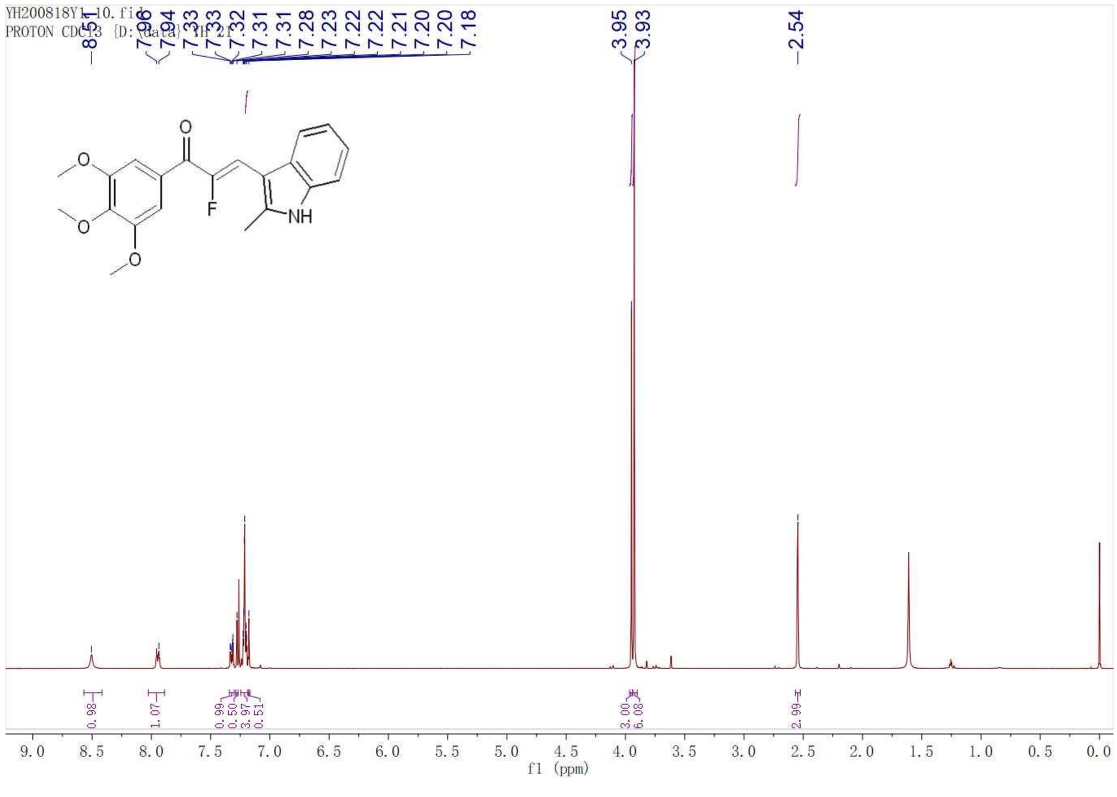

^{1}H NMR of **4e**

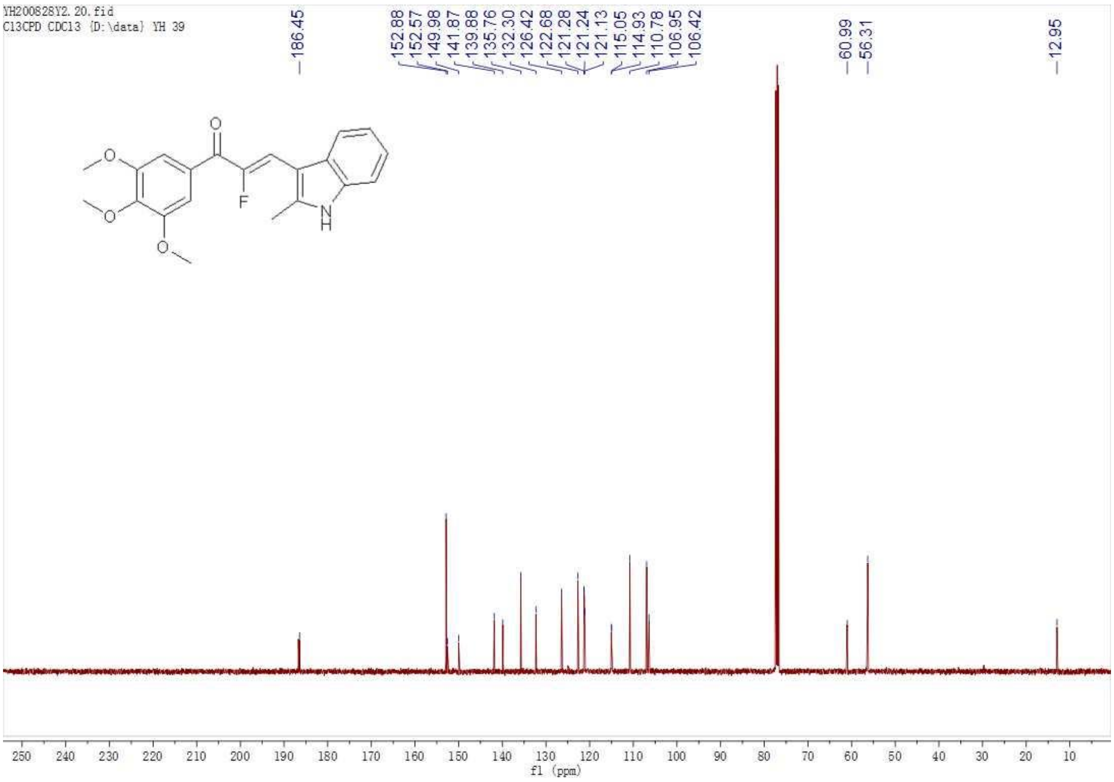

^{13}C NMR of **4e**

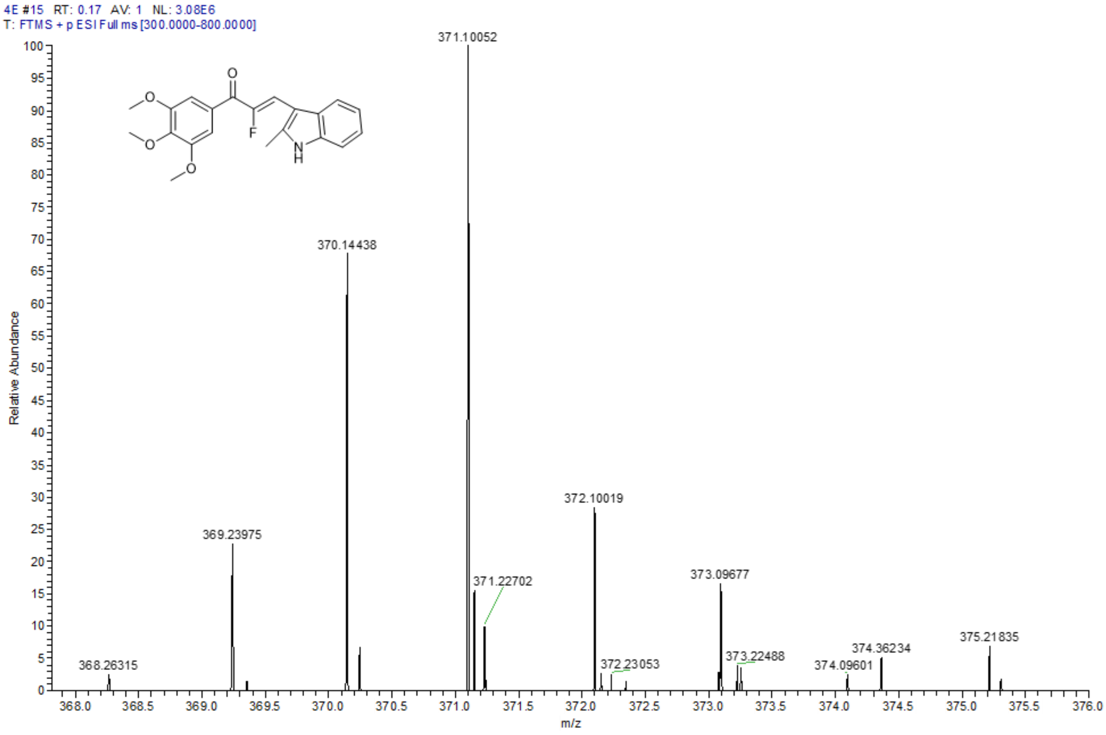

HRMS of **4e**

(6) ^{1}H、^{13}C NMR and HRMS spectra of **4f**

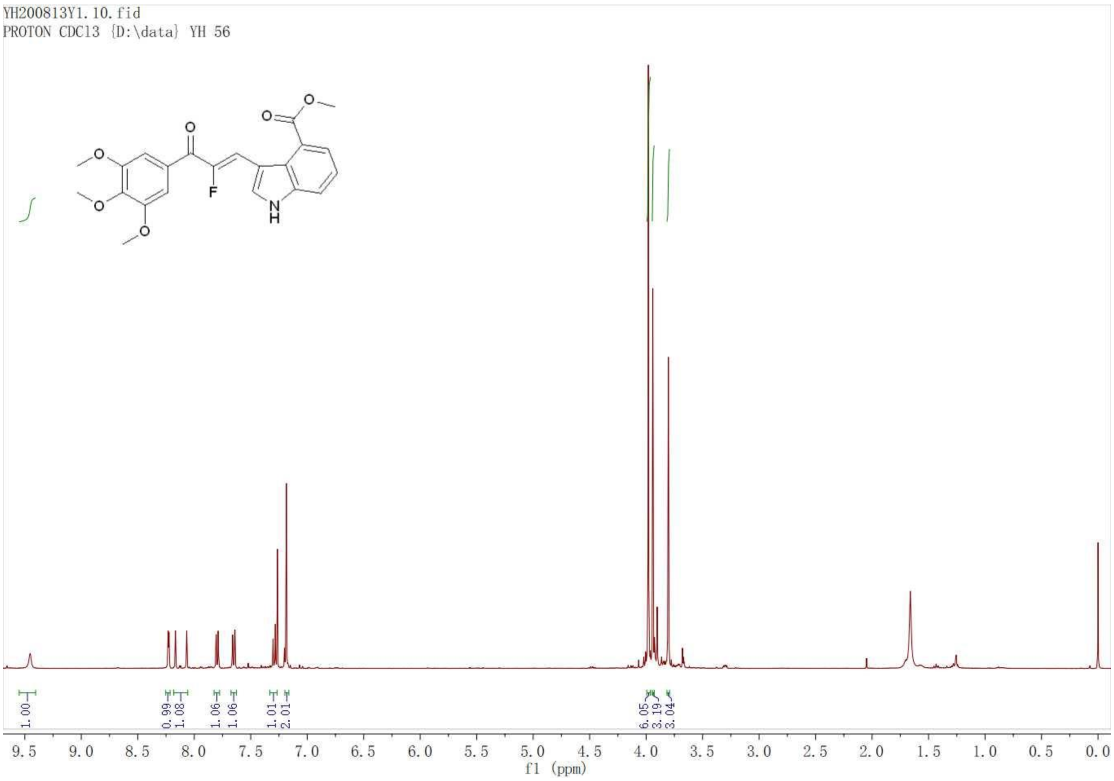

^{1}H NMR of **4f**

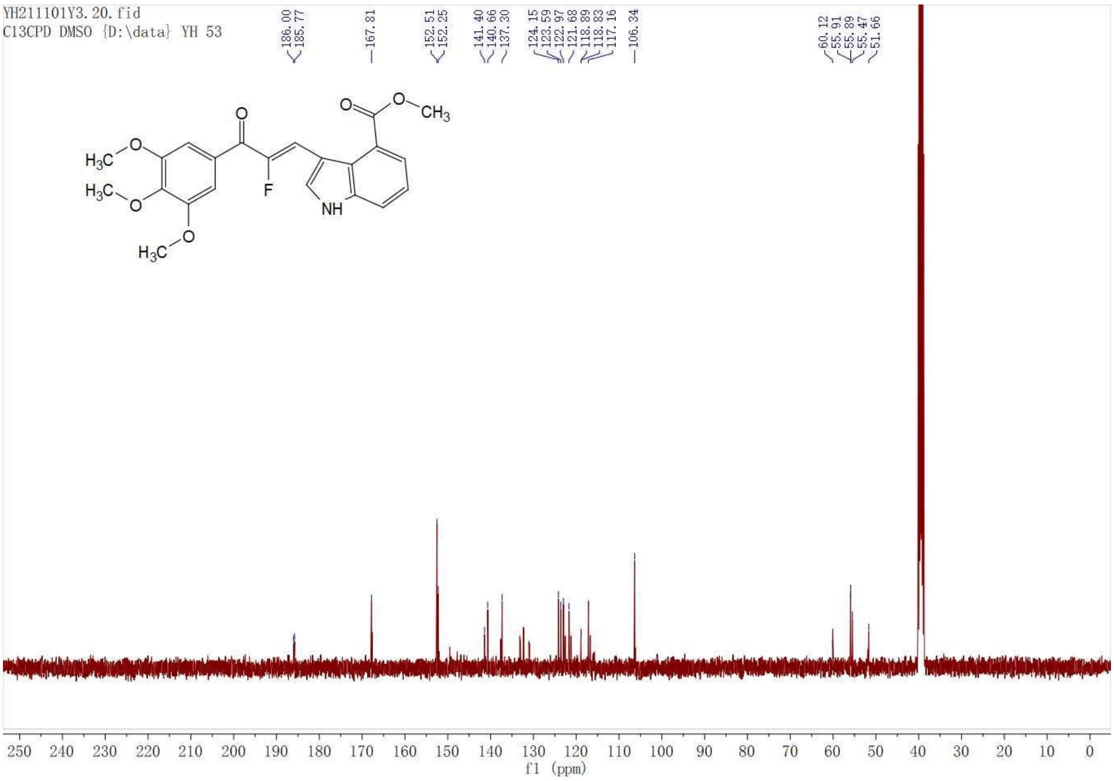

^{13}C NMR of **4f**

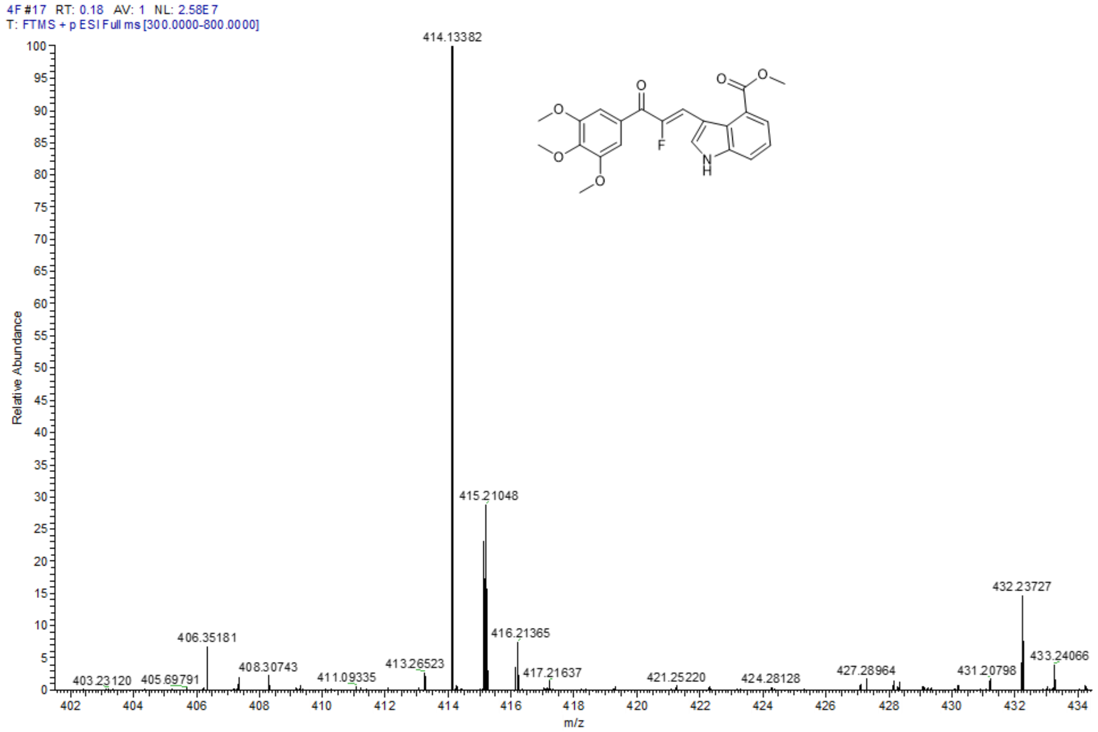

HRMS of **4f**

(7) ^{1}H、^{13}C NMR and HRMS spectra of **4g**

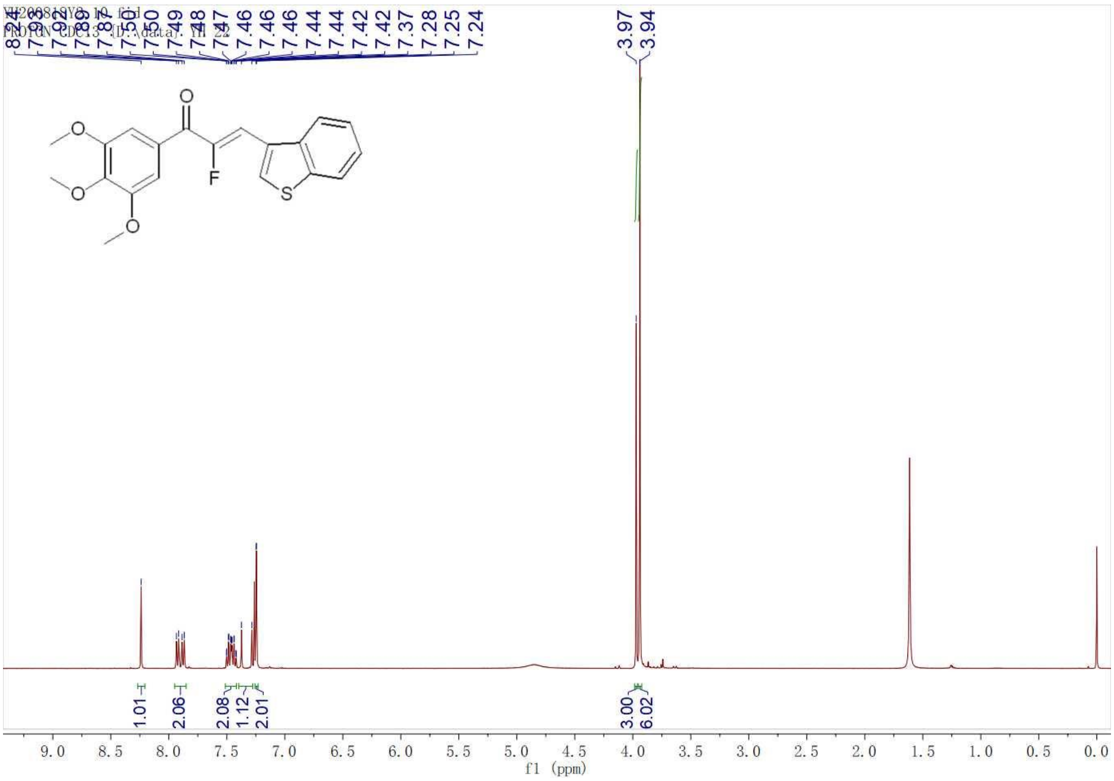

^{1}H NMR of **4g**

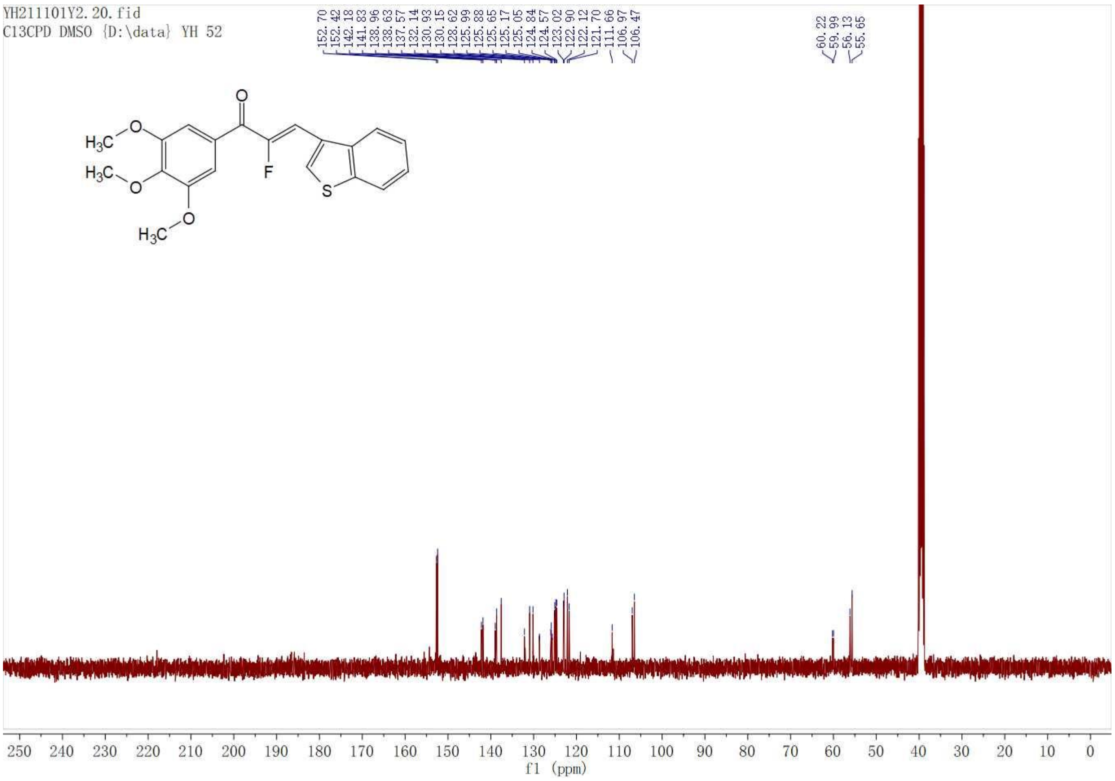

^{13}C NMR of **4g**

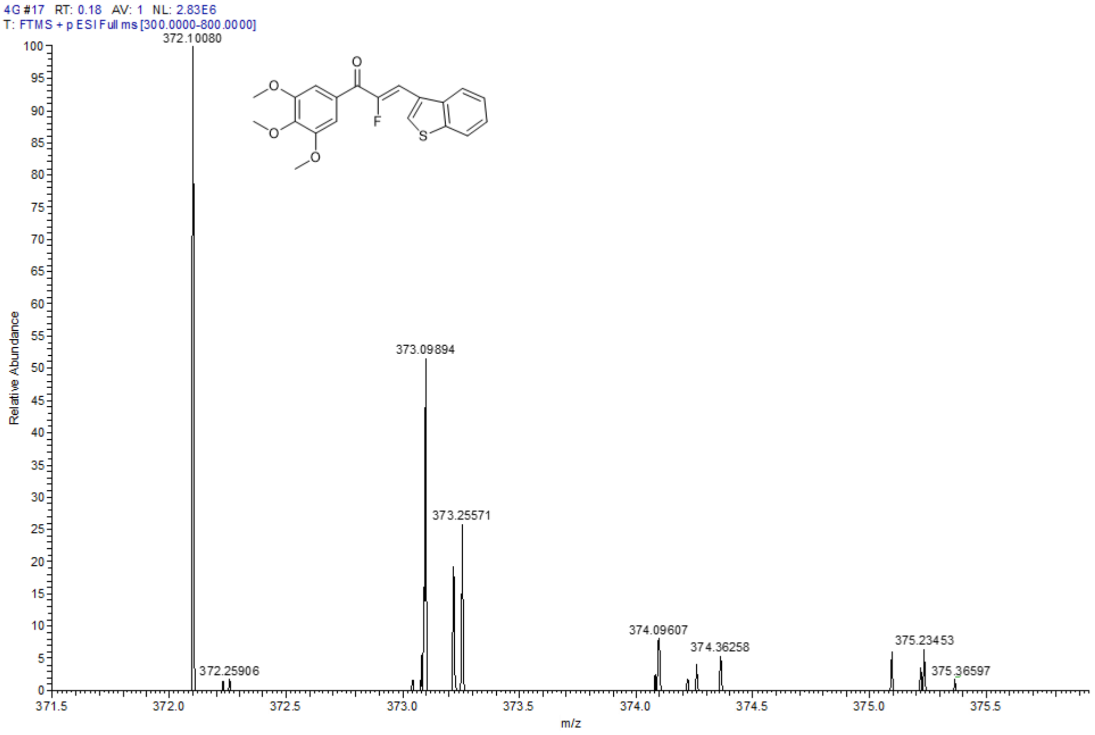

HRMS of **4g**

(8) ^{1}H、^{13}C NMR and HRMS spectra of **4h**

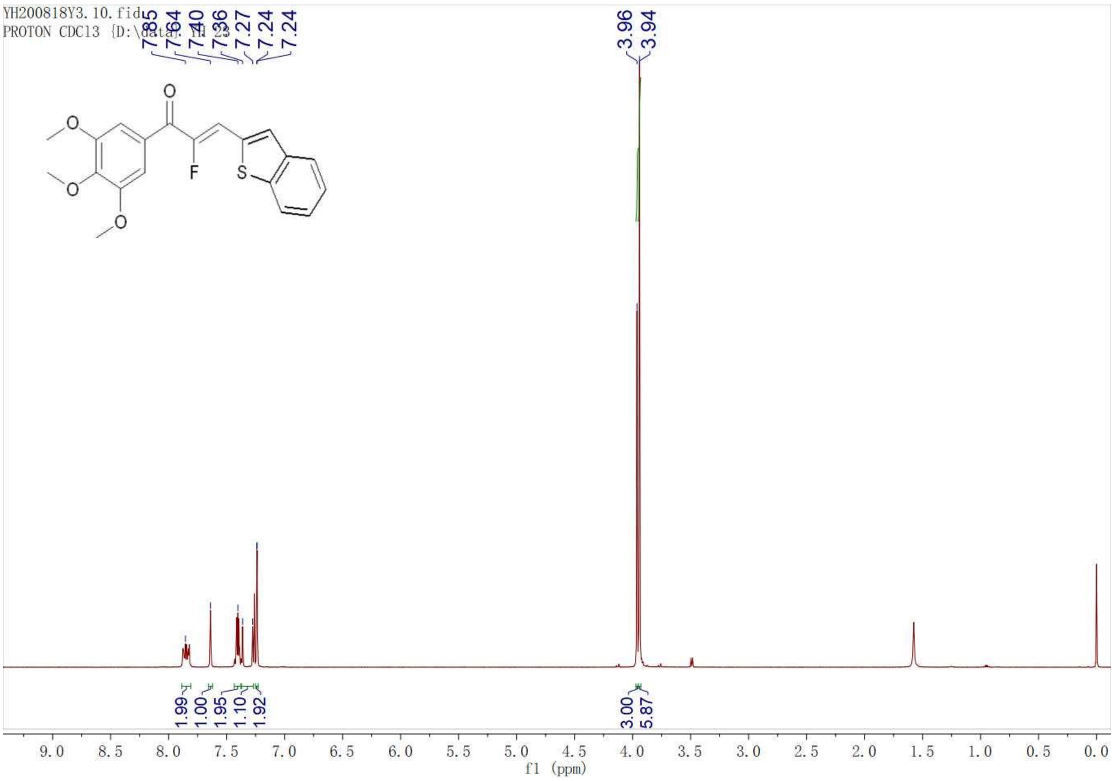

^{1}H NMR of **4h**

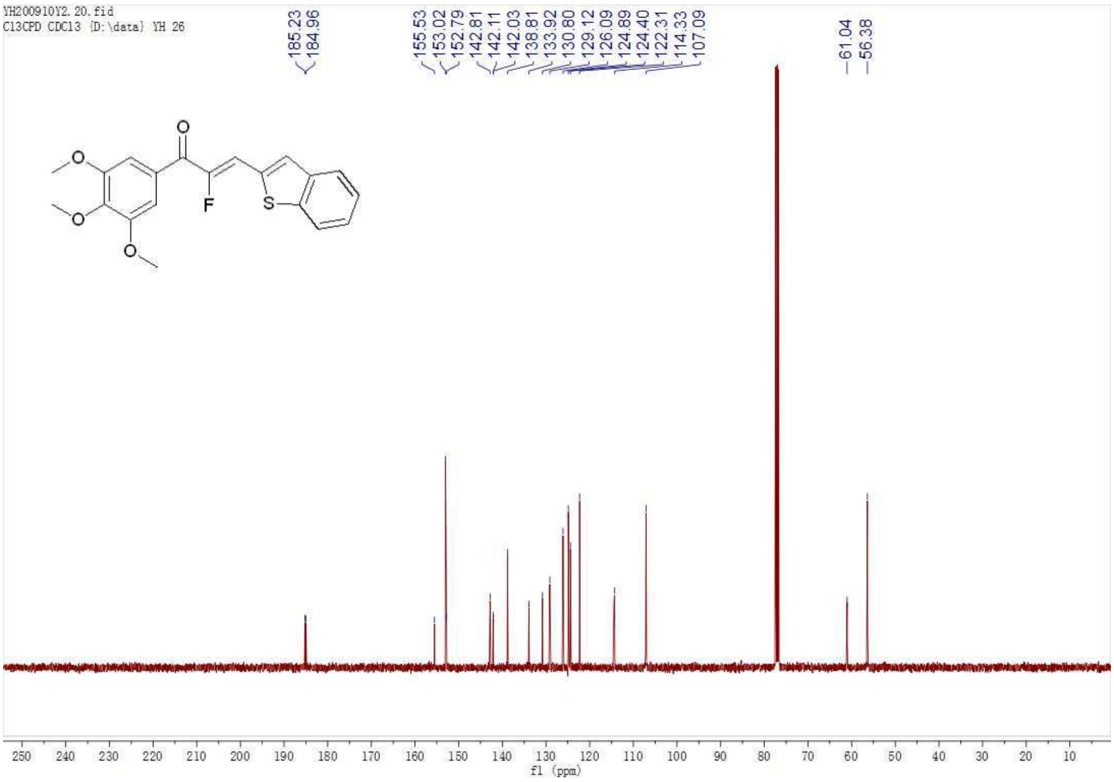

^{13}C NMR of **4h**

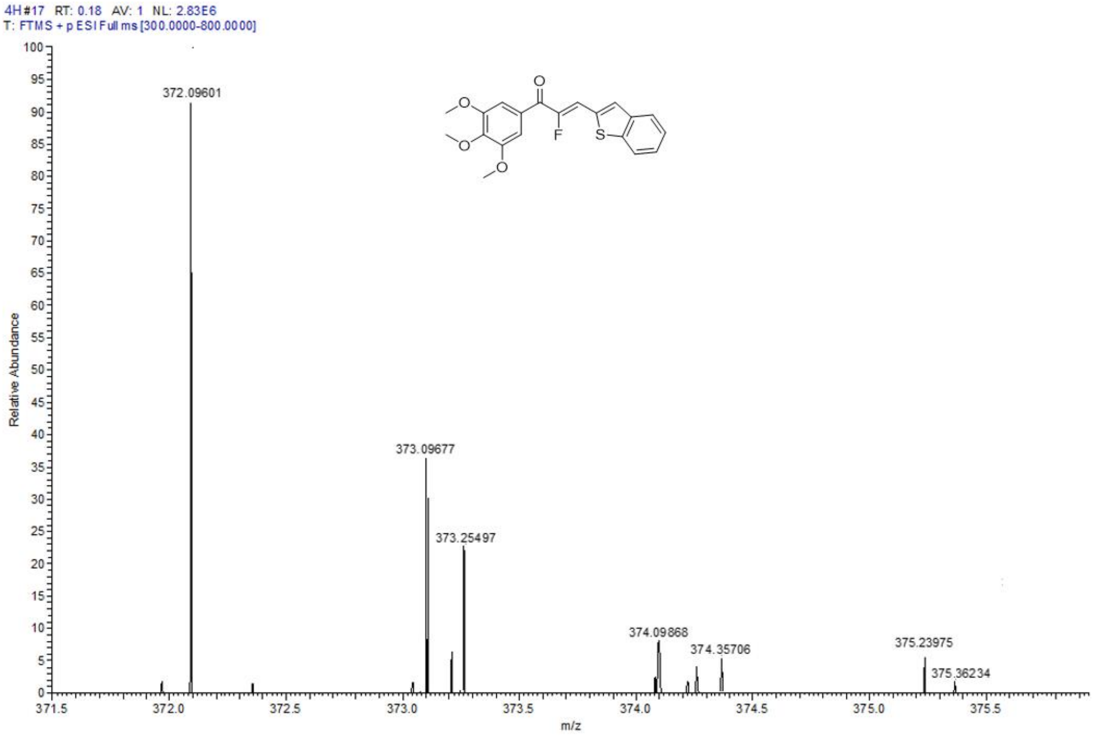

HRMS of **4h**

(9) ^{1}H、^{13}C NMR and HRMS spectra of **4i**

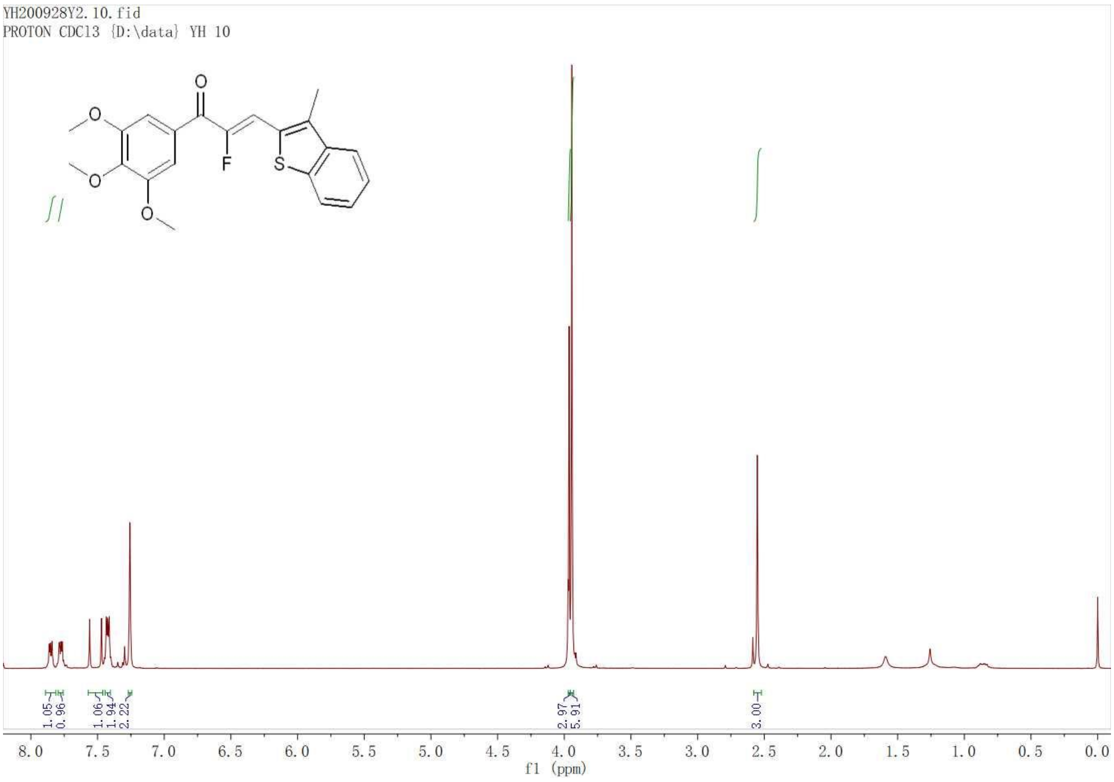

^{1}H NMR of **4i**

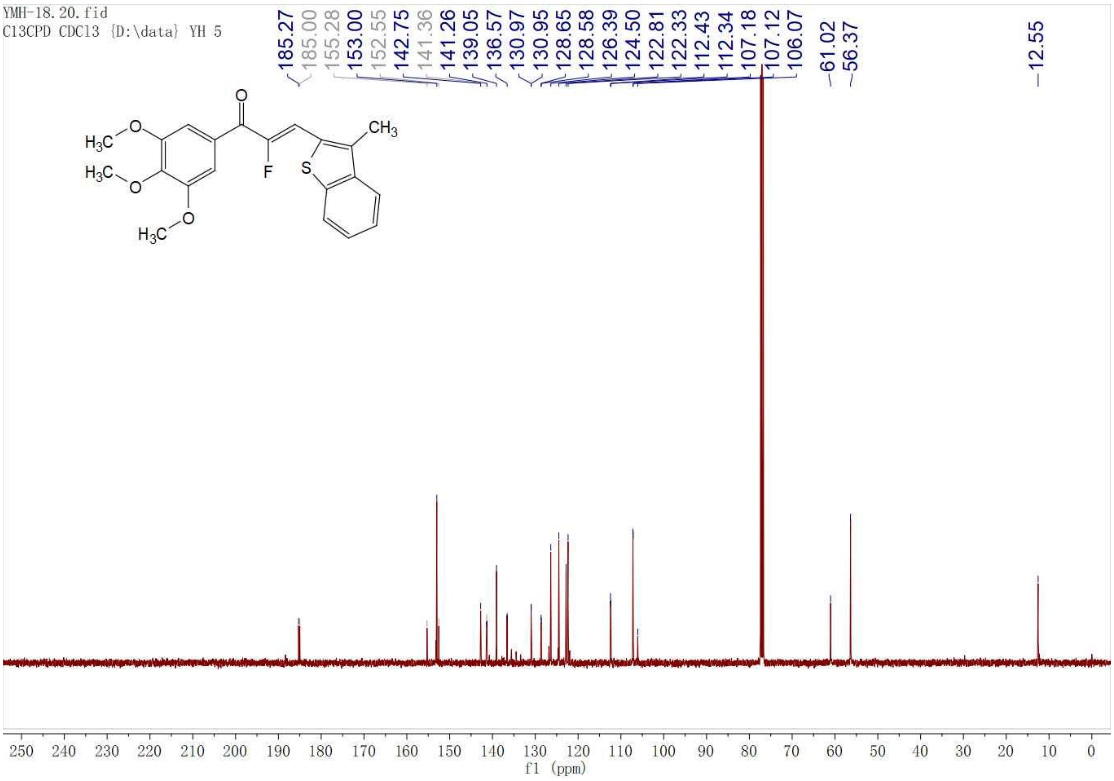

^{13}C NMR of **4i**

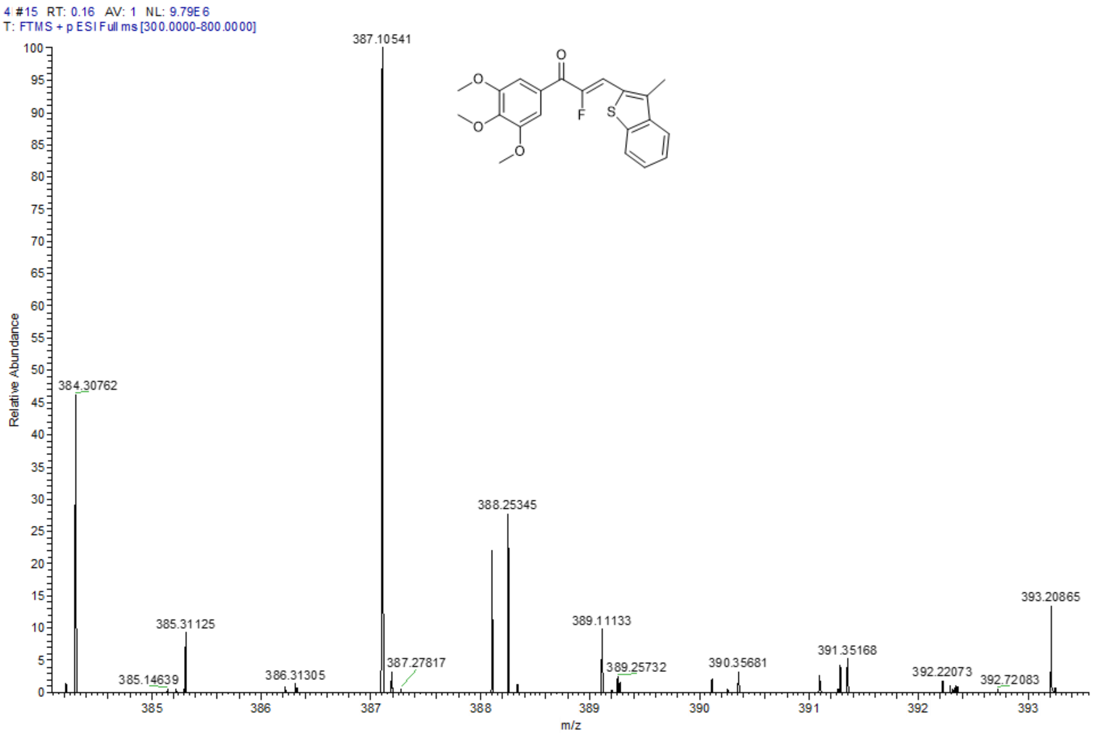

HRMS of **4i**

(10) ^{1}H、^{13}C NMR and HRMS spectra of **4j**

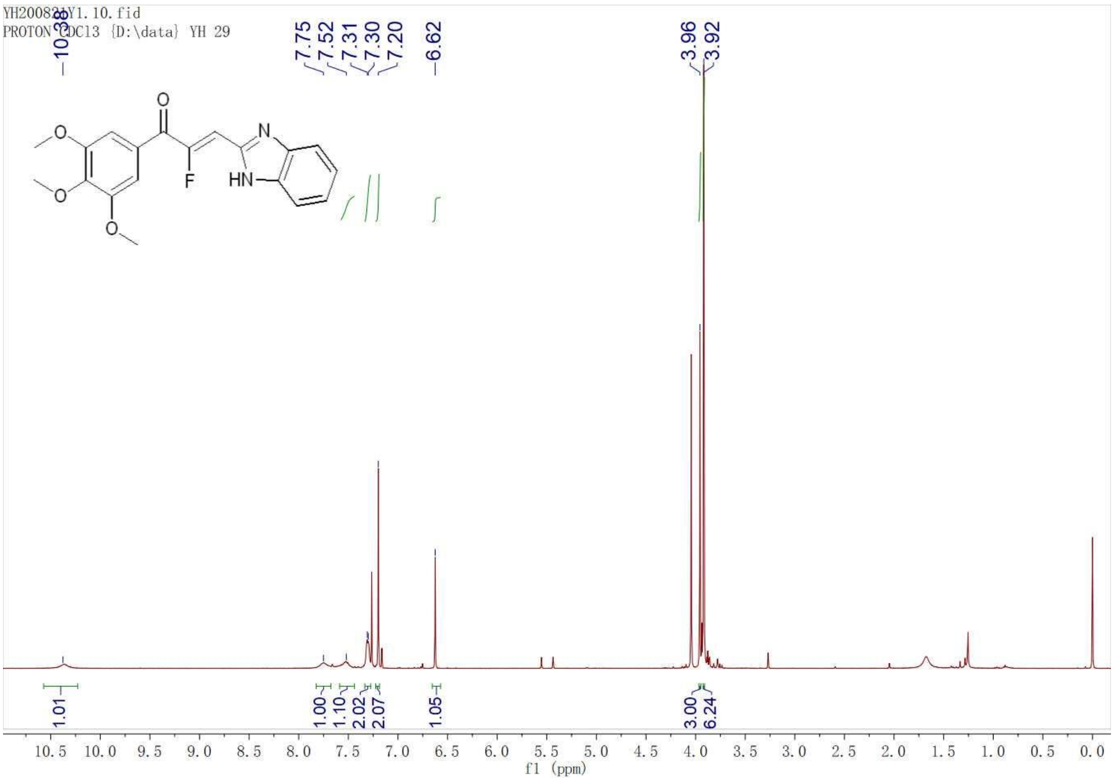

^{1}H NMR of **4j**

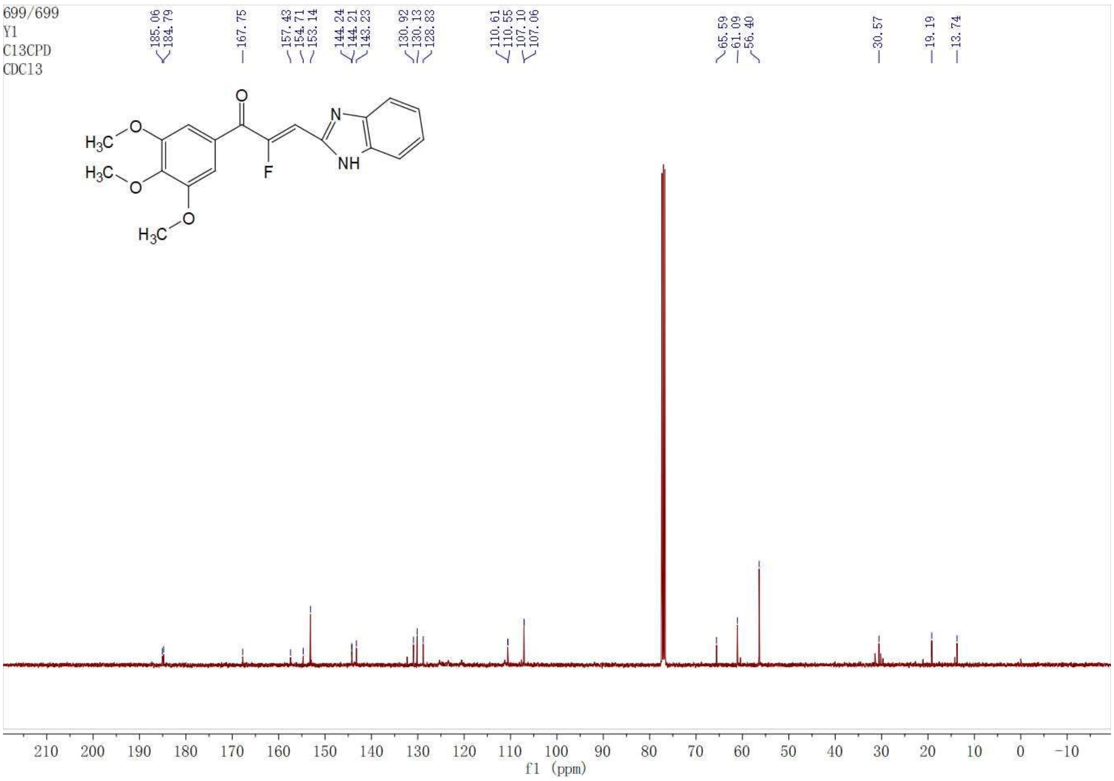

^{13}C NMR of **4j**

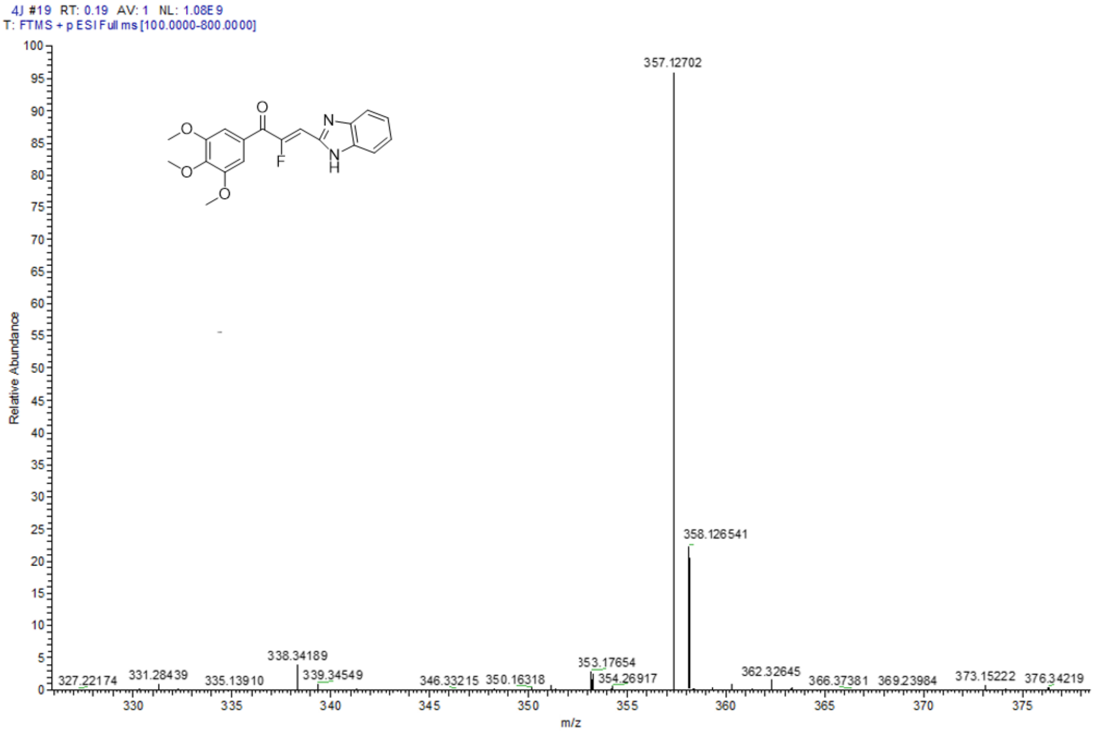

HRMS of **4j**

(11) ^{1}H、^{13}C NMR and HRMS spectra of **4k**

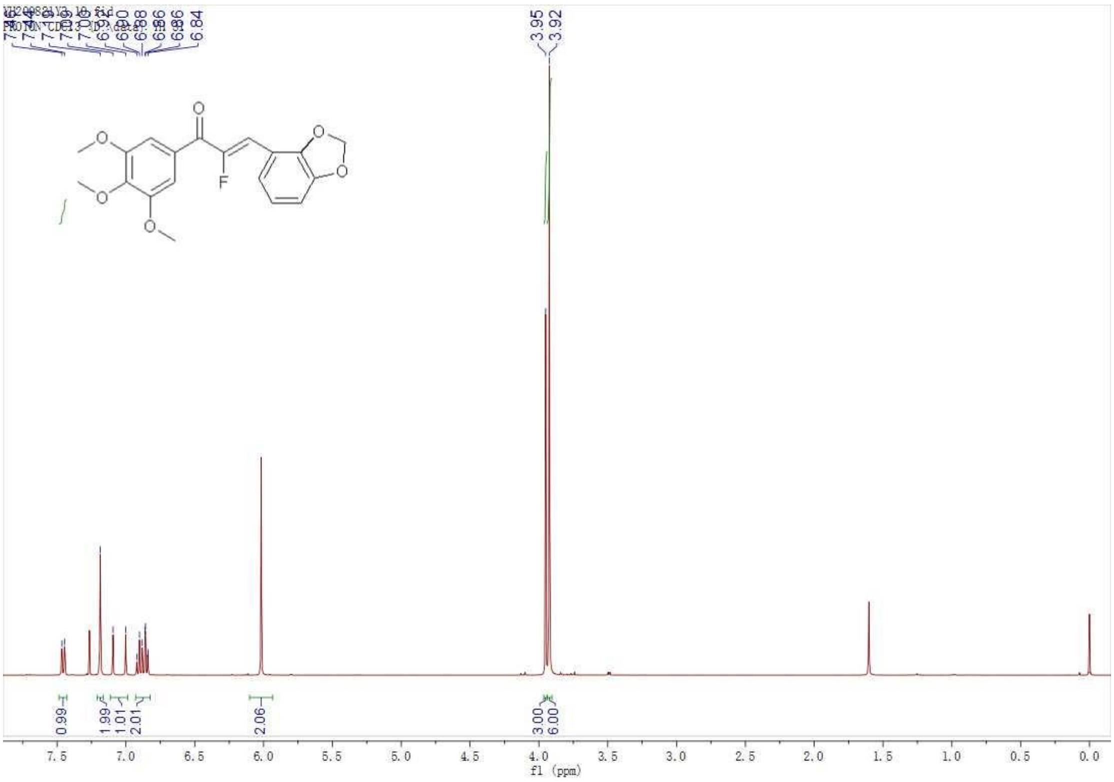

^{1}H NMR of **4k**

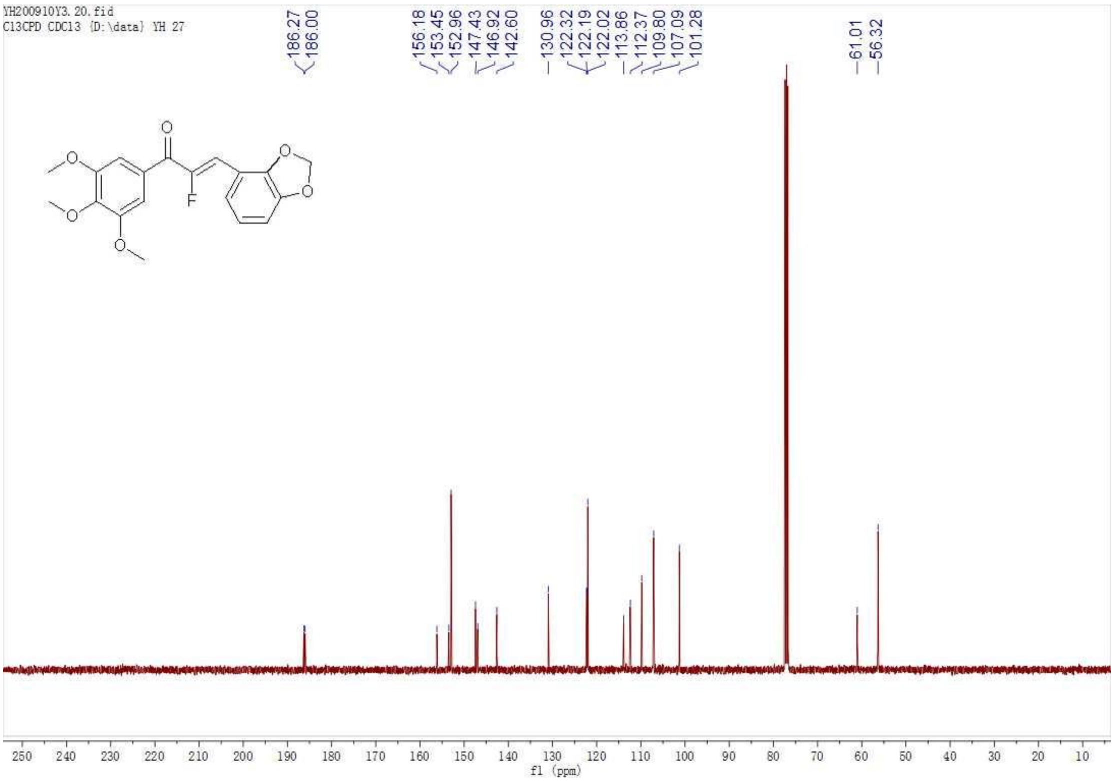

^{13}C NMR of **4k**

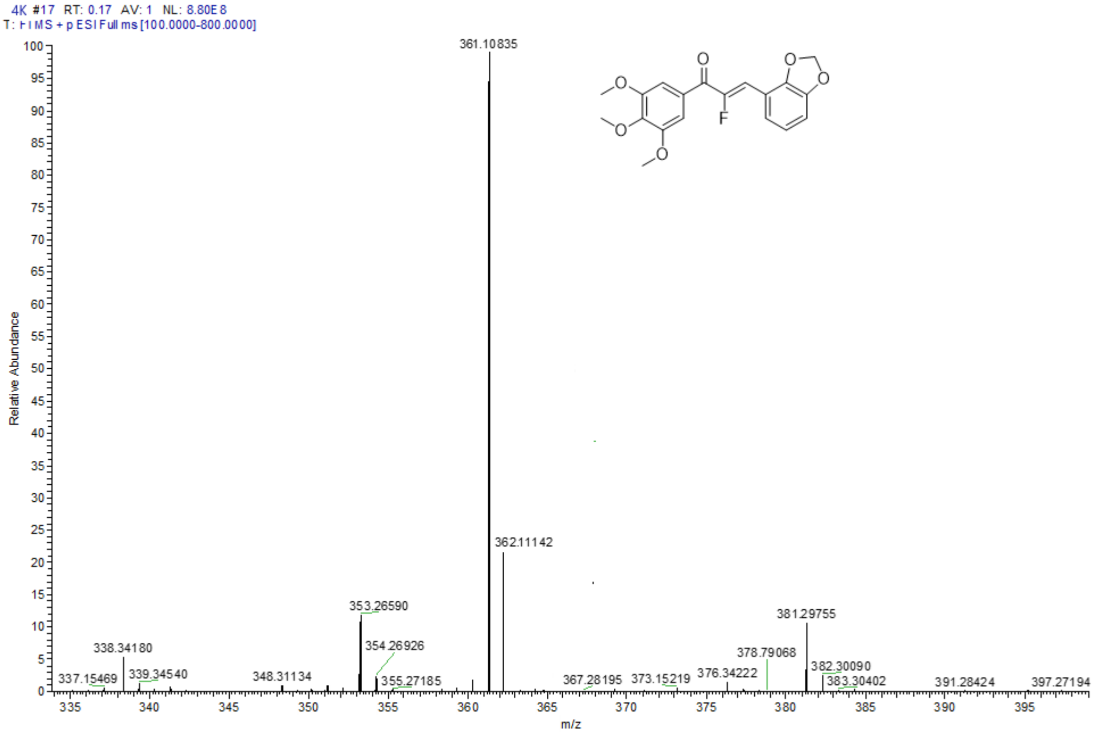

HRMS of **4k**

(12) ^{1}H、^{13}C NMR and HRMS spectra of **4l**

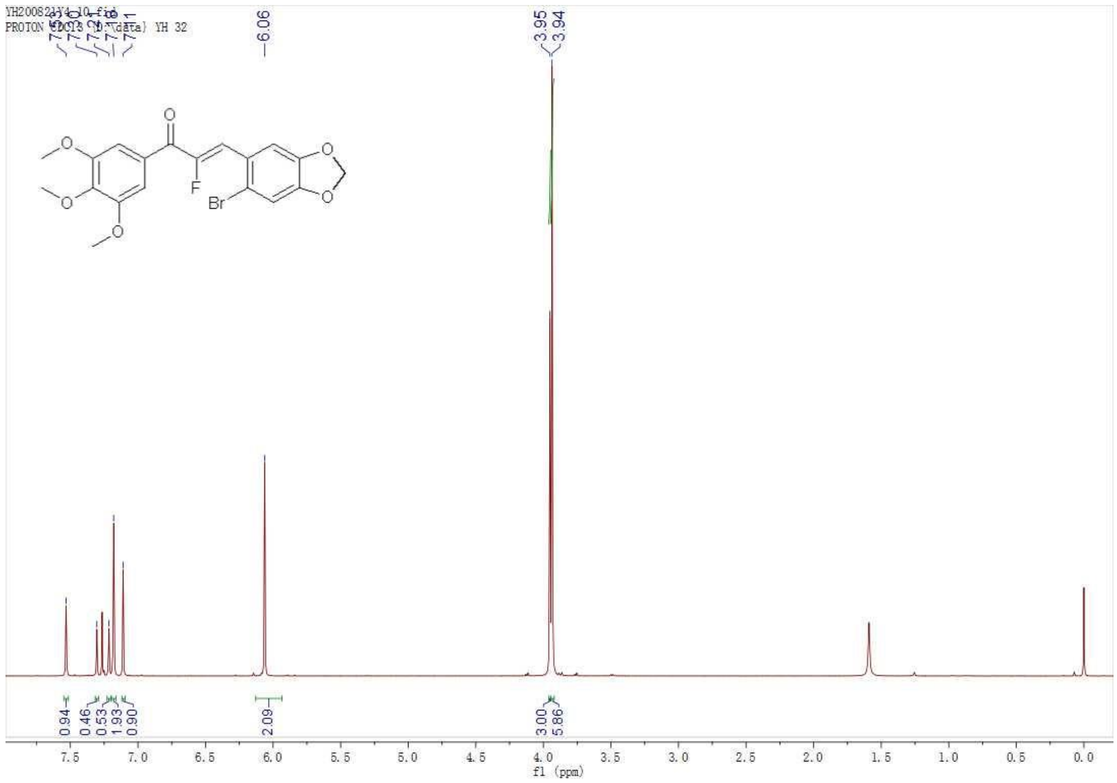

^{1}H NMR of **4l**

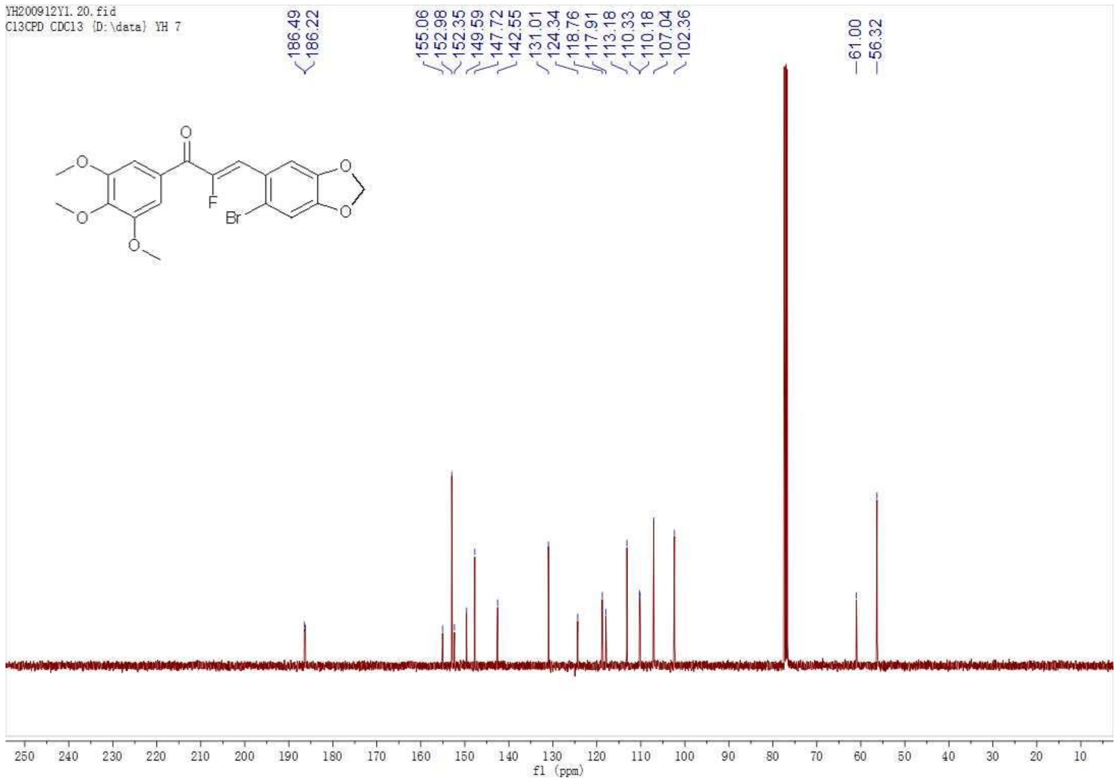

^{13}C NMR of **4l**

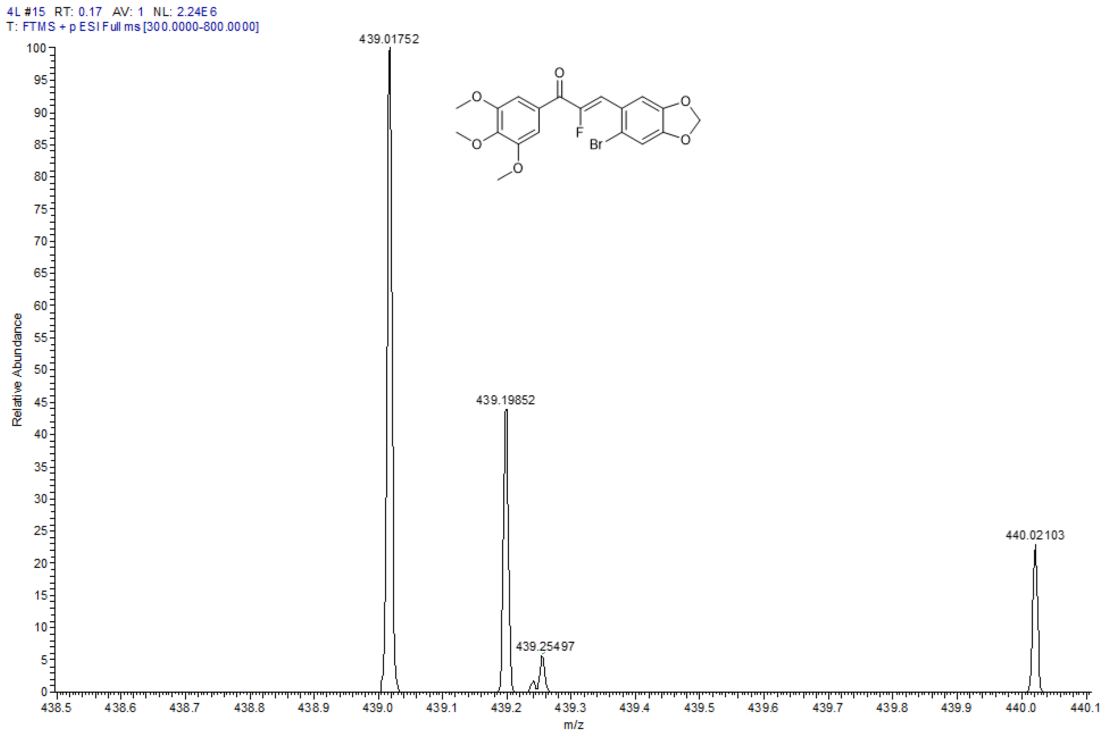

HRMS of **4l**

(13) ¹H、 ¹³C NMR and HRMS spectra of **4m**

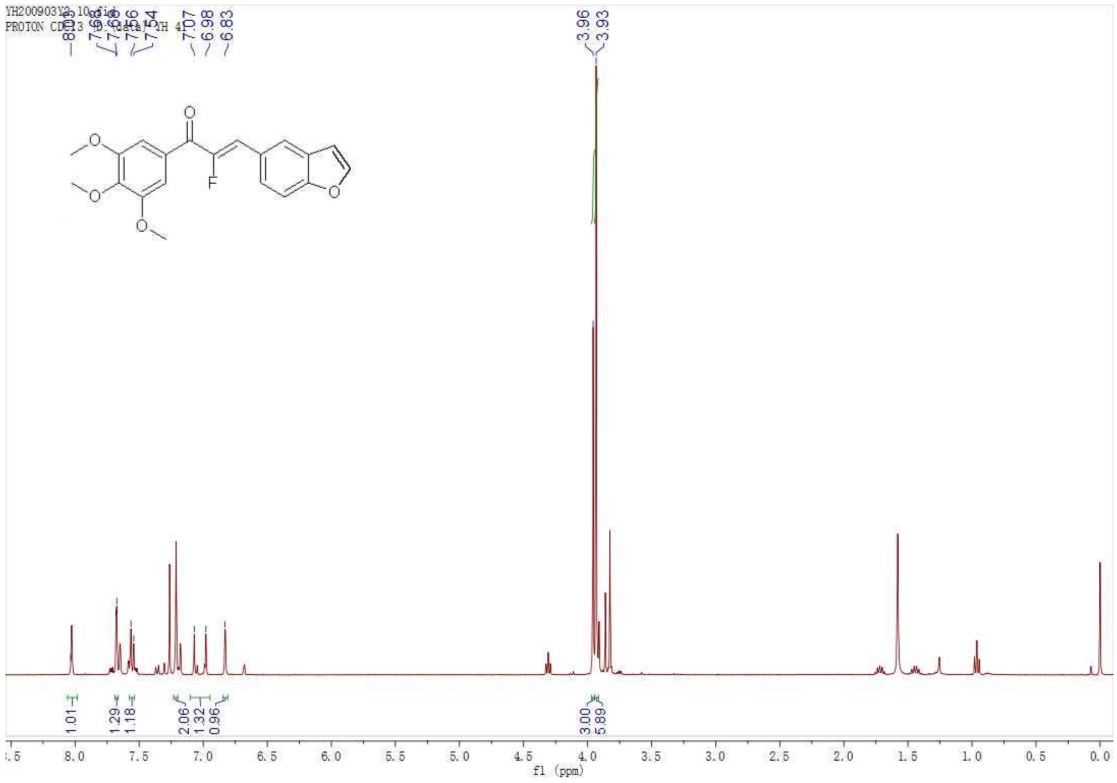

¹H NMR of **4m**

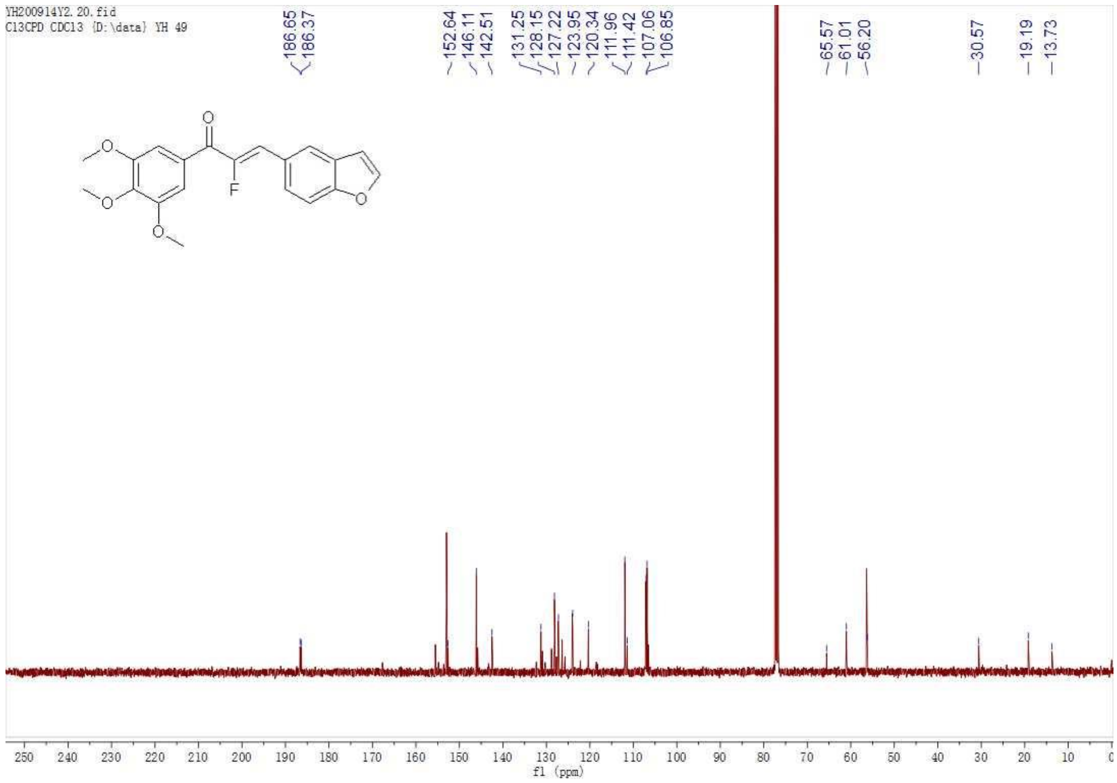

^{13}C NMR of **4m**

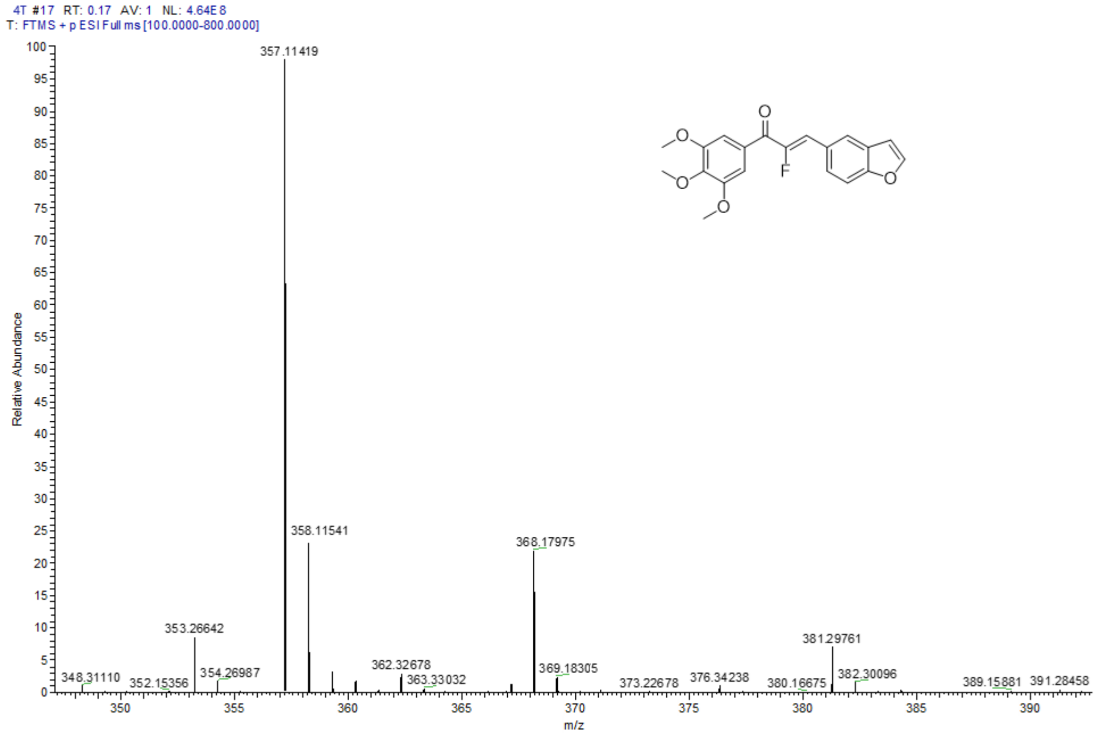

HRMS of **4m**

(14) ^{1}H、^{13}C NMR and HRMS spectra of **4n**

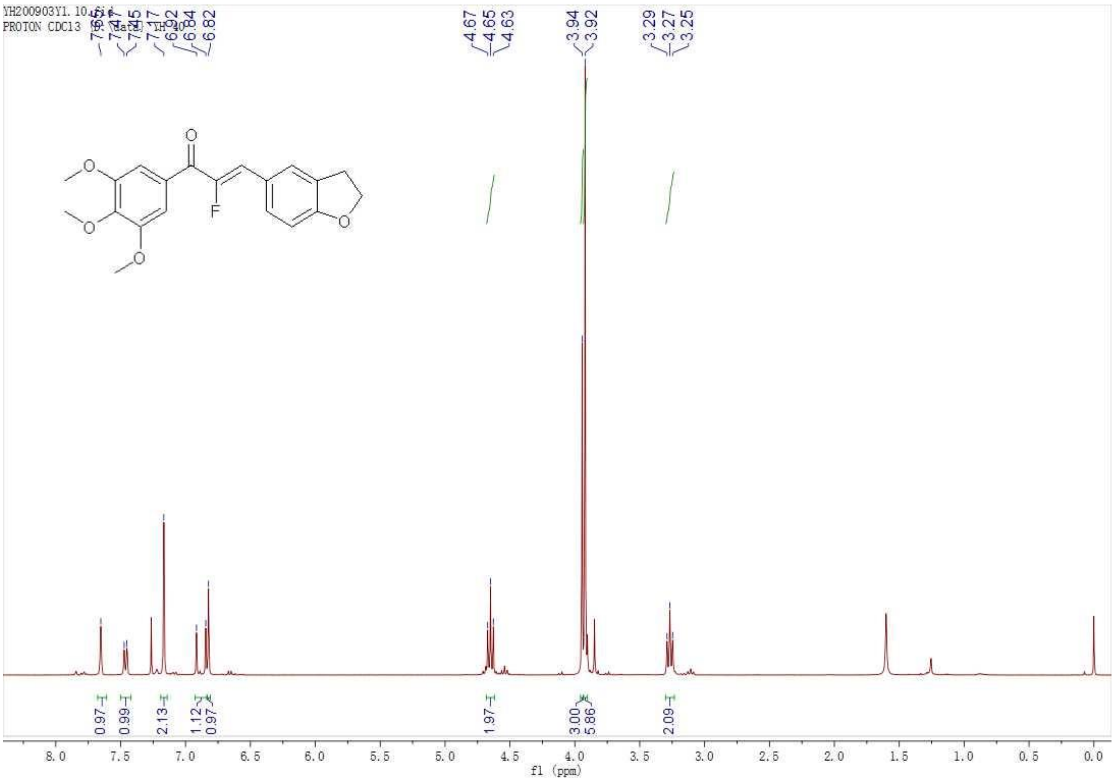

^{1}H NMR of **4n**

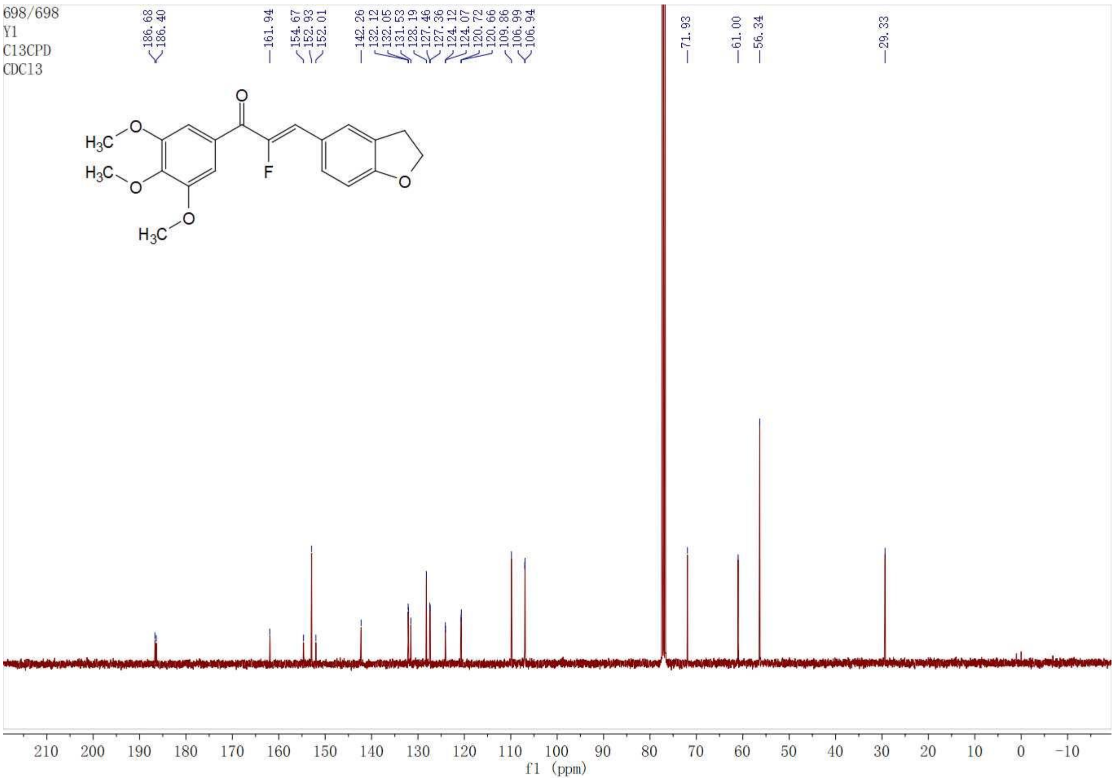

^{13}C NMR of **4n**

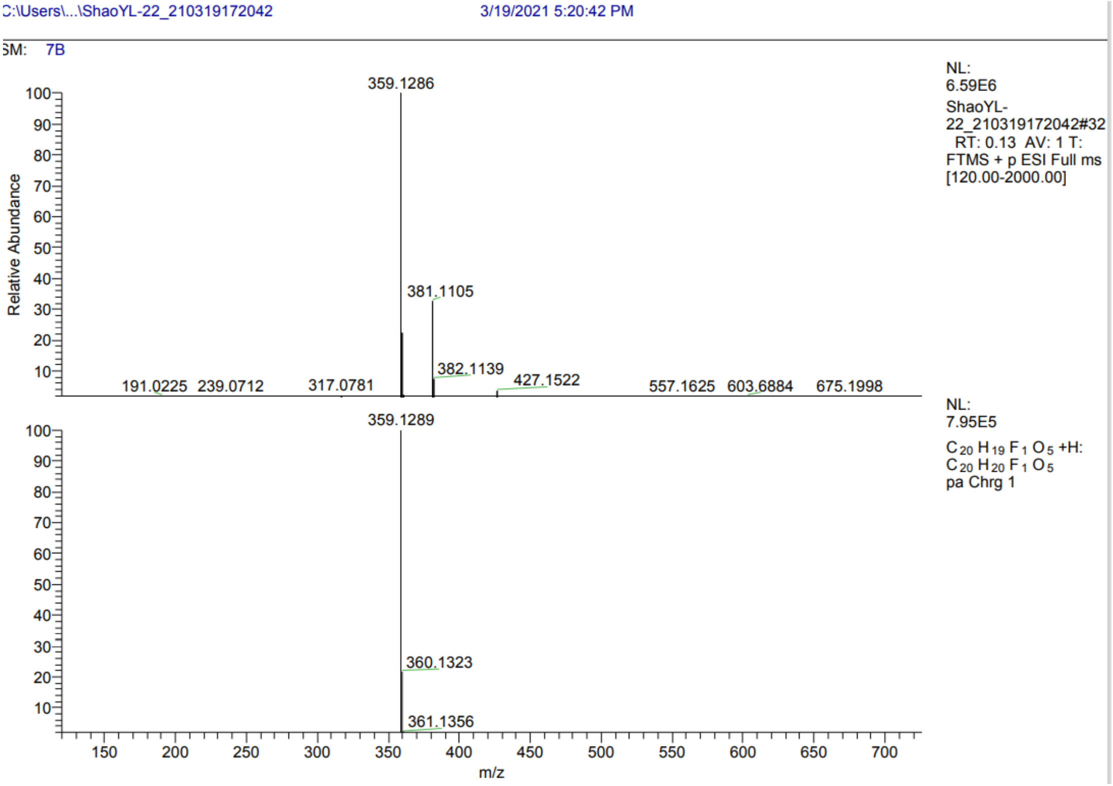

HRMS of **4n**

(15) ^{1}H、^{13}C NMR and HRMS spectra of **4o**

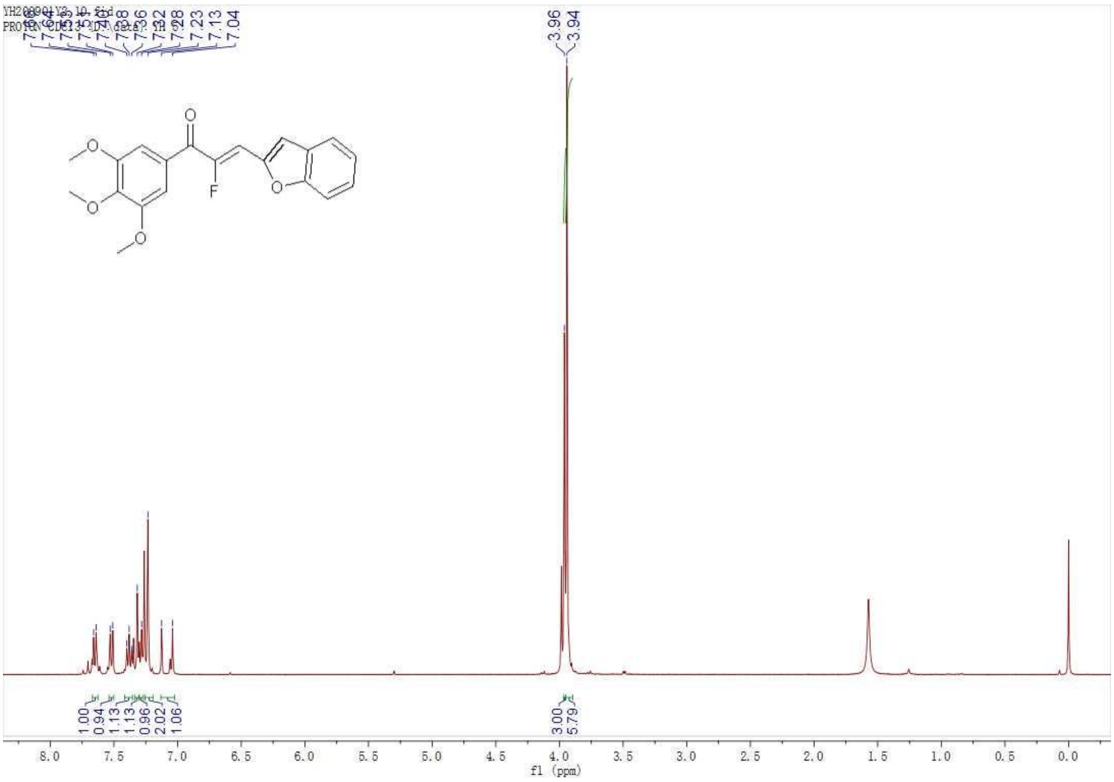

^{1}H NMR of **4o**

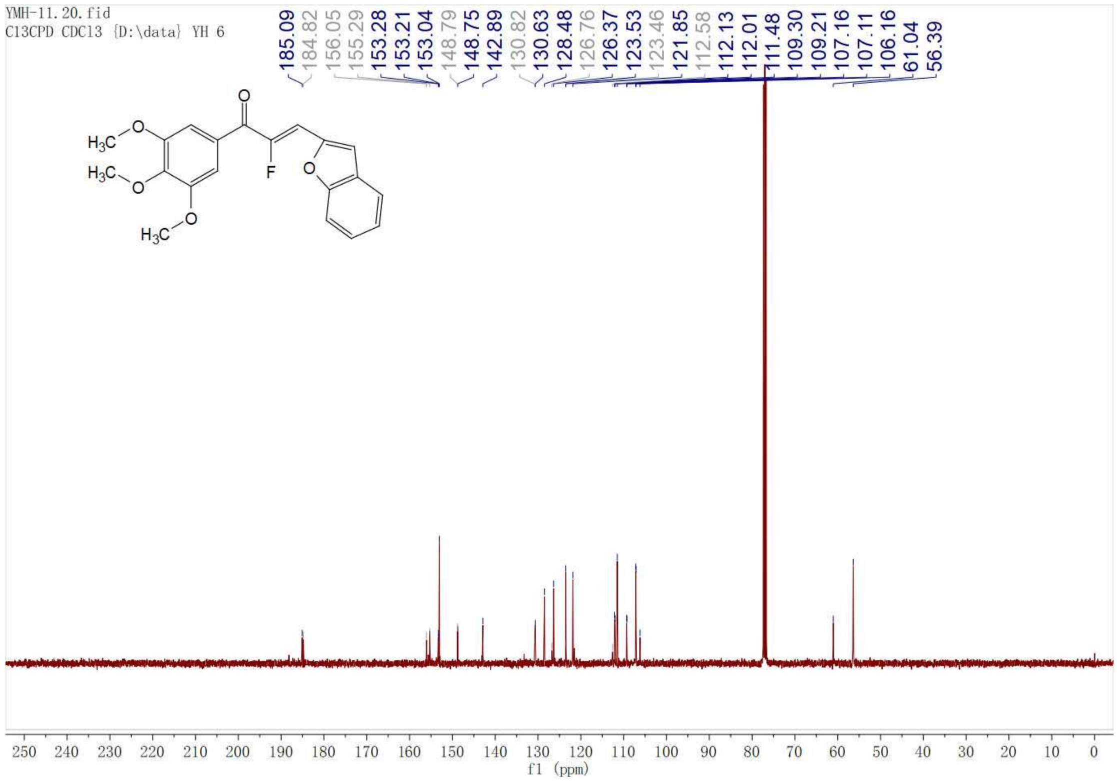

^{13}C NMR of **4o**

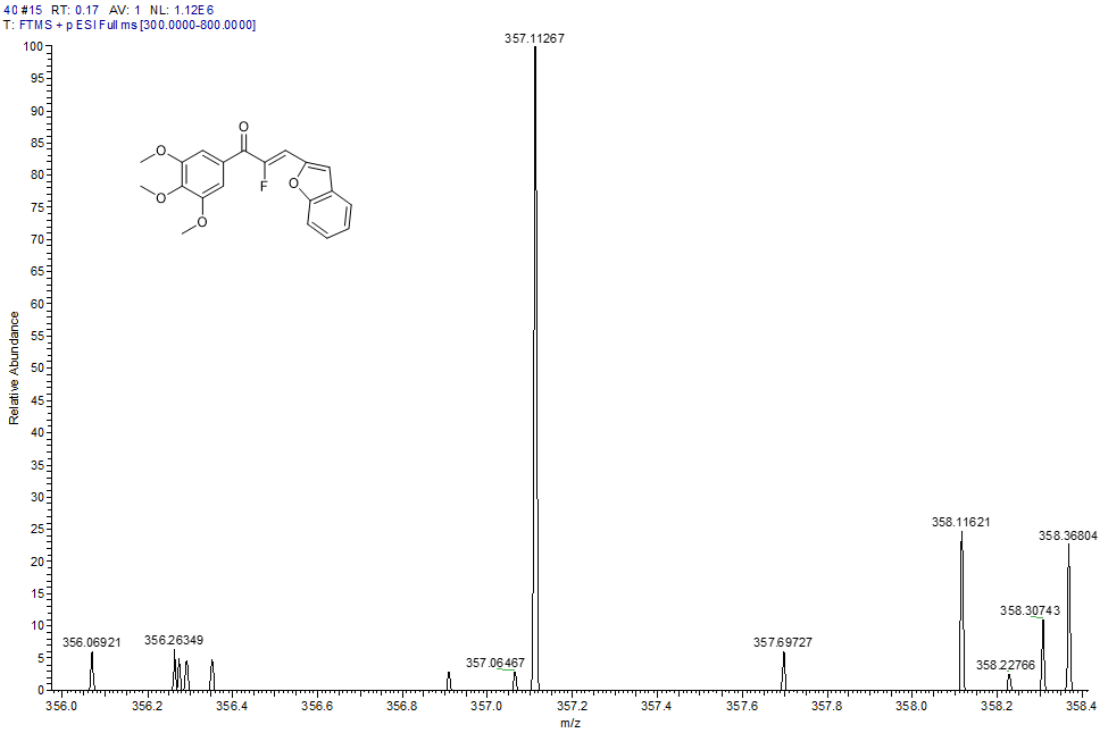

HRMS of **4o**

(16) ^{1}H、^{13}C NMR and HRMS spectra of **4p**

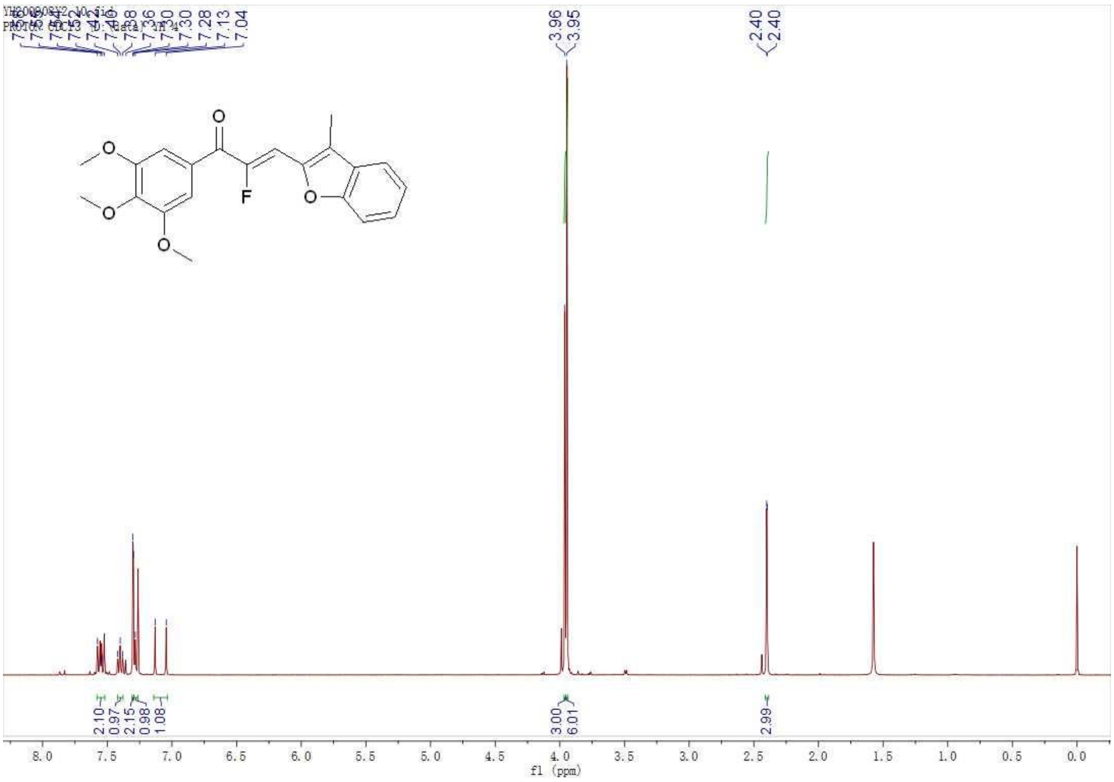

^{1}H NMR of **4p**

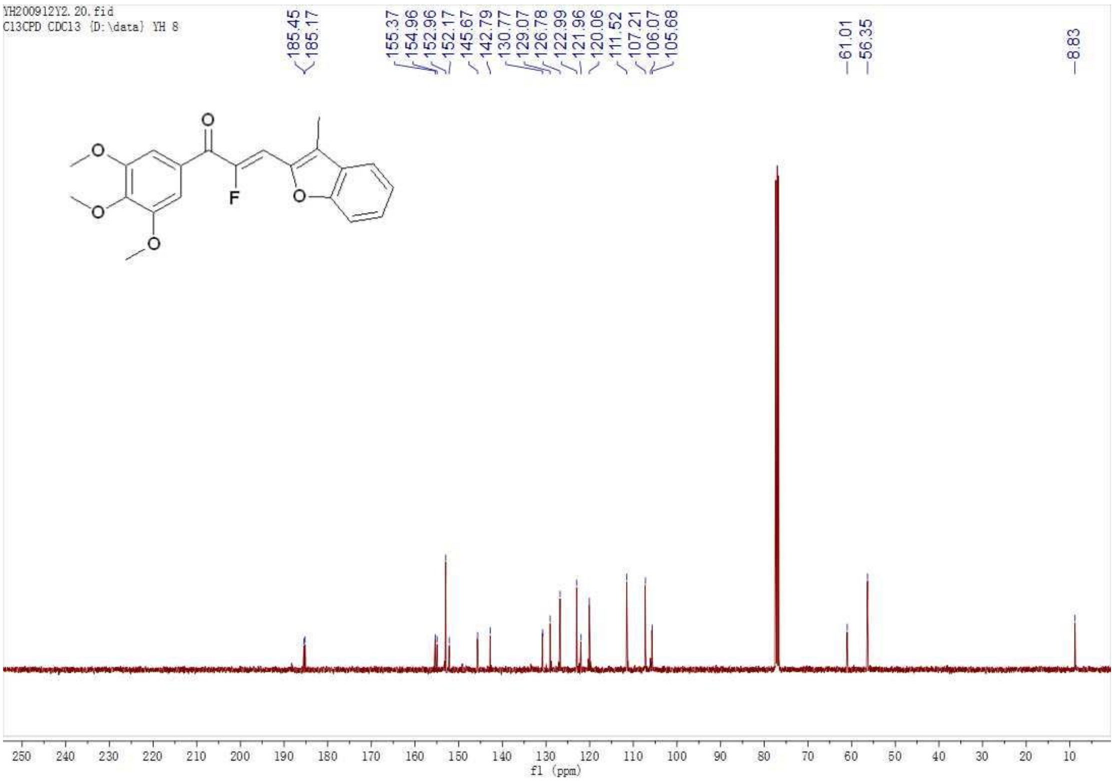

^{13}C NMR of **4p**

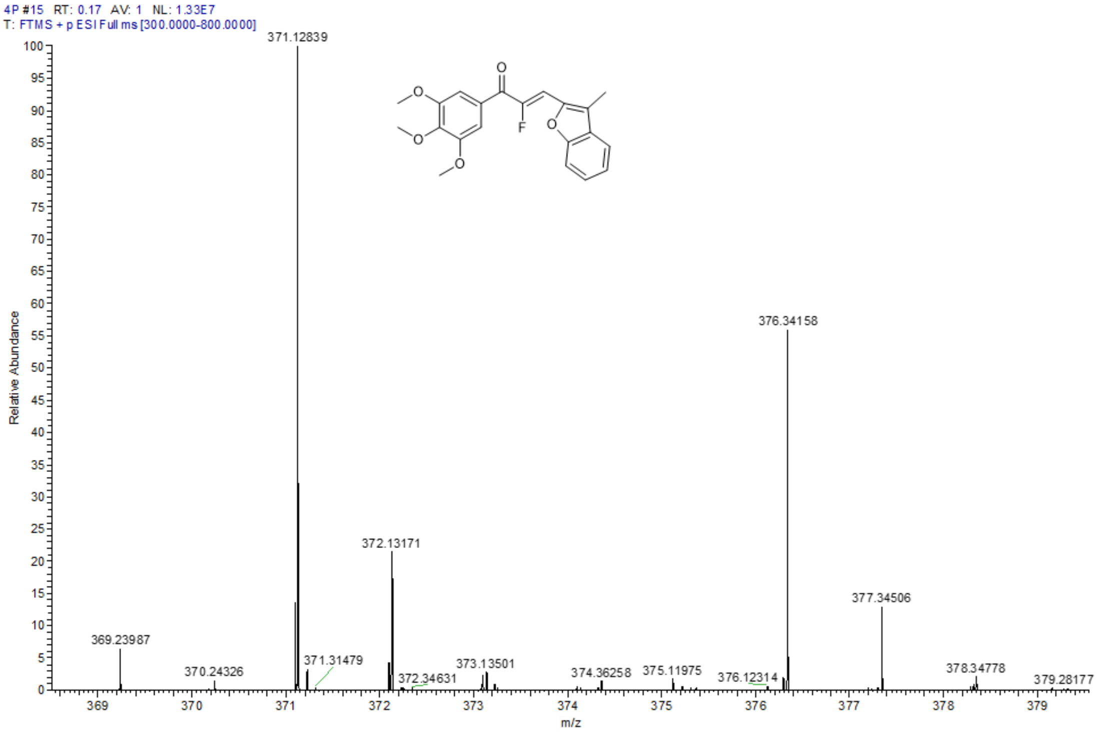

HRMS of **4p**

(17) ^{1}H、^{13}C NMR and HRMS spectra of **4q**

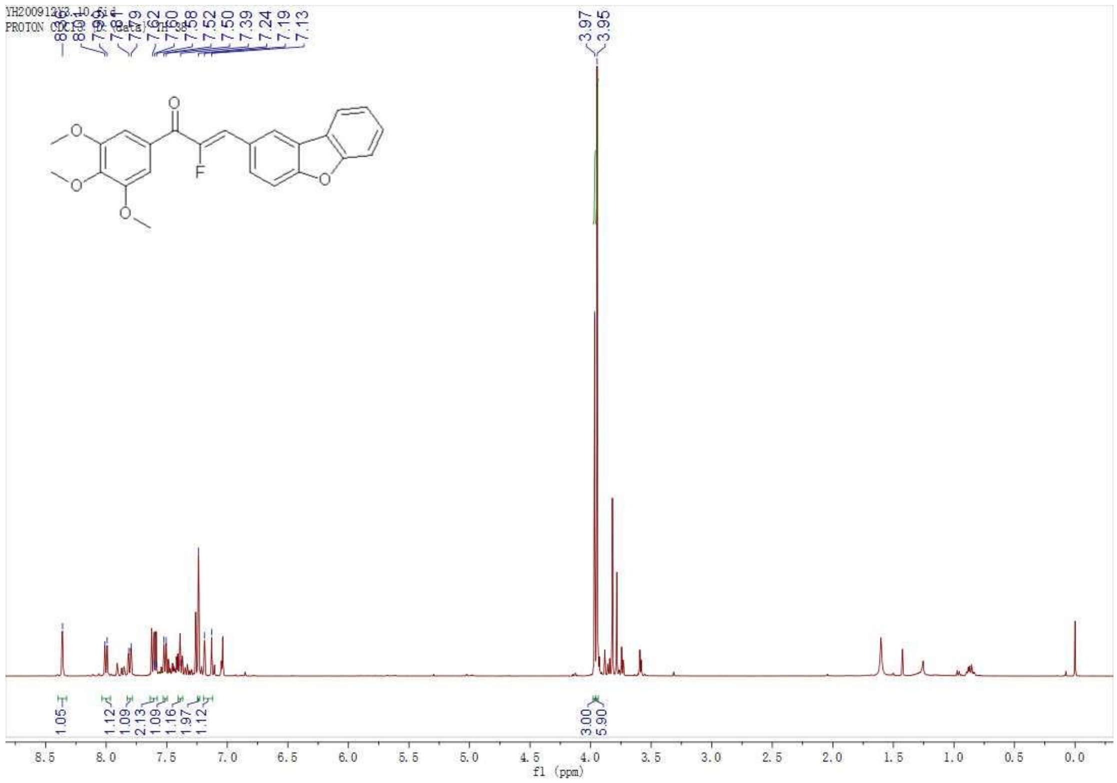

^{1}H NMR of **4q**

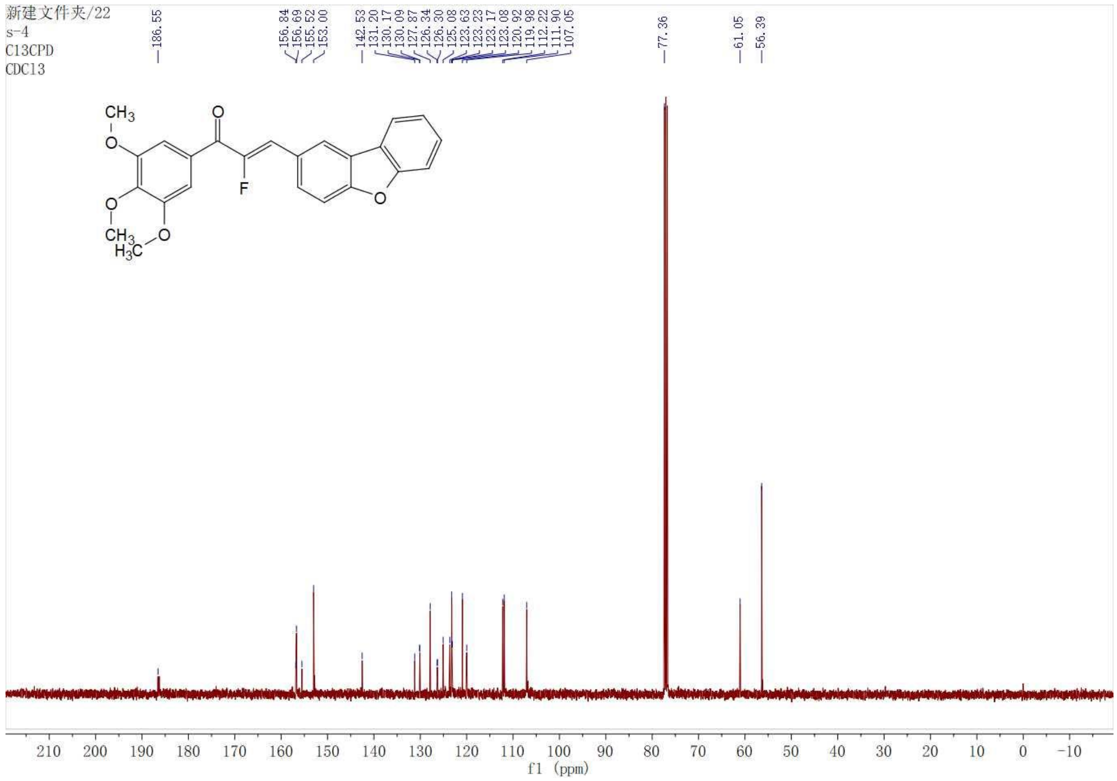

^{13}C NMR of **4q**

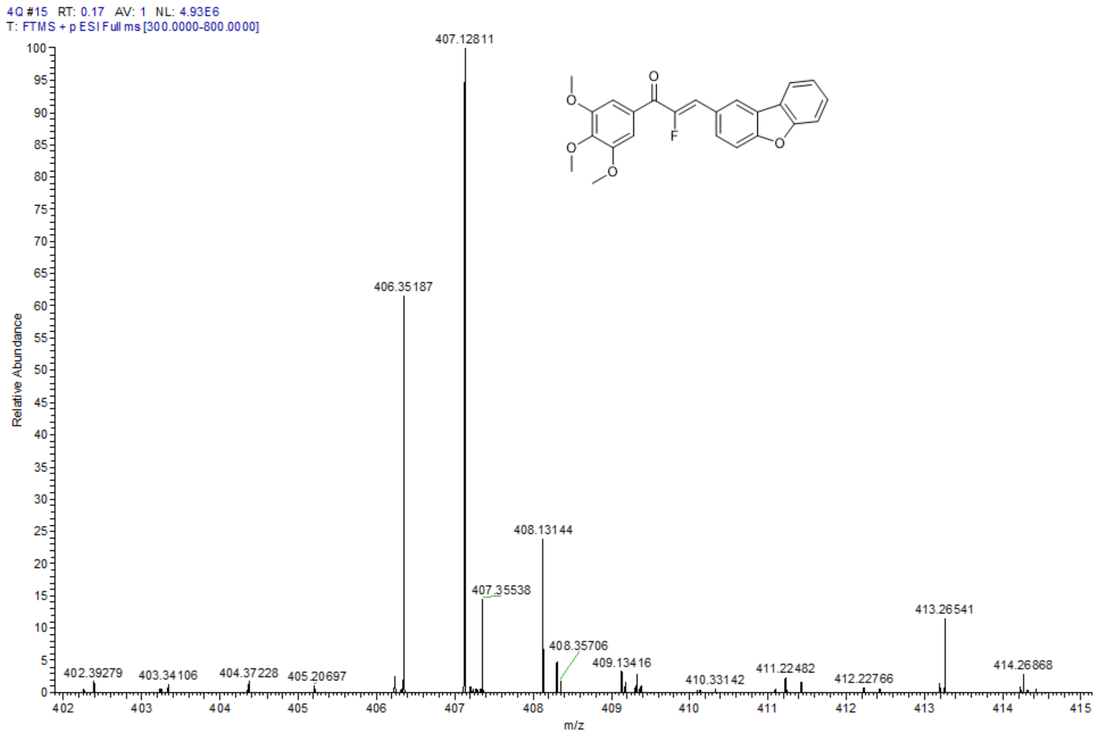

HRMS of **4q**

(18) ^{1}H、 ^{13}C NMR and HRMS spectra of **4r**

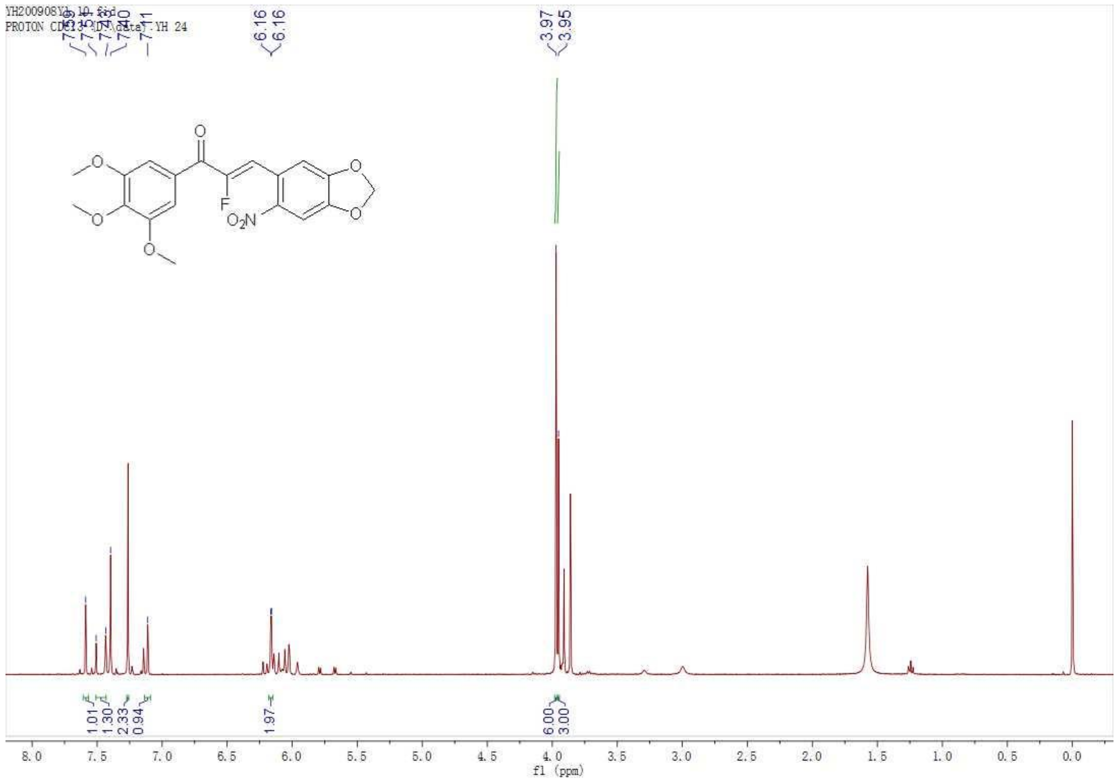

^{1}H NMR of **4r**

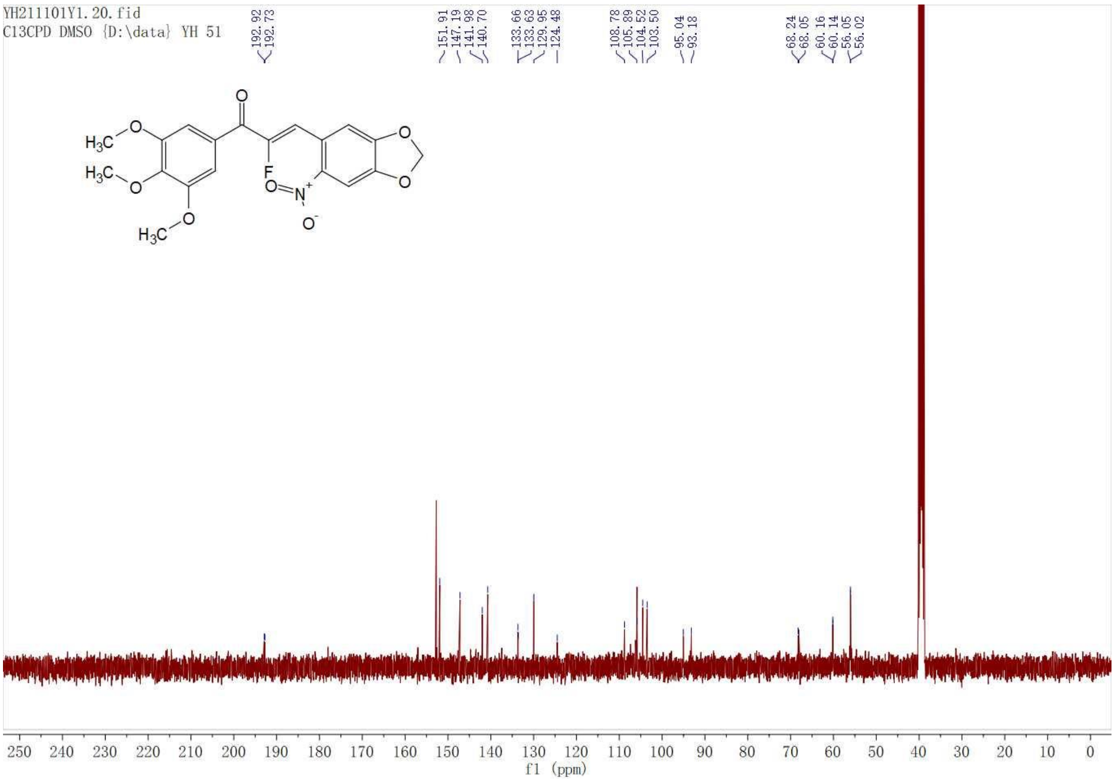

^{13}C NMR of **4r**

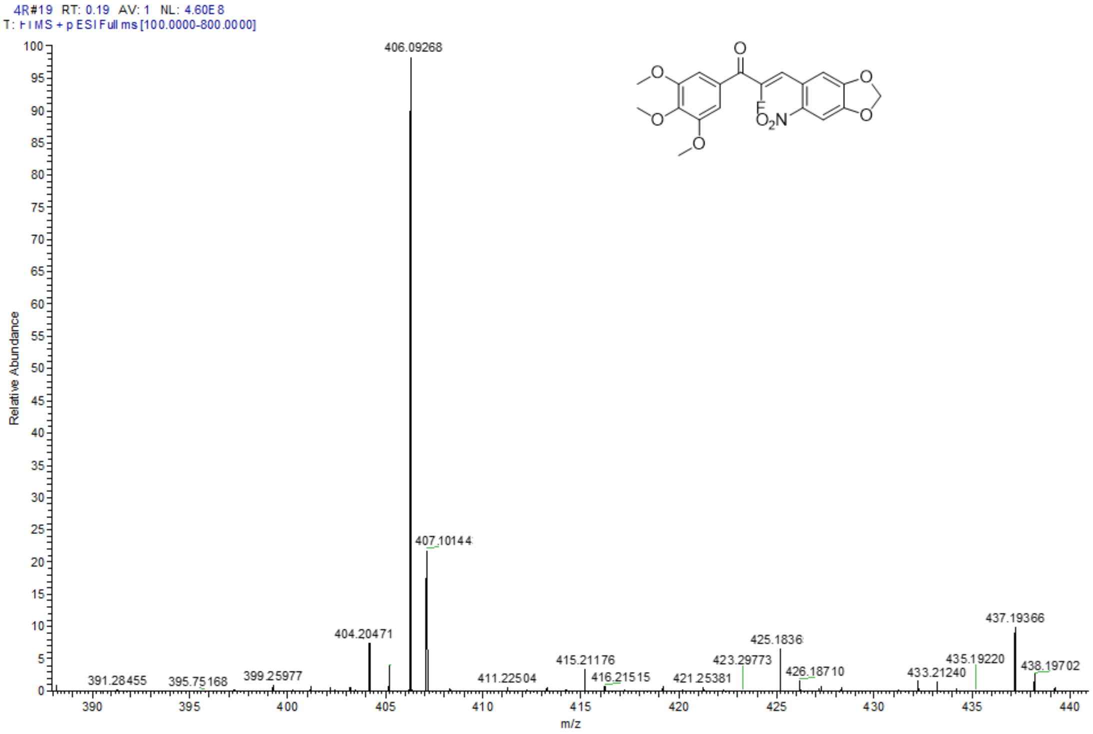

HRMS of **4r**

(19) ^{1}H、^{13}C NMR and HRMS spectra of **4s**

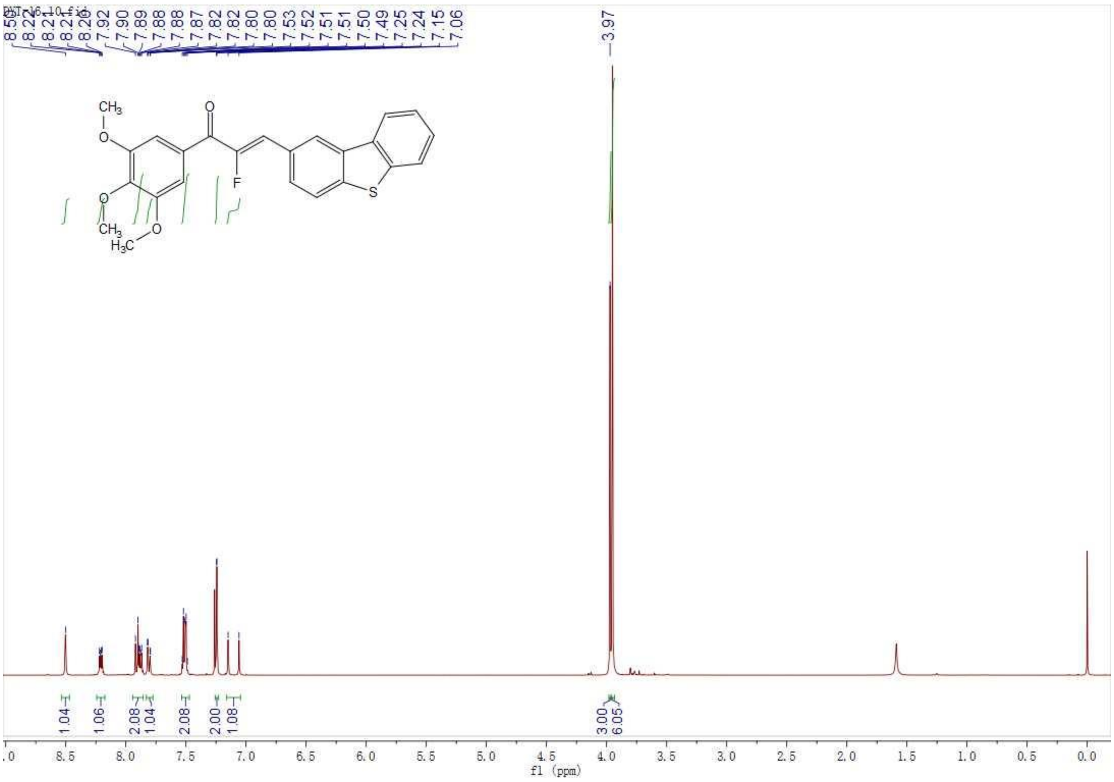

^{1}H NMR of **4s**

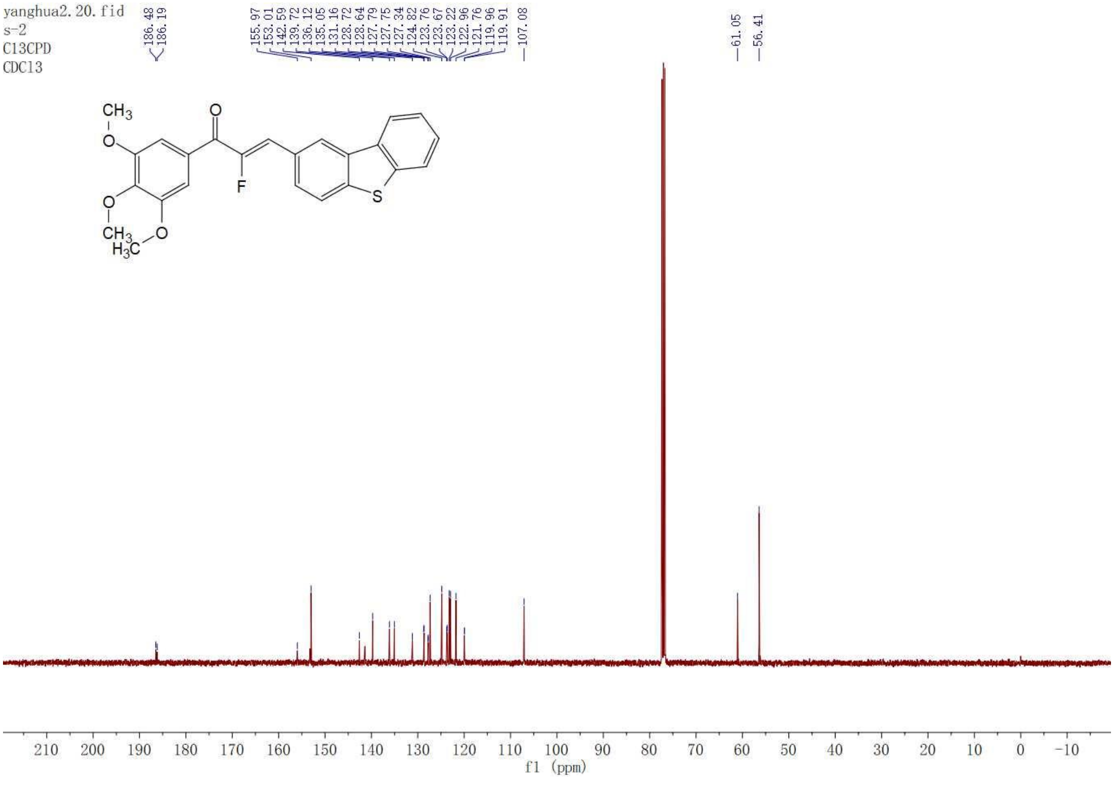

^{13}C NMR of **4s**

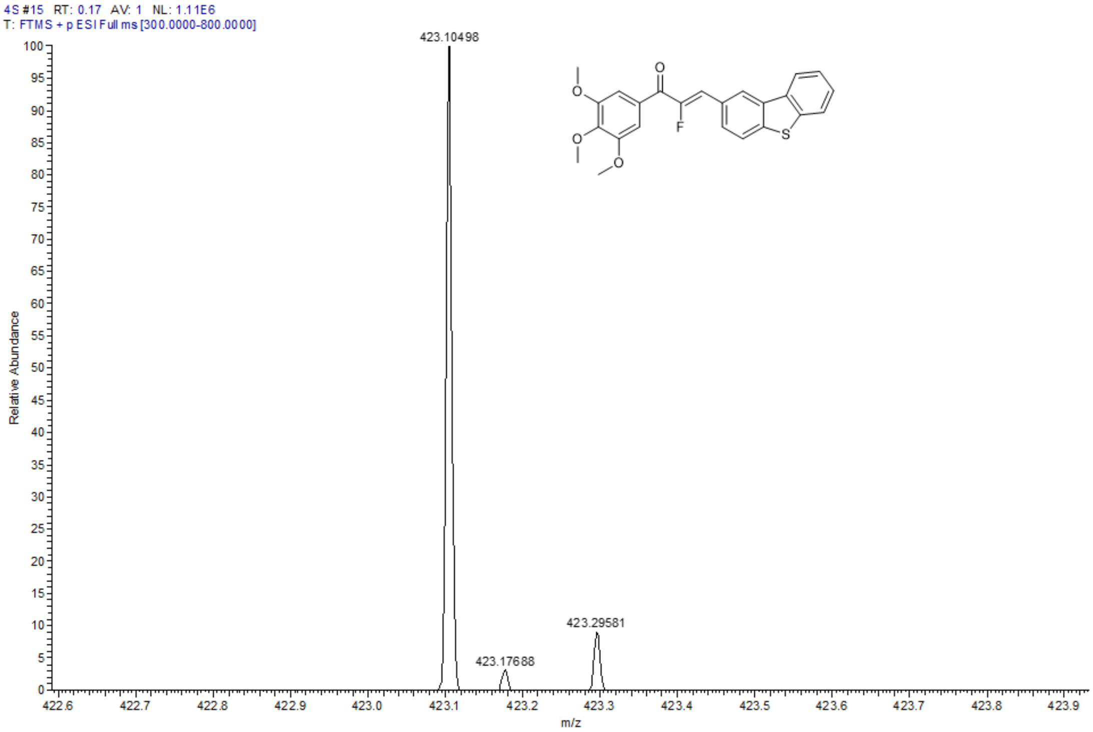

HRMS of **4s**

(20) ^{1}H、^{13}C NMR and HRMS spectra of **4t**

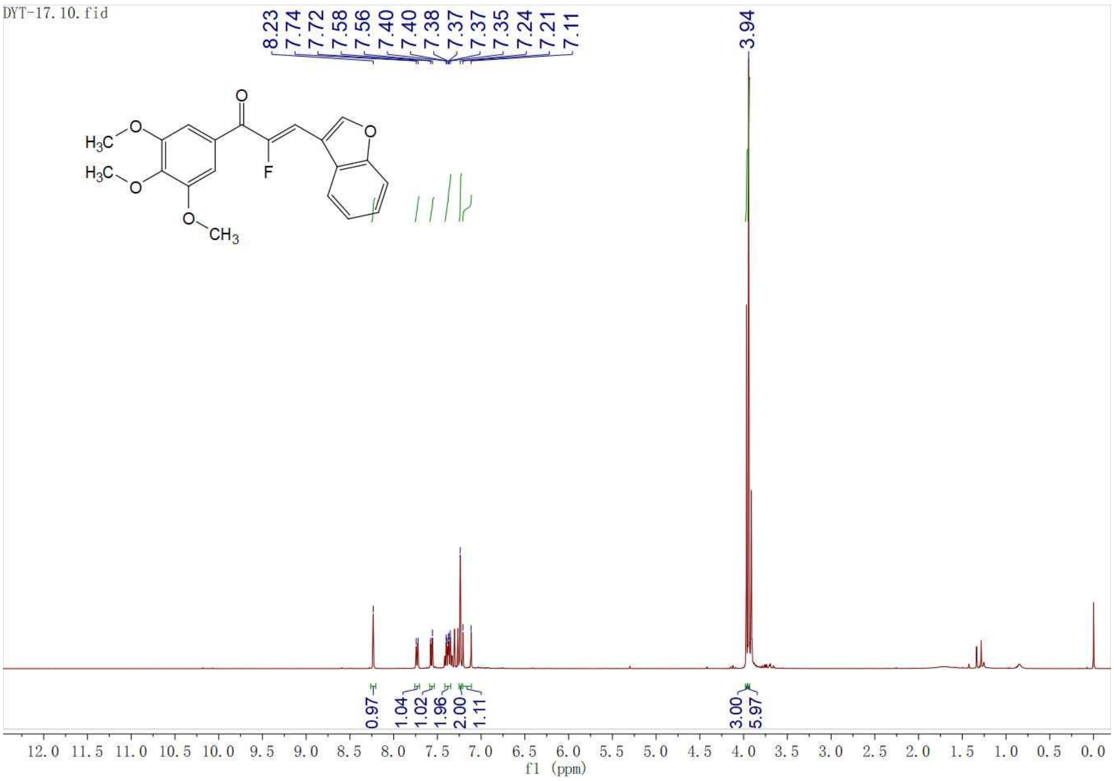

^{1}H NMR of **4t**

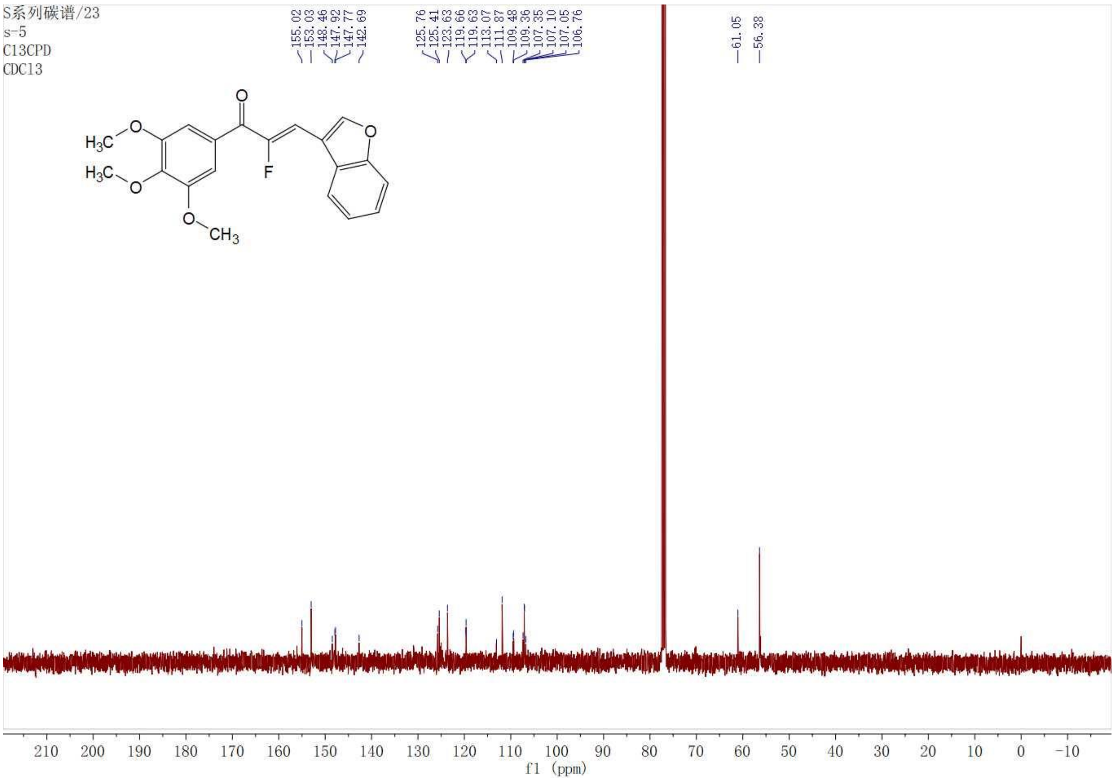

^{13}C NMR of **4t**

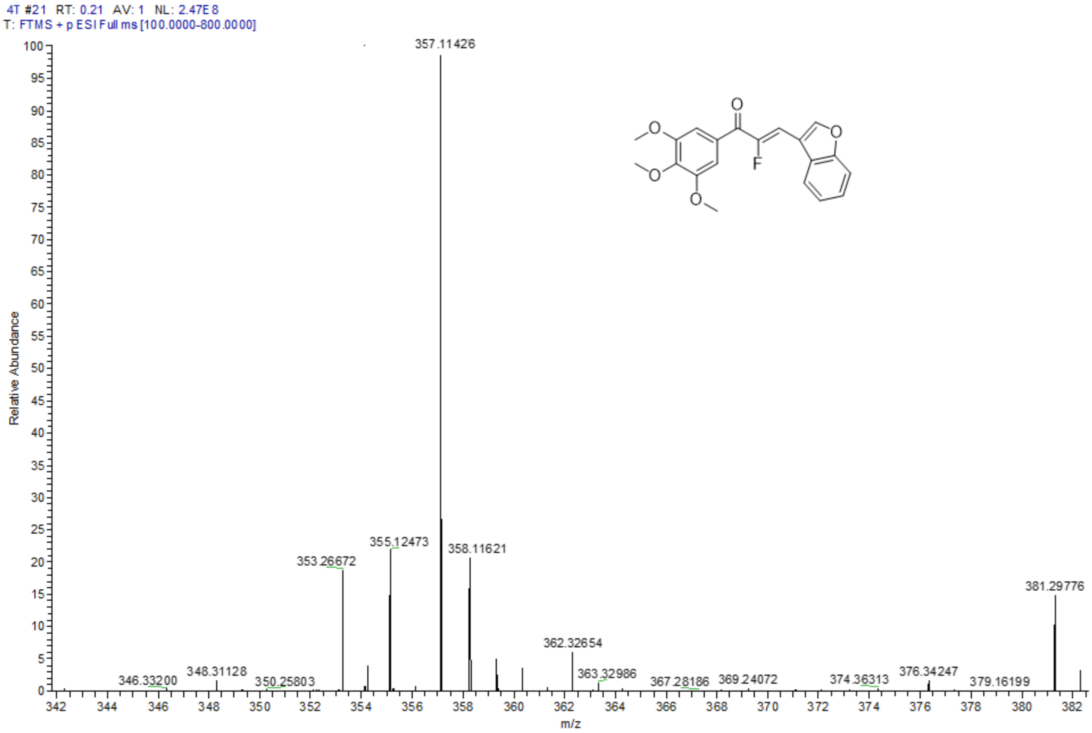

HRMS of **4t**

2. Cell arrest western blot assays films of compound 4c(48h)

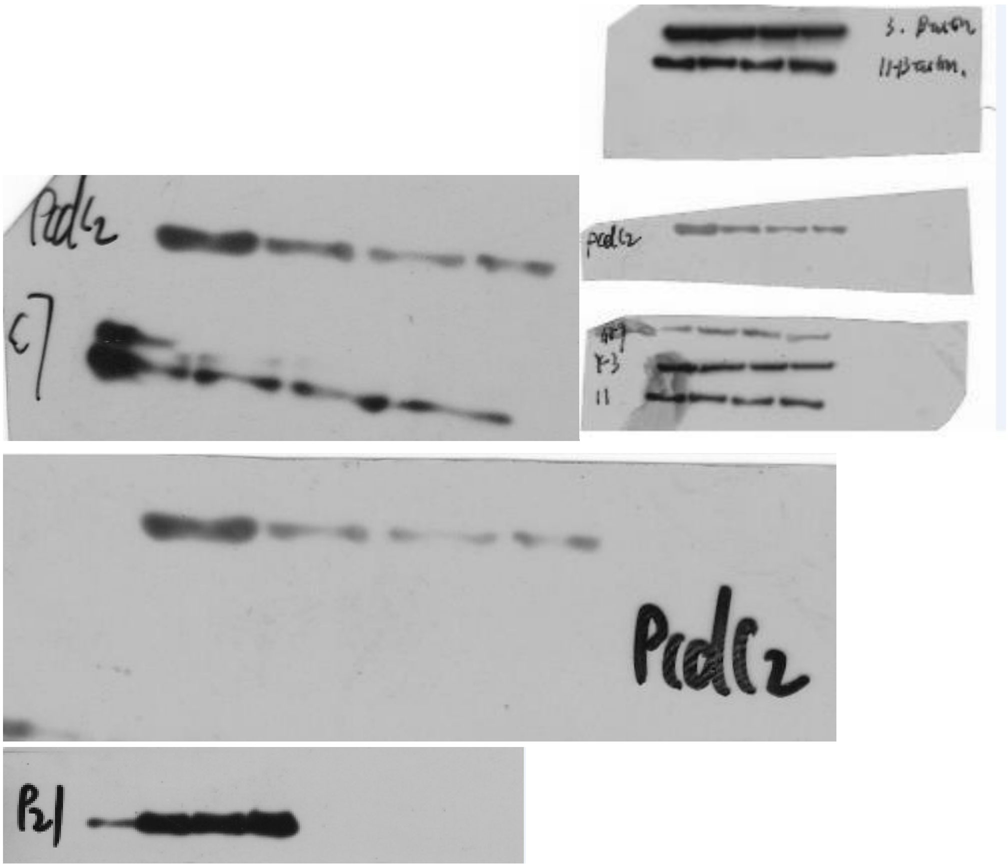

3. Cell apoptosis western blot assays films of compound 7m

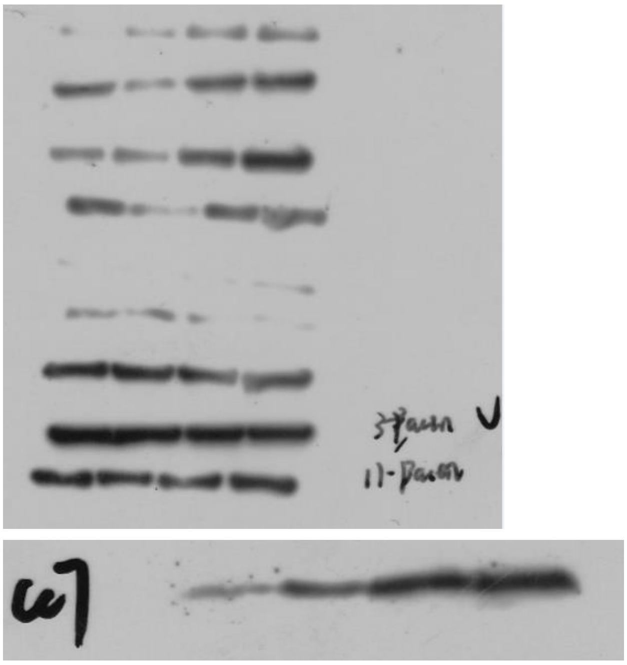

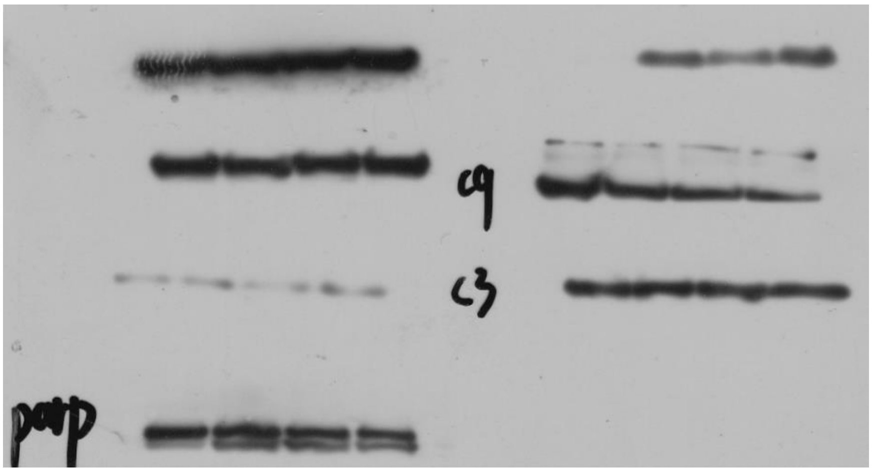
c9
c3
parp

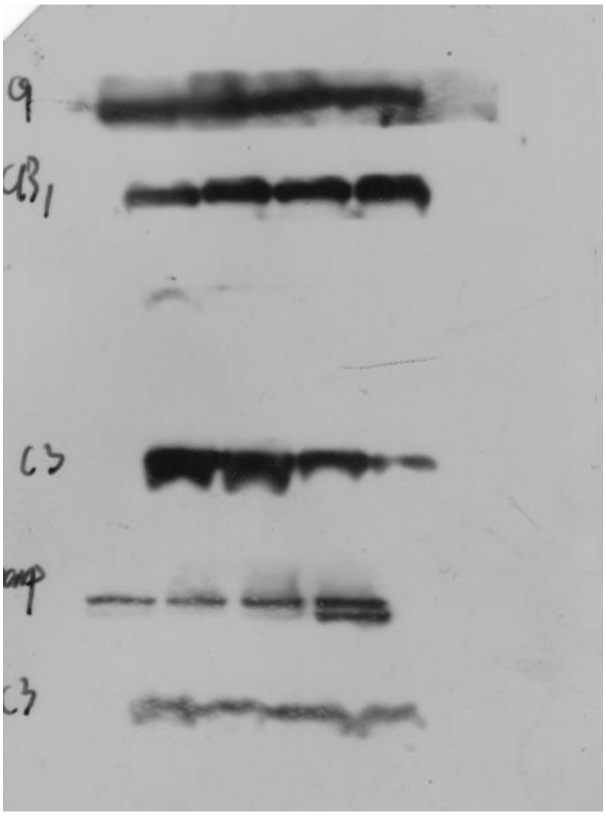
c9
CB1
c3
c3
